# Evaluation of Automated Magnetic Bead–Based DNA Extraction for Detection of Short Tandem Repeat Expansions With Nanopore Sequencing

**DOI:** 10.1002/jcla.25029

**Published:** 2024-03-20

**Authors:** Helene Faust, Patricia Duffek, Julia Hentschel, Denny Popp

**Affiliations:** ^1^ Institute of Human Genetics University of Leipzig Medical Center Leipzig Germany

**Keywords:** blood storage, DNA extraction, DNA integrity, nanopore sequencing, repeat expansion

## Abstract

**Background:**

Long‐read technologies such as nanopore sequencing provide new opportunities to detect short tandem repeat expansions. Therefore, a DNA extraction method is necessary that minimizes DNA fragmentation and hence allows the identification of large repeat expansions. In this study, an automated magnetic bead–based DNA extraction method and the required EDTA blood storage conditions as well as DNA and sequencing quality were evaluated for their suitability for repeat expansion detection with nanopore sequencing.

**Methods:**

DNA was extracted from EDTA blood, and subsequently, its concentration, purity, and integrity were assessed. DNA was then subjected to nanopore sequencing, and quality metrics of the obtained sequencing data were evaluated.

**Results:**

DNA extracted from fresh EDTA blood as well as from cooled or frozen EDTA blood revealed high DNA integrity whereas storage at room temperature over 7 days had detrimental effects. After nanopore sequencing, the read length N50 values of approximately 9 kb were obtained, and based on adaptive sampling of samples with a known repeat expansion, repeat expansions up to 10 kb could be detected.

**Conclusion:**

The automated magnetic bead–based DNA extraction was sufficient to detect short tandem repeat expansions, omitting the need for high‐molecular‐weight DNA extraction methods. Therefore, DNA should be extracted either from fresh blood or from blood stored in cooled or frozen conditions. Consequently, this study may help other laboratories to evaluate their DNA extraction method regarding the suitability for detecting repeat expansions with nanopore sequencing.

## Graphical abstract

This study may help other laboratories to evaluate their DNA extraction method regarding the suitability for detecting repeat expansions with nanopore sequencing. We evaluated an automated magnetic bead–based DNA extraction as suitable for repeat expansion detection when the blood samples have been short‐term stored in cooled or frozen condition. Furthermore, we showed the DNA integrity number as a good predictor for the required read length distribution for repeat expansion detection.

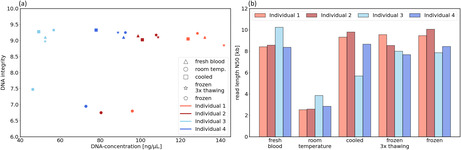

## 1 Introduction

More than 50 short tandem repeat (STR) expansion disorders are known, which are caused by pathogenic expansions of repetitive DNA sequences [1]. These STR expansions are composed of repetitive motifs of up to 9 bp and can encompass in total a size up to 40 kb, such as in the gene *CNBP* causing myotonic dystrophy [2]. STRs are located in coding regions, intronic, in the 3′ and 5′ UTR region, and in noncoding RNA. Even though several bioinformatics tools, such as ExpansionHunter [3], enable the detection of STR expansions in short‐read sequencing data, there are limitations regarding the precise detection and size estimation of large STR expansions, such as interruptions within a repeat, GC‐rich regions and epigenetic modifications [4].

Nanopore sequencing as one option for long‐read sequencing overcomes these limitations. Its use was already demonstrated for the validation of new STR expansions that were initially identified in short‐read sequencing data [5, 6]. In order to characterize several known STR loci with targeted nanopore sequencing simultaneously, at least two different approaches have been developed [7, 8]. First, Stevanovski et al. [7] applied adaptive sampling to detect and analyze 37 STR expansion loci. Adaptive sampling allows targeted enrichment using a real‐time data analysis by recognition and acceptance or rejection of single DNA fragments through a programmable target selection [9, 10]. Second, Erdmann et al. [8] used a nanopore Cas9‐targeted long‐read sequencing approach for STR expansion detection.

Using long‐read sequencing requires a dedicated workflow from wet to dry laboratories. In particular, a method for DNA extraction that allows extensive read lengths is needed. Therefore, commercially available high‐molecular‐weight kits and several other DNA extraction protocols have been evaluated for specific applications [11, 12]. For STR expansion detection with nanopore sequencing, Stevanowski et al. [7] used two commercially available high‐molecular‐weight DNA extraction kits, a salting‐out precipitation DNA purification and a silica‐based DNA purification, whereas Erdmann et al. [8] used a conventional automated magnetic bead–based DNA extraction method. In short, the detection of STR expansions requires DNA fragments of sufficient length whereas DNA integrity depends on the method of DNA isolation and on the preservation of the blood sample.

In a clinical setting, blood samples might be taken at a different geographically location as the DNA extraction and subsequent analyses take place. This might require even the shipping of blood samples. However, storage conditions are usually not well controlled, especially regarding temperature during the shipment as blood samples are usually shipped without any cooling. Hence, these samples are exposed to ambient temperatures. Prolonged storage even at room temperature has been shown to be detrimental in terms of DNA fragmentation [13]. Furthermore, blood samples might be stored under freezing conditions as long‐term storage. As subsequent analyses might be performed on those frozen samples, they undergo several freezing and thawing cycles that can also affect the DNA integrity.

In this study, the suitability of an automated magnetic bead–based DNA extraction method (MagCore HF 16 Plus II Nucleic Acid Extractor) for the detection of STR expansions with nanopore sequencing was investigated. Moreover, since EDTA is the most common anticoagulant for blood storage for DNA extraction in routine diagnostics, the effect of storage conditions of EDTA blood samples on DNA and nanopore sequencing quality was assessed. Furthermore, the DNA integrity number (DIN) was evaluated as a predictor for read length distribution. In order to investigate the feasibility of the DNA extraction method for the detection of STR expansions, DNA samples from individuals with known STR expansions were sequenced.

## 2 Materials and Methods

### 2.1 DNA Extraction and Quality Measurement

Genomic DNA (gDNA) extraction from EDTA whole blood was performed using the MagCore HF 16 Plus II Nucleic Acid Extractor, which performs an automated magnetic bead–based DNA extraction within 49 min for a batch of up to 16 EDTA blood samples. Starting from 400 μL of EDTA blood samples, 100 μL of DNA elution volume was obtained using the Genomic DNA Whole Blood Kit (RBC Bioscience) and 101 cartridge (RBC Bioscience) according to the manufacturer's instructions. gDNA was extracted four times from each blood sample. DNA concentrations and purity (260/280 ratio) were measured in triplicates with a Tecan Infinite 200 Microplate Reader using the absorbance mode. gDNA samples were long‐term stored at 4°C and EDTA blood samples at −20°C. Using a 4150 TapeStation System (Agilent) with the Genomic DNA ScreenTape assay and the TapeStation analysis software, the DIN was calculated. The DIN reproduces the fragmentation of a gDNA sample to a scale of DIN 1–10, where 1 is the highest DNA degradation and 10 is the highest DNA integrity [14]. Mean values and standard deviations of measured DNA concentrations, 260/280 ratio, and the DIN values were calculated.

### 2.2 Nanopore Sequencing

For nanopore sequencing of several gDNA samples simultaneously, the library was prepared from 1 μg DNA of each sample using the NEBNext Companion Module (New England BioLabs) and the 24 barcoding library prep kit (SQK‐NBD112.24 or SQK‐NBD114.24) according to the standard protocol of Oxford Nanopore Technology (ONT) with the following adjustments. Incubation time of the end‐prep reaction was increased to 30 min at 20°C and 30 min at 65°C. Incubation time of adapter ligation reaction was adjusted to 60 min at room temperature. The pool of barcoded probes (~200 ng) was loaded onto a MinION flow cell type R9.4.1 or R10.4.1 and sequenced on a MinION device Mk1b (ONT). Thereby, 3.5 and 4.5 Gb of raw sequencing data was obtained, respectively (see Table S6).

For adaptive sampling, the library was obtained from 2 μg gDNA of each sample using the NEBNext Companion Module (New England BioLabs) and the ligation sequencing kit (SQK‐LSK110) according to the ligation‐sequencing gDNA protocol of ONT with the same adjustments as mentioned above. Each sample (~300 ng) was loaded onto a MinION flow cell type R9.4.1 and sequenced on a MinION device Mk1b (ONT) using adaptive sampling. Therefore, a BED file format was used containing known repeat expansion loci and ~50 kb flanking region upstream and downstream of each locus (see Appendix S1). The known repeat expansion loci were taken from the STRipy Database of Short Tandem Repeats [15]. Flow cells were washed after 24 h using the wash kit EXP‐WSH004 (ONT) according to the manufacturer's instructions after which sequencing was continued by reloading of ~300 ng of the same library (for details about the sequencing runs, see Table S1).

Raw data (fast5 files) were basecalled and de‐multiplexed if required by guppy v6.1.2 or v6.4.2 (ONT) with enabled read splitting and adapter as well as barcode trimming. Afterwards, read metrics were calculated using NanoPlot v1.4.0 and NanoComp v1.23.1 [16]. For the sequencing runs with adaptive sampling, the read length N50 values were calculated using only the on‐target reads without rejected reads. Reads were then mapped against the human reference genome hg38 using minimap2 v2.17‐r941 [17]. Repeat loci of interest were assessed manually using the Integrative Genomics Viewer [18].

## 3 Results

### 3.1 Effect of DNA Integrity on Nanopore Sequencing Results

In order to measure the DNA quality and its influence on nanopore sequencing, five EDTA blood samples were obtained from four healthy individuals each. DNA was immediately isolated from one sample per individual. The other blood samples were stored either at room temperature, cooled (4°C), frozen (−20°C), or frozen (−20°C) with three times thawing (4°C) over a period of 7 days until DNA extraction. Seven days after the respective DNA extraction, DNA concentrations and DIN values were determined for each sample. Subsequently, nanopore sequencing was performed for one DNA sample per storage condition of each individual (20 DNA samples in total).

**FIGURE 1:**
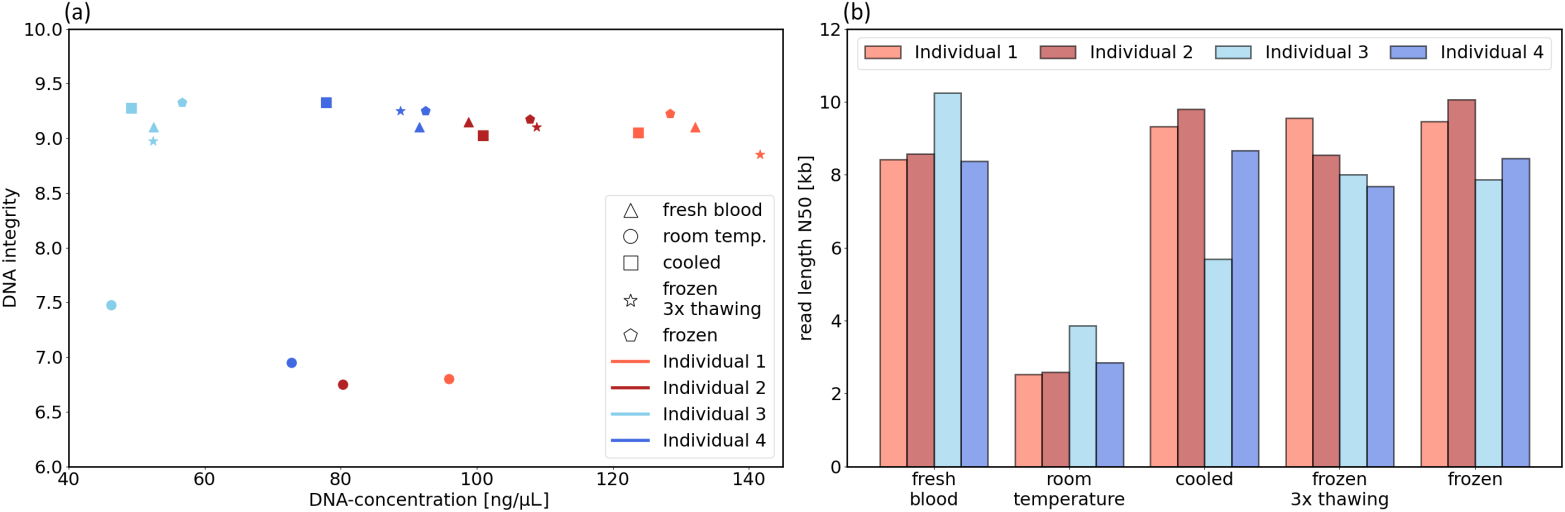
(a) DNA concentration and integrity number (DIN) and (b) read length N50 values of nanopore sequencing of the DNA of the four individuals isolated from fresh blood, after storage at room temperature, cooled (4°C), and frozen (−20°C) with three times thawing (4°C).

The range of DNA concentrations varied strongly between the individuals ranging from 53 to 132 ng/μL (fresh blood), whereas DNA concentrations of blood were considerably lower after storage at room temperature than in fresh, cooled, or frozen conditions (see Figure 1a and Table S2). DNA purity as 260/280 ratio was confirmed to be between 1.8 and 1.9 for all samples (see Table S3). The DIN values were in a similar range for all individuals depending on the storage condition of the blood sample and seemed to be independent of the DNA concentration (see Figure 1a and Table S4). DNA samples obtained from fresh, cooled, or frozen blood showed high DIN values with a minimum of 9.0 to a maximum of 9.3 overall. In contrast, DNA samples obtained from blood stored at room temperature showed lower DIN values with a minimum of 6.8 and a maximum of 7.5. A similar effect was observed for the read length distribution of nanopore sequencing (see Figure 1b, Tables S2–S4, Appendices S2 and S3). The DNA extracted from fresh, cooled, or frozen blood showed a read length N50 with a minimum of 5.7 kb and a maximum of 10.2 kb (mean overall: 8.7 kb). After storage at room temperature, much lower N50 values were measured, with a minimum of 2.5 kb and a maximum of 3.8 kb (mean: 3 kb). The mean read quality was not affected by storage conditions (see Table S5).

In order to evaluate the DIN as a predictor for the read length N50 values of nanopore sequencing, 24 DNA samples from our in‐house collection of samples were collected with the varying DIN values and nanopore sequencing to determine the read length N50 value was performed.

**FIGURE 2:**
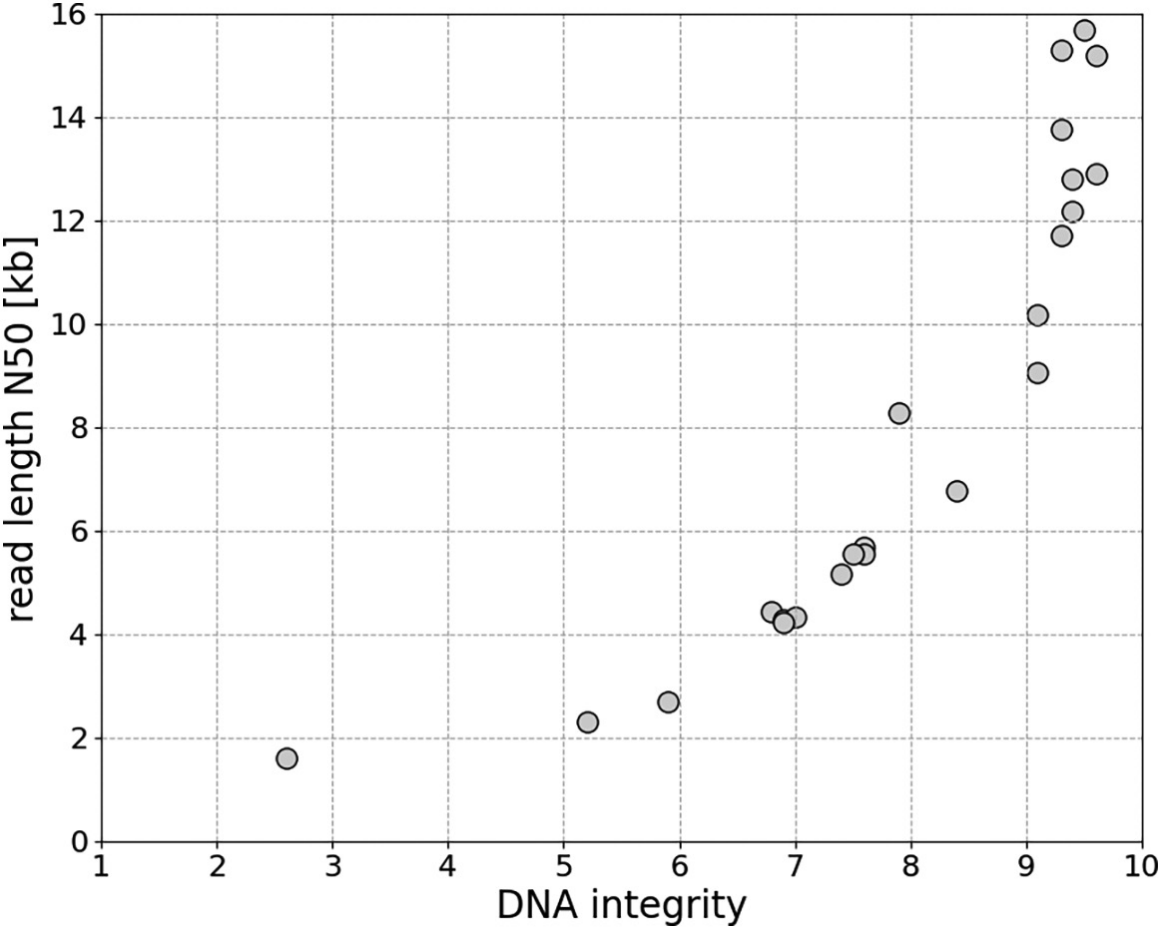
Dependency of read length distribution as N50 value and DNA integrity number (DIN).

Thereby, a strong dependency of the DIN and N50 values was observed. As the DIN value increases, so do the N50 values, although there is a restriction on predicting the N50 values larger than approximately 9 kb (see Figure 2, Table S6, Appendices S4 and S5).

### 3.2 Detection of STR Expansions

After confirming high quality of the extracted DNA with the automated magnetic bead–based method, the reproducibility of the measured DNA quality in previously extracted samples of patients was investigated as well as the feasibility of detecting STR expansions. Therefore, three DNA samples from patients with known STR expansions in the genes *FXN*, *CNBP*, and *C9ORF72*, respectively, were selected. The STR expansion counts of the samples were determined as part of routine diagnostics in other laboratories, independently of this study (see Table 1). The DIN values of the DNA samples were measured, followed by the preparation of nanopore libraries and sequencing with adaptive sampling using a template of known repeat expansion loci.

**TABLE 1:** STR expansion detection in DNA samples with known repeat expansions.

Gene	Motif	DNA and sequencing quality			Expanded allele		
		DIN	N50 (kb)	Coverage	Read count	Nanopore (repeat count)	Reference (repeat count)
*FXN*	GAA	9.4	16.4	27×	10	404–1000	900^a^
*C9ORF72*	GGGGCC	9.2	13.9	12×	7	484–911	1600–1800^b^
*CNBP*	CCTG	8.5	6.1	16×	2	1502–1504	>75^c^

*Note:* DNA quality is given as the DIN value and sequencing quality as on‐target read length N50 values (without rejected reads) and on‐target sequencing coverage. Details about the expanded allele contain the read count and the repeat counts determined by nanopore sequencing and estimated by a reference method.

^a^ PCR, fragment analysis.

^b^ Southern blot analysis.

^c^ Tetraplet‐primed PCR.

As observed before, the DIN values corresponded with the N50 values of the on‐target reads of the samples (see Table 1 and Appendix S6). The sample with the trinucleotide (GAA) repeat expansion in intron 1 of the gene *FXN* had the highest DIN value and the largest read length distribution of the three samples. The repeat expansion was detected with nanopore sequencing in about a third of the reads. The size of the repeat expansion (404–1000 repeat units) was in the range of the previously determined size (900 repeat units) using PCR and fragment analysis. A high DIN and N50 value was also measured for the sample with the hexanucleotide (GGGGCC) repeat expansion in intron 1 of the gene *C9ORF72*. Half of the nanopore reads showed the repeat expansion, but the repeat count was lower (484–911 repeat counts) than in the previously performed southern blot analysis (1600–1800 repeat units). In contrast, the sample with the tetranucleotide (CCTG) repeat expansion in intron 1 of the gene *CNBP* had a lower DIN than the other two samples and only a N50 value of 6.1 kb. The repeat expansion was only detected in 2 of 16 reads and was larger than the 75 repeat counts as determined by tetraplet‐primed PCR before.

## 4 Discussion

Long‐read sequencing, such as from Oxford Nanopore Technologies (ONT), can overcome the limitations of short‐read sequencing and is increasingly used in a diagnostic setting, especially for repeat expansion detection [19, 20]. Therefore, DNA integrity is crucial for extensive read lengths, enabling the detection of STR expansions that can span several kilobases or even more. In this study, we investigated the feasibility of nanopore sequencing for the detection of STR expansions using an automated magnetic bead–based DNA extraction method. As blood samples were taken from four individuals, we observed strongly varying DNA concentrations, which is probably due to different lymphocyte counts depending on hematocrit, the activity of the immune system, and other individual conditions of the blood lymphocytes. Another observation is that DNA integrity is not influenced by the concentration but rather by the storage conditions. The DIN value was high (>8.8) after DNA extraction of fresh blood as well as cooled or frozen blood for 7 days. However, the DIN value was considerably decreased after storage at room temperature (<7.3). This was also reflected in the read length N50 values of approximately 9 kb for DNA obtained from fresh, cooled, or frozen EDTA blood, in contrast to about 3 kb for DNA obtained from EDTA blood stored at room temperature. The observation of the dependency of short‐term storage conditions on DNA quality agrees with the finding of Schünemann et al. [21], who showed the suitability of DNA isolated from fresh blood and from blood stored at 4°C or −70°C for 7 days for southern blot analysis. Huang et al. [22] investigated the influence of storage of blood samples at room temperature over 15 days and observed a reduced DNA concentration, but no fragmentation of DNA. However, the used DNA electrophoresis with 0.5% agarose gel is only able to dissolve the DNA fragments up to 10 kb, which is insufficient to measure fragment length of higher molecular weight DNA. In contrast, the 4150 TapeStation System (Agilent) used in the present study separates DNA fragments up to 60 kb and reproduces the fragment length as a numeric value, referred as the DIN value [14].

As anticipated in this study, a strong correlation between the DIN and N50 values in nanopore sequencing was observed. However, the DIN values larger than 9.0, which corresponds to the read length N50 values of more than approximately 9 kb in this study, cannot predict the larger N50 values. This might be due to the DIN value reaching its limit to predict the read length distribution at this point. In other applications, the DIN is used for sample selection for nanopore sequencing. Sakamoto et al. [23] utilized only samples with the DIN values of approximately 9.0 for long‐read whole‐genome patterning of cultivated cancer cell lines using enzymatic base conservation and nanopore sequencing. Jain et al. [24] used samples with the DIN values larger than 7.0 to sequence and assemble a reference genome for a human cell line with nanopore. As we observed a strong correlation between the DIN values and the N50 values, the DIN values determined prior to library preparation for nanopore sequencing can be used to identify samples with insufficient DNA integrity. A possibility to increase DNA integrity is a dedicated clean‐up step to eliminate short fragments. Another mean to eventually increase the read length N50 values could be a shearing of the DNA with a high integrity. This is counter‐intuitive, but apparently unsheared DNA tends to fragment stronger than sheared DNA during library preparation [25]. However, including a short fragment elimination and/or shearing step prior to library preparation increases the hands‐on time. Accordingly, measuring the DIN value to reduce the number of samples that have to undergo further sample preparation steps would be of great advantage.

Furthermore, we wondered whether the DIN value is applicable for sample selection for STR expansion detection. Using adaptive sampling to detect repeat expansions, Stevanowski et al. [7] sheared the DNA to 15 kb and measured the N50 values of 12.5 kb of the on‐target reads. We also used adaptive sampling to detect the known STR expansions in samples that have previously been extracted with our magnetic bead–based DNA extraction. The expected STR expansions were detected in all three samples. In both samples with a DIN value higher than 9.0, the N50 value of the on‐target reads was 13.9 and 16.4 kb (see Table 1), respectively, thereby in the range of the N50 values that Stevanovski et al. [7] measured. In these samples, the STR expansion was detected in one sample in a third of the reads and in the other in half of the reads. One would expect to observe the STR expansion in half of the reads in case of a heterozygous expansion. In contrast, the N50 value was only 6.1 kb in the sample with a DIN value of only 8.5 and the expansion was only identified in two of 16 reads, which was also lower than expected. This showcases that expanded alleles do not need to be detected as heterozygous due to technical limitations even though heterozygosity is assumed. Apparently, there is a preference for the shorter allele. This could be due to a bias during library preparation and during the sequencing process. Additionally, reads showing mainly the expanded repeat motif might not map to the reference repeat locus in contrast to reads with the normal allele. Despite these deviations from the expected allele frequency of 50% (heterozygosity), these findings suggest that STR expansions, such as in the genes *CNBP* and *C9ORF72*, can be detected reliably in samples with DIN values larger than approximately 9.0.

It has to be noted that we detected a lower repeat count in *C9ORF72* than previously identified with southern blotting. This might be explained through the observation of a reduced read quality of the expanded allele potentially due to the low complexity of the high GC‐rich motif and the length of the expansion. Hence, the exact repeat length determination is probably not possible using only IGV. Therefore, appropriate bioinformatics tools need to be taken into consideration that likely require more coverage. That could be easily achieved by a higher sequencing throughput. Besides this, the results of the southern blotting also have to be interpreted with caution as it is only an indirect determination of repeat expansion lengths.

Additionally, we determined the STR expansions in the genes *FXN* and *C9ORF72* in extensive length distributions that probably reflects the somatic mosaicism of large repeat expansions due to the instability of the repeat. This has already been described for the investigated genes [26, 27] and is also in accordance with the observation by Erdmann et al. [8] for large expansions in the gene *FMR1* using nanopore sequencing.

In conclusion, we observed read length distributions suitable for STR expansion detection with nanopore sequencing as long as the DNA samples had a DIN value higher than 9.0. Even though STRs can be up to 40 kb of total repeat size, for example, in the gene *CNBP*, it is for diagnostic purposes not necessary to determine the exact of those large expansions. It is rather of interest if there is an expansion exceeding the described pathological repeat size, which is at maximum an order of magnitude lower (e.g., STRs in *TNRC6A* and *YEATS2* are pathologically expanded at more than 1100 and 1000 quintuplet repeat units). Hence, this might be well within the N50 values obtained in our study. Furthermore, we established the DIN value to be a good predictor for read length distribution for nanopore sequencing and therefore useful for sample selection and evaluation of the DNA extraction method. The required DIN values can be achieved with the automated magnetic bead–based DNA extraction method assuming that the EDTA blood samples have been short‐term stored, cooled, or frozen. In short, it is worth evaluating the established DNA extraction methods in diagnostic laboratories in order to omit dedicated elaborate high‐molecular‐weight DNA extraction methods.

## Conflicts of Interest

The authors declare no conflicts of interest.

## Supporting information


Appendix S1

Appendix S2

Appendix S3

Appendix S4

Appendix S5

Appendix S6

Tables S1–S6

## Data Availability

The data that support the findings of this study are available in the [Link], [Link], [Link], [Link], [Link], [Link], [Link] of this article.
