# Supplementary material for: Evaluation of Automated Magnetic Bead–Based DNA Extraction for Detection of Short Tandem Repeat Expansions With Nanopore Sequencing
Source: J Clin Lab Anal. 2024 Mar 20;38(6):e25029. doi: 10.1002/jcla.25029 (PMC10997813; doi:10.1002/jcla.25029)
Supplement: Supplementary file 1 — Appendix S1 [file JCLA-38-e25029-s005.pdf]

Chromosome	start position	end position	Gene
chr1	1385798	1485818	VWA1
chr1	57317043	57417118	DAB1
chr1	149340802	149440841	NOTCH2NLC
chr10	79776383	79876404	NUTM2B-AS1
chr11	119156290	119256323	CBL
chr12	6886716	6986773	ATN1
chr12	50455001	50555022	DIP2B
chr12	111548949	111649018	ATXN2
chr12	123483721	123583755	RILPL1
chr13	70089353	70189428	ATXN8OS
chr13	99935448	100035493	ZIC2
chr14	23271472	23371510	PABPN1
chr14	92021009	92121042	ATXN3
chr15	22736677	22836701	NIPA1
chr16	2038710	2185898	PKD1
chr16	17420907	17520922	XYLT1
chr16	24563439	24663529	TNRC6A
chr16	66440398	66540453	BEAN1
chr16	87554287	87654329	JPH3
chr17	80097059	80197139	EIF4A3
chr18	55536155	55636227	TCF4
chr19	13157858	13257897	CACNA1A
chr19	14446041	14546074	GIPC1
chr19	18736034	18836049	COMP
chr19	45720204	45820264	DMPK
chr2	96147066	96247121	STARD7
chr2	176043058	176143103	HOXD13
chr2	190830872	190930920	GLS
chr20	2602733	2702775	NOP56
chr20	4649370	4749493	PRNP
chr21	43726443	43826479	CTSB
chr22	19716762	19816807	TBX1
chr22	28637743	28792422	CHEK2
chr22	45745354	45845424	ATXN10
chr3	63862684	63962726	ATXN7
chr3	129122576	129222732	CNBP
chr3	138896020	138996062	FOXL2
chr3	183662187	183762222	YEATS2
chr4	3024876	3124966	HTT
chr4	39298424	39398479	RFC1
chr4	41695972	41796032	PHOX2B
chr4	159292526	159392616	RAPGEF2
chr5	10306346	10406411	MARCHF6
chr5	146828727	146928757	PPP2R2B
chr6	16277633	16377723	ATXN1
chr6	45372750	45472792	RUNX2
chr6	170511906	170612017	TBP
chr7	5920925	6059049	PMS2
chr7	27149678	27249966	HOXA13

chr8	104538970	104638997	LRP12
chr8	118316815	118416880	SAMD12
chr9	27523528	27623546	C9ORF72
chr9	68987261	69087304	FXN
chr9	130631606	130731639	PRDM12
chrX	24963529	25063697	ARX
chrX	31234557	31334605	DMD
chrX	38235540	38337245	RPGR
chrX	67495316	67595385	AR
chrX	71374239	71475356	TAF1
chrX	137516826	137616856	ZIC3
chrX	140454316	140554361	SOX3
chrX	147862050	147962110	FMR1
chrX	148450631	148550691	AFF2
chrX	154497620	154615033	G6PD
