# Supplementary material for: Evaluation of Automated Magnetic Bead–Based DNA Extraction for Detection of Short Tandem Repeat Expansions With Nanopore Sequencing
Source: J Clin Lab Anal. 2024 Mar 20;38(6):e25029. doi: 10.1002/jcla.25029 (PMC10997813; doi:10.1002/jcla.25029)

B1: Individual 1_Iso 1_fresh blood

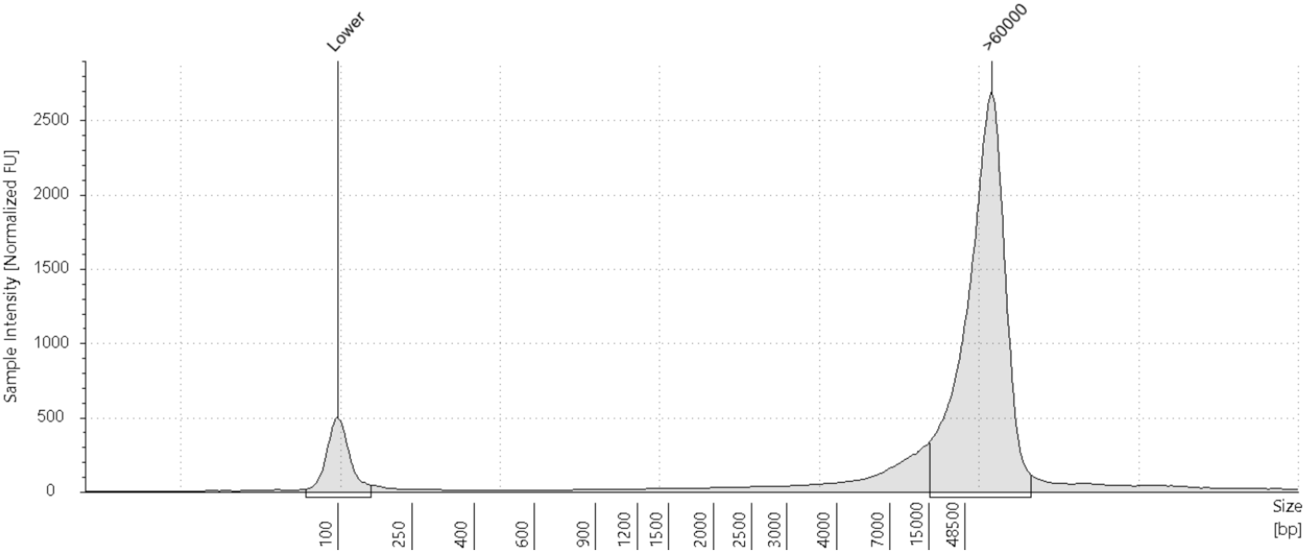

C1: [redacted] Individual 1_Iso 2_fresh blood

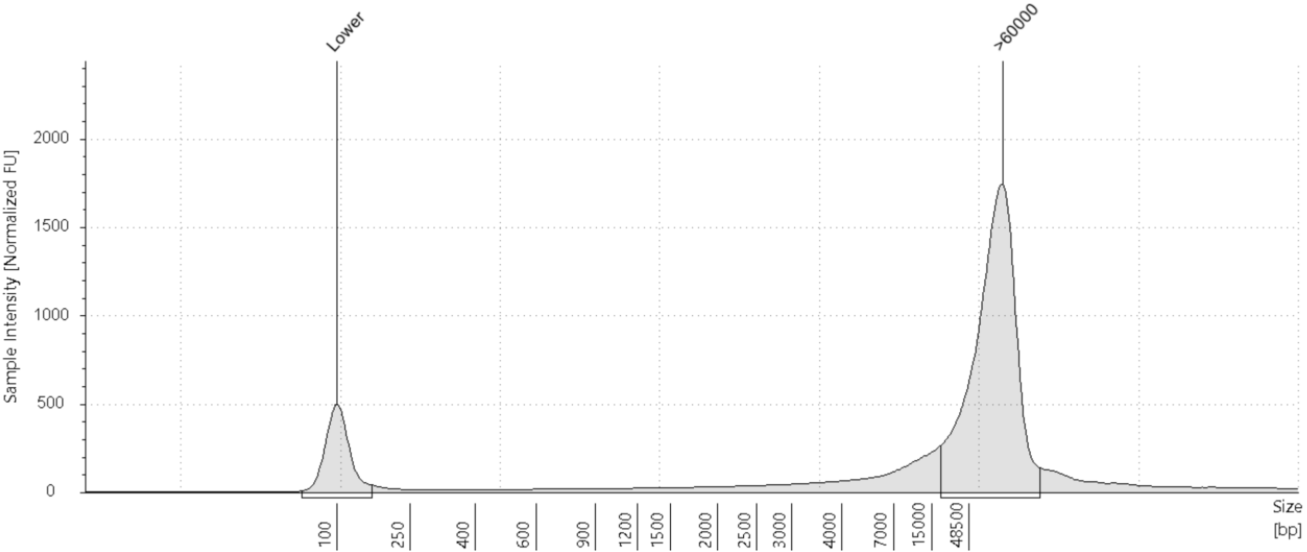

C1: [redacted] Individual 1_Iso 3_fresh blood

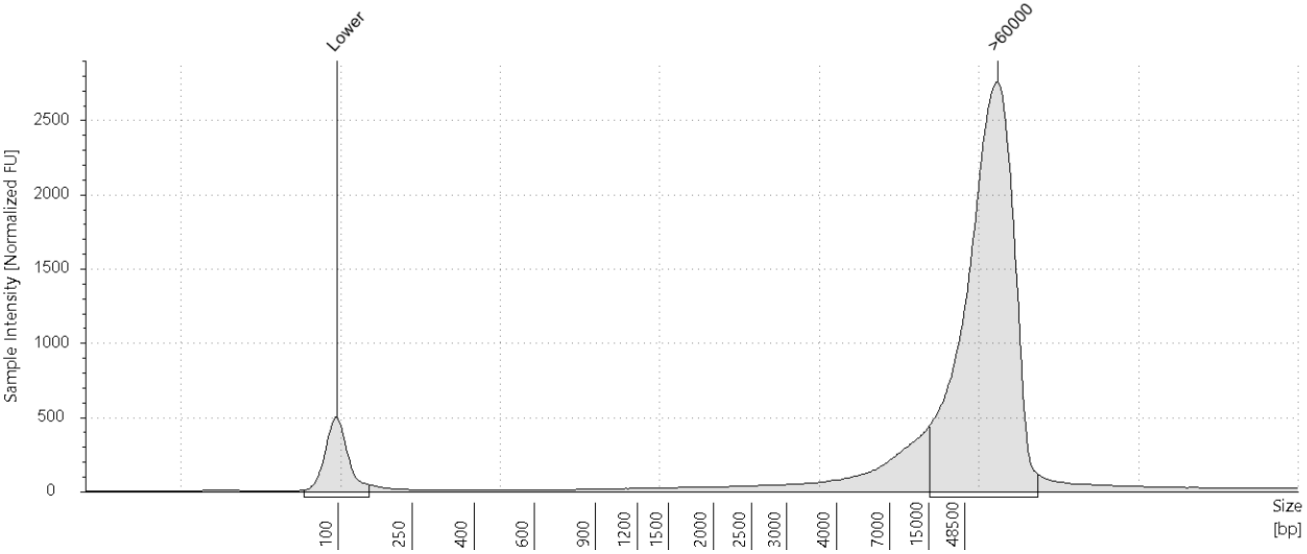

E1: [redacted] Individual 1_Iso 4_fresh blood

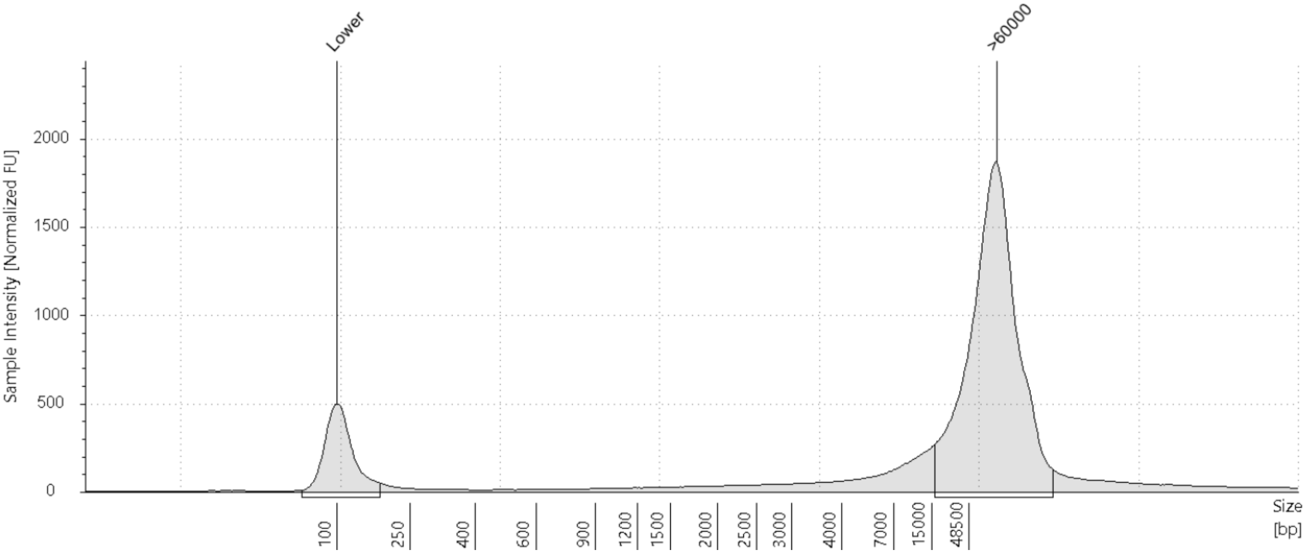

D1: [redacted] Individual 2_Iso 1_fresh blood

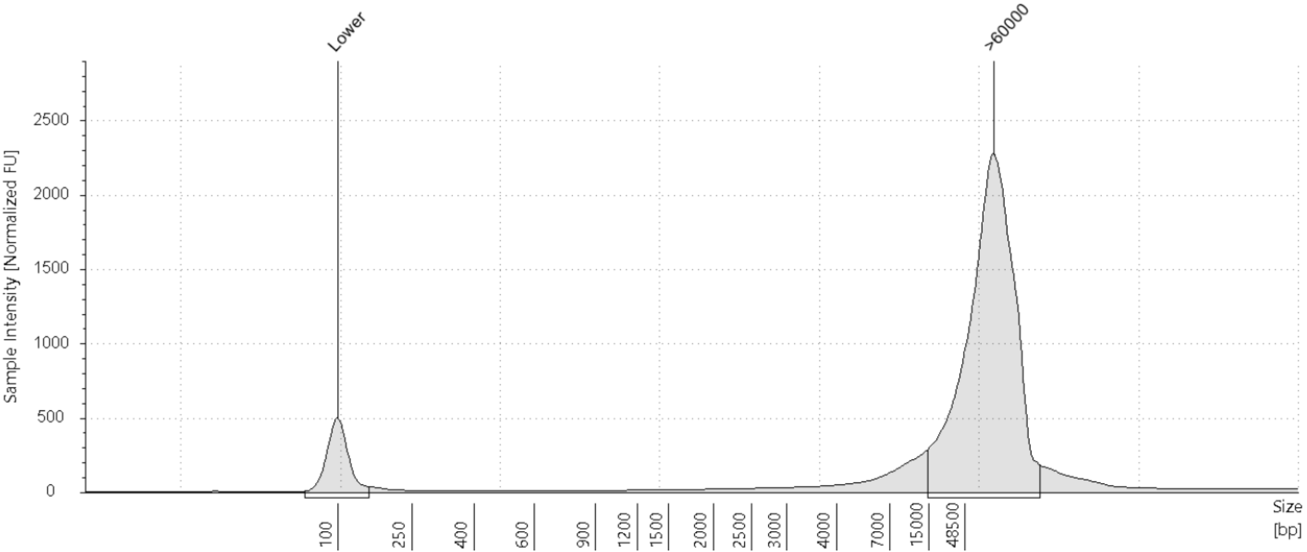

G1: [redacted] Individual 2_Iso 2_fresh blood

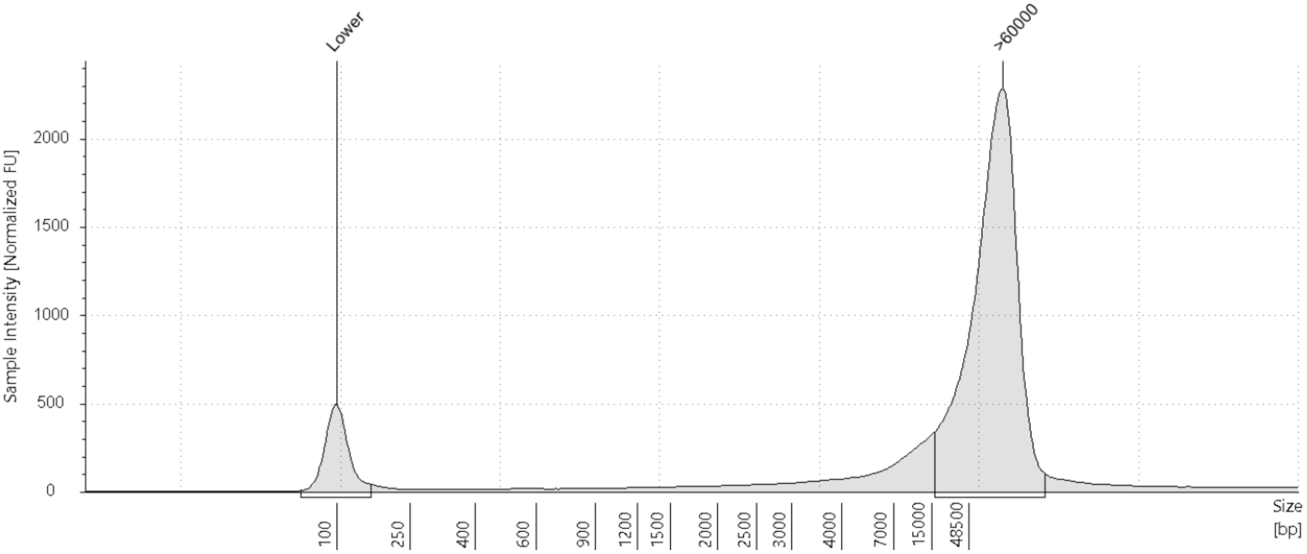

E1: [redacted] Individual 2_Iso 3_fresh blood

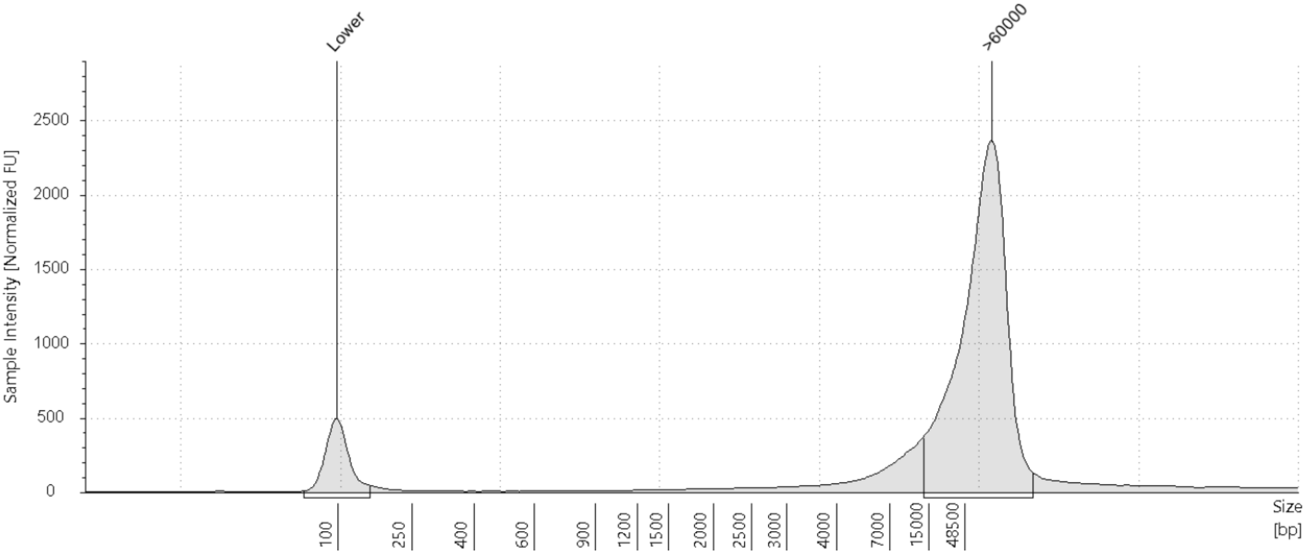

A2: [redacted] Individual 2_Iso 4_fresh blood

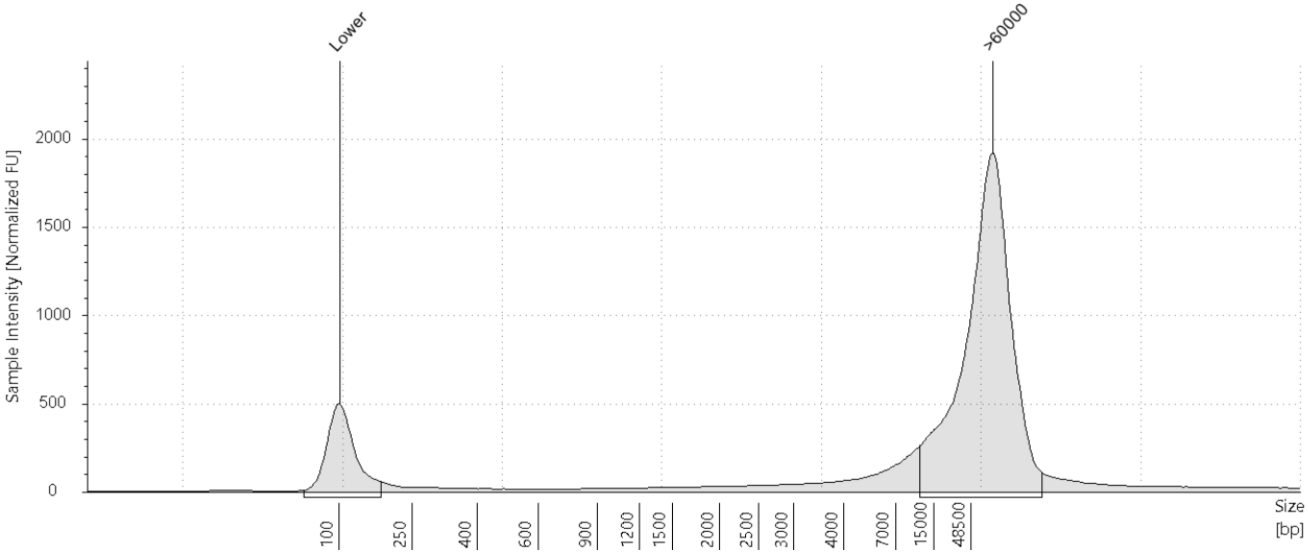

F1: [redacted] Individual 3_Iso 1_fresh blood

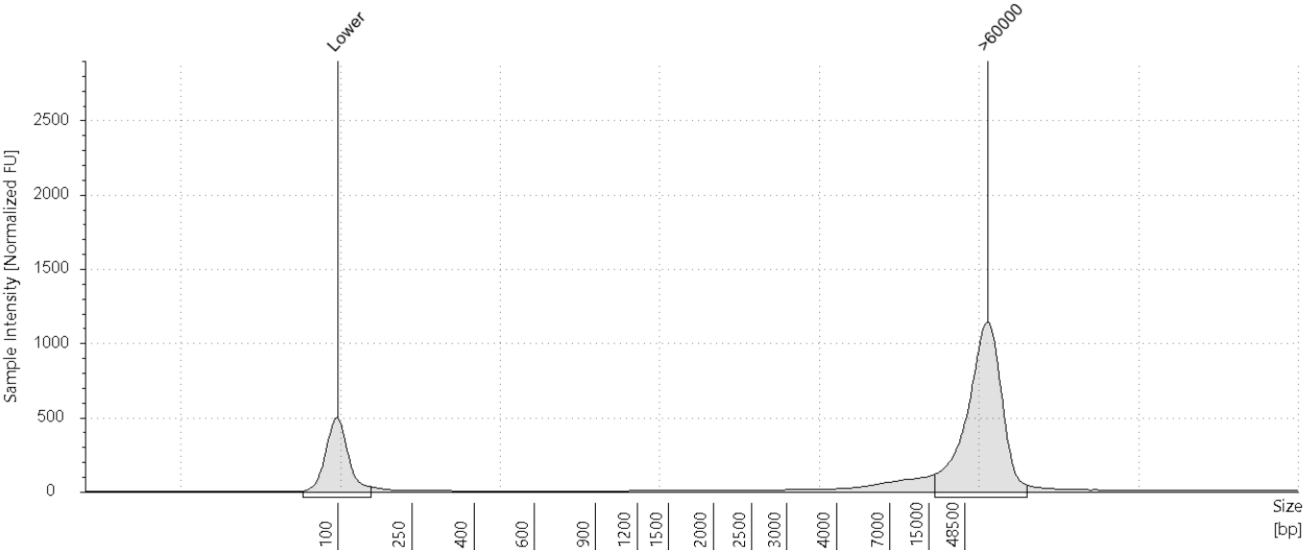

C2: Individual 3_Iso 2_fresh blood

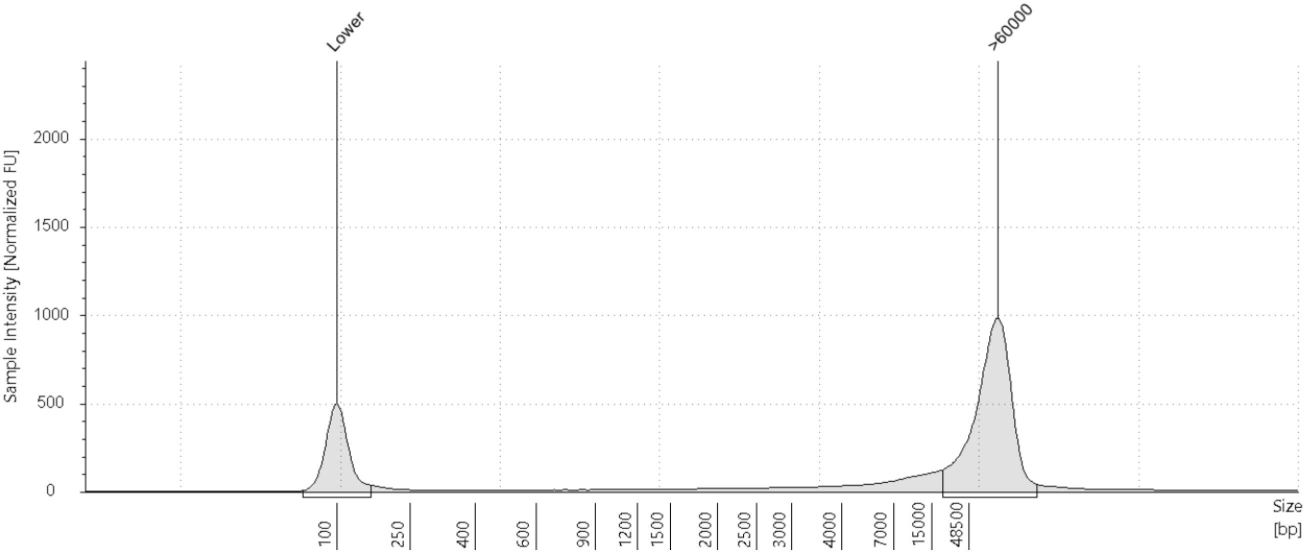

D2: [redacted] Individual 3_Iso 3_fresh blood

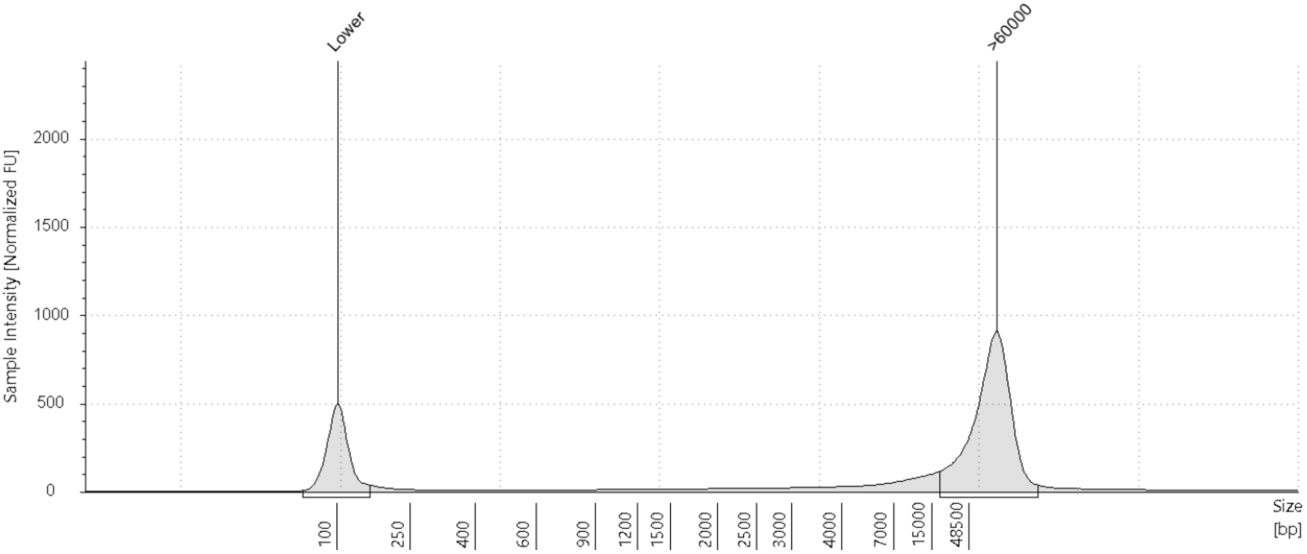

G1: [redacted] Individual 3_Iso 4_fresh blood

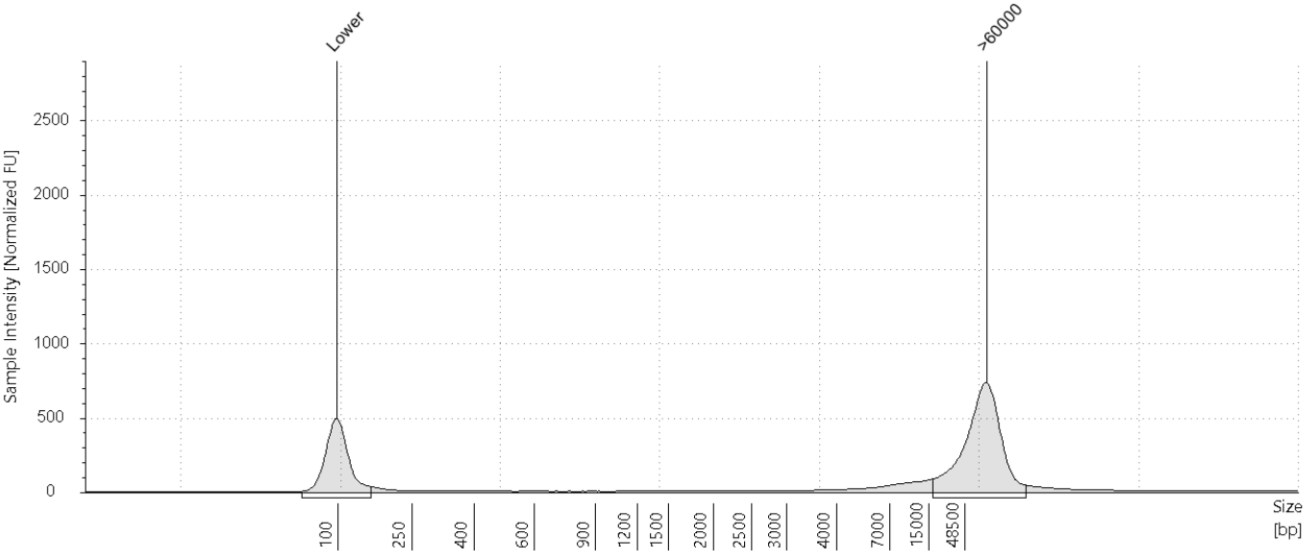

F2: [redacted] Individual 4_Iso 1_fresh blood

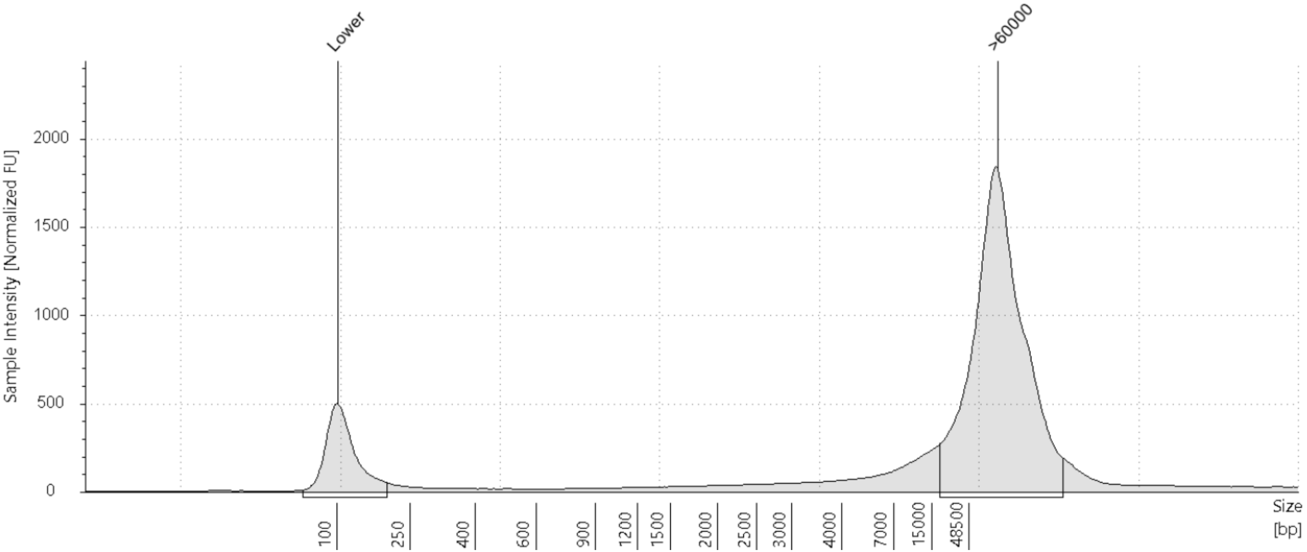

G2: [redacted] Individual 4_Iso 2_fresh blood

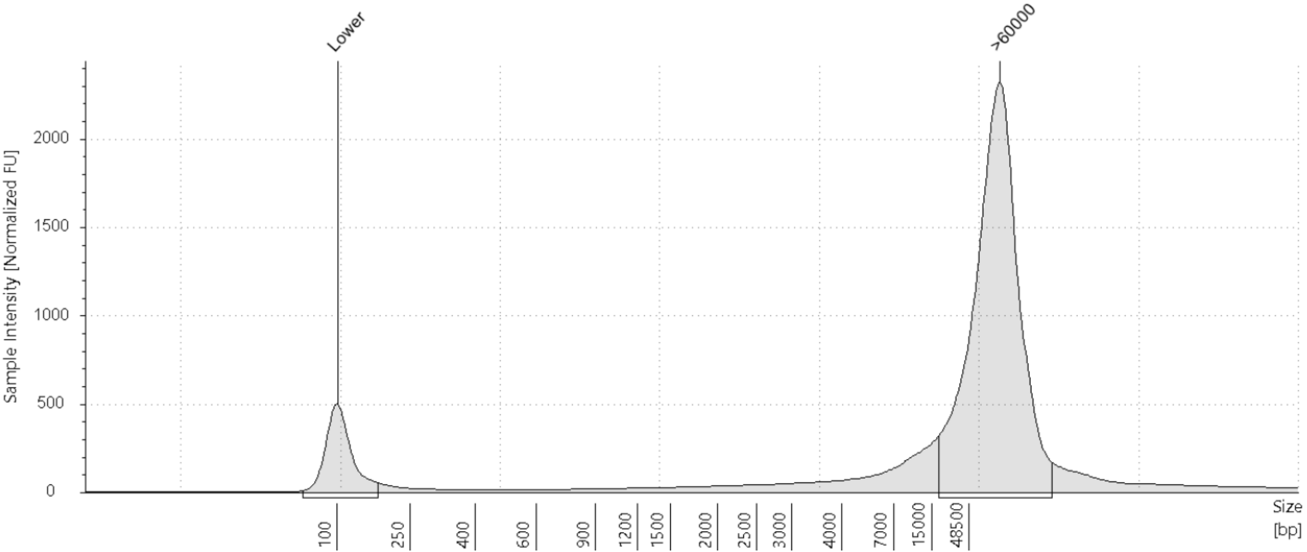

H2: [redacted] Individual 4_Iso 3_fresh blood

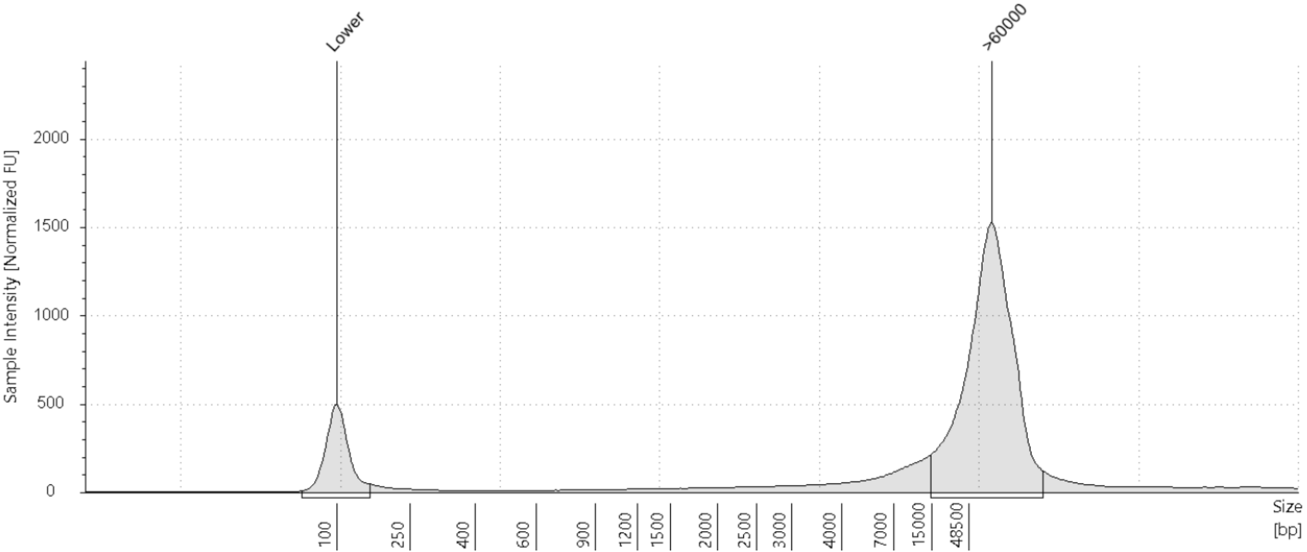

H1: [redacted] Individual 4_Iso 4_fresh blood

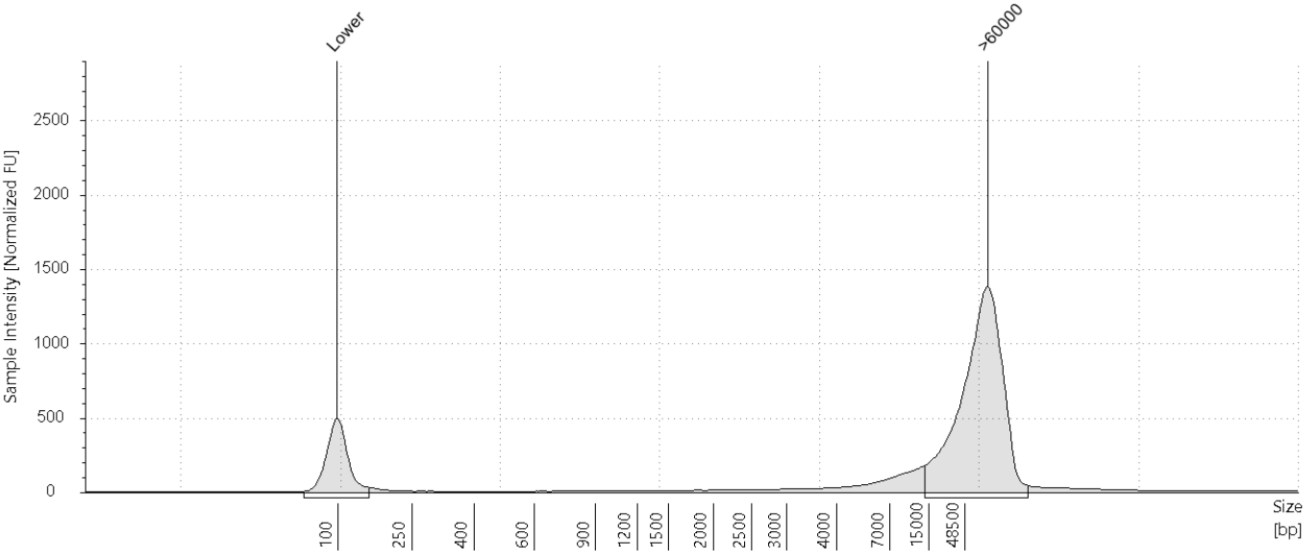

B1: [redacted] Individual 1_Iso 1_room temperature

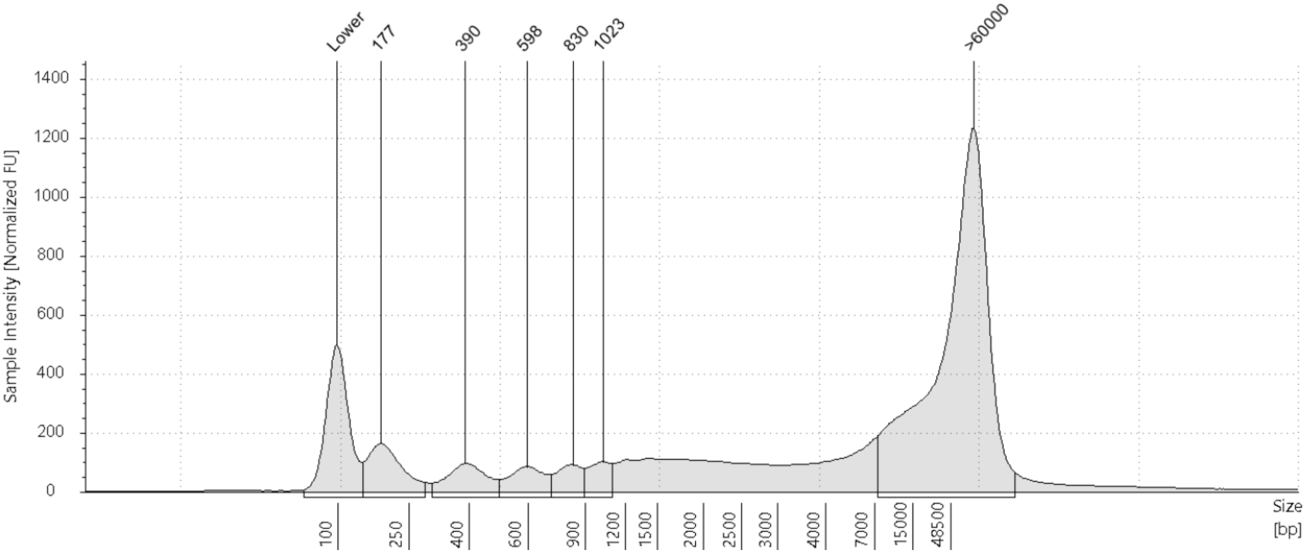

C1: [redacted] Individual 1_Iso 2_room temperature

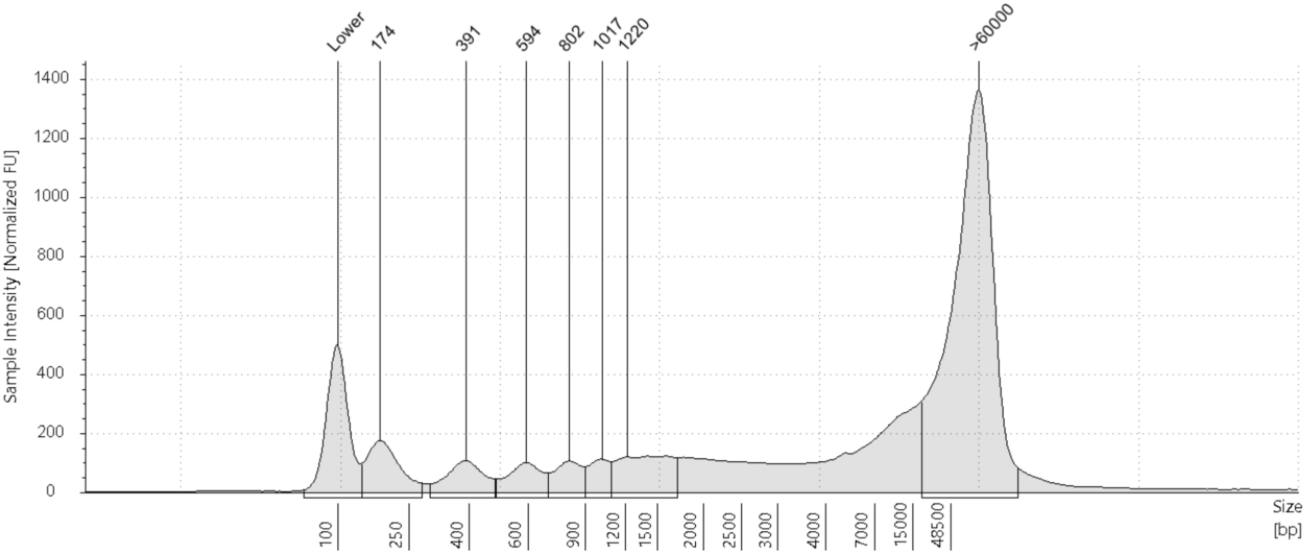

D1: [redacted] Individual 1_Iso 3_room temperature

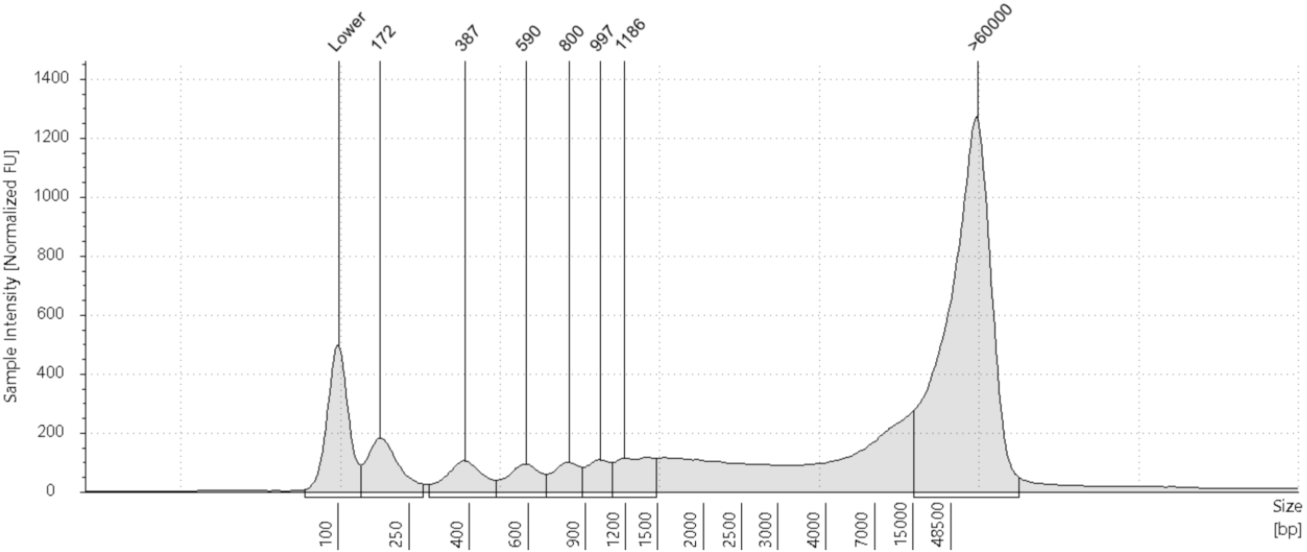

E1: [redacted] Individual 1_Iso 4_room temperature

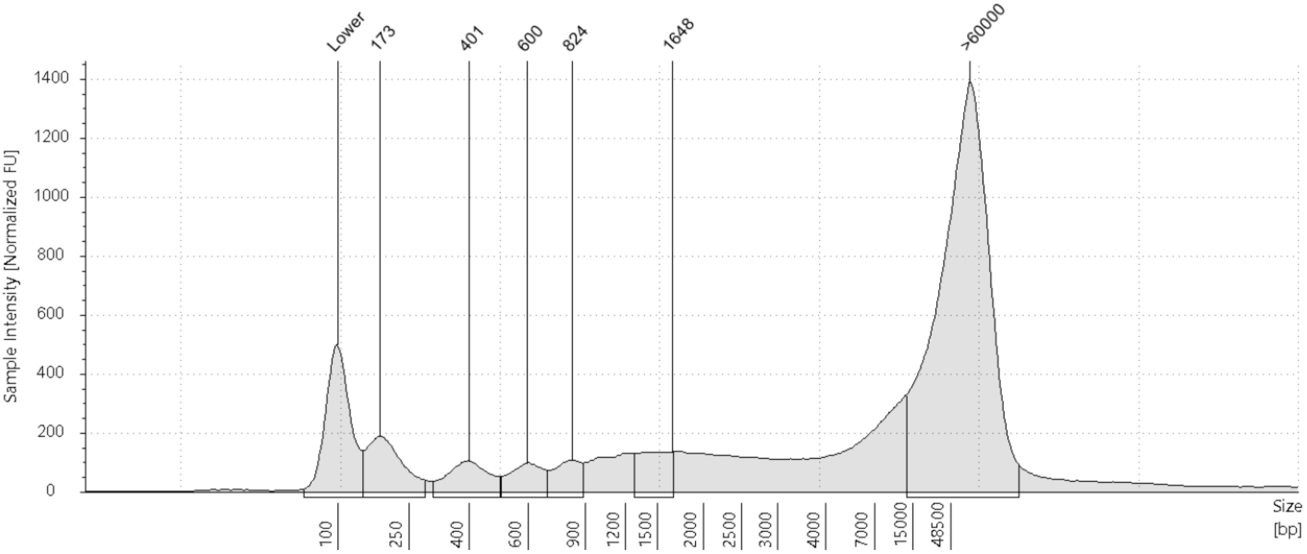

F1: [redacted] Individual 2_Iso 1_room temperature

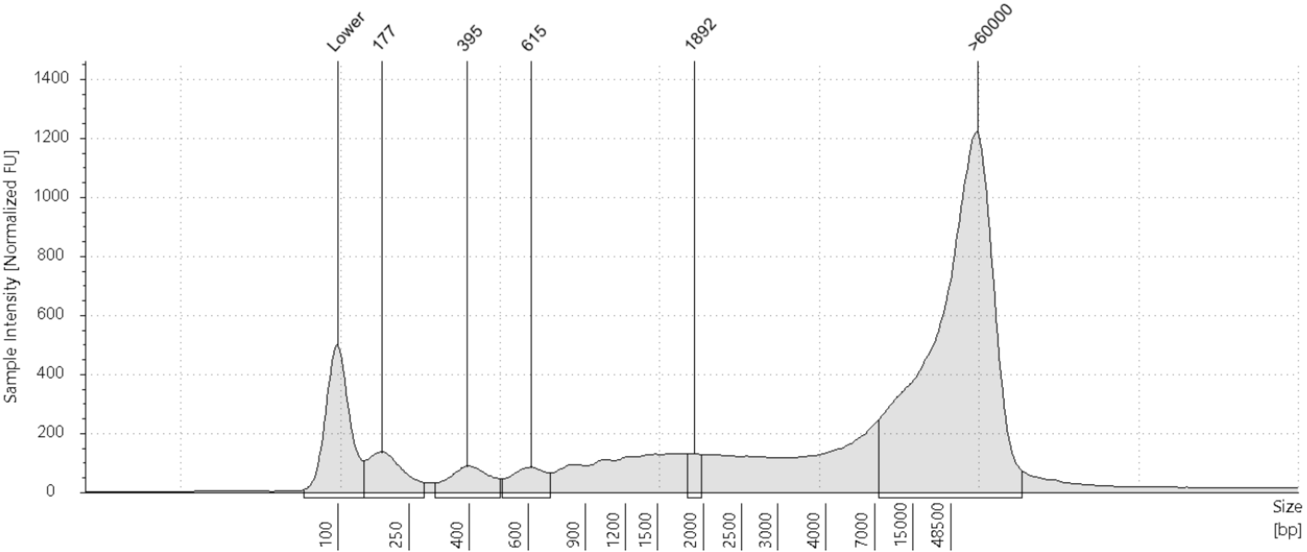

G1: [redacted] Individual 2_Iso 2_room temperature

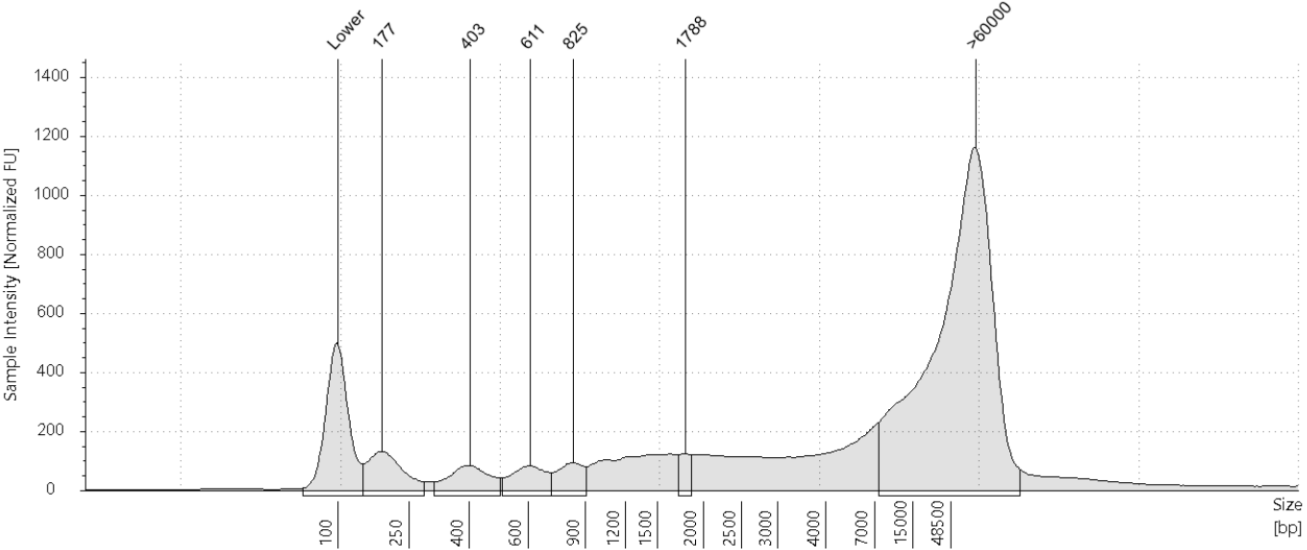

H1: [redacted] Individual 2_Iso 3_room temperature

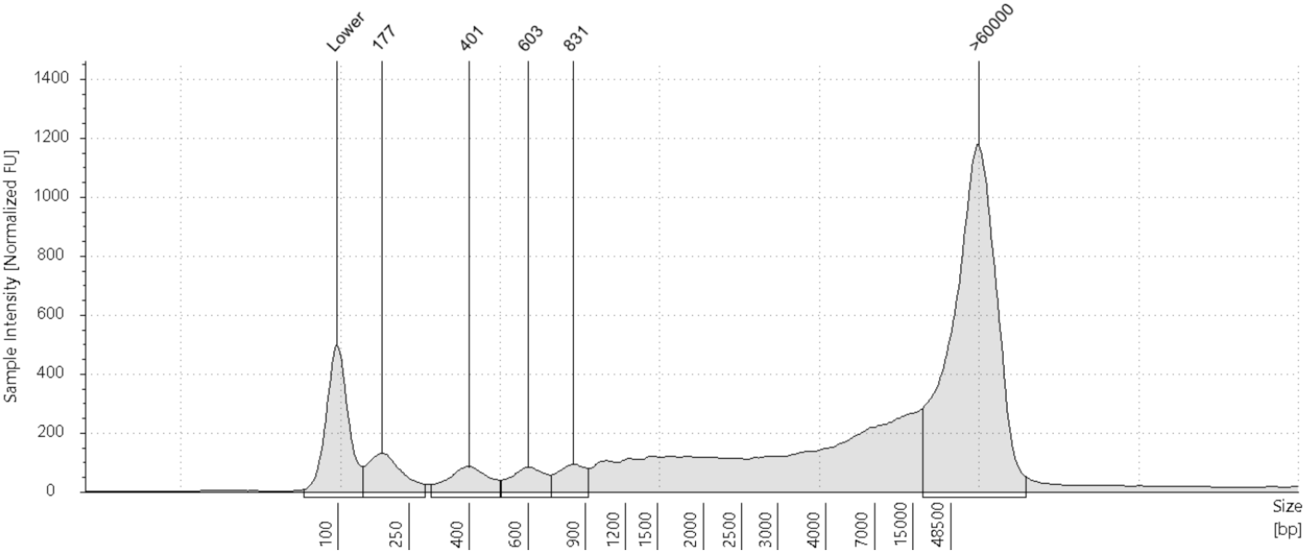

A2: [redacted] Individual 2_Iso 4_room temperature

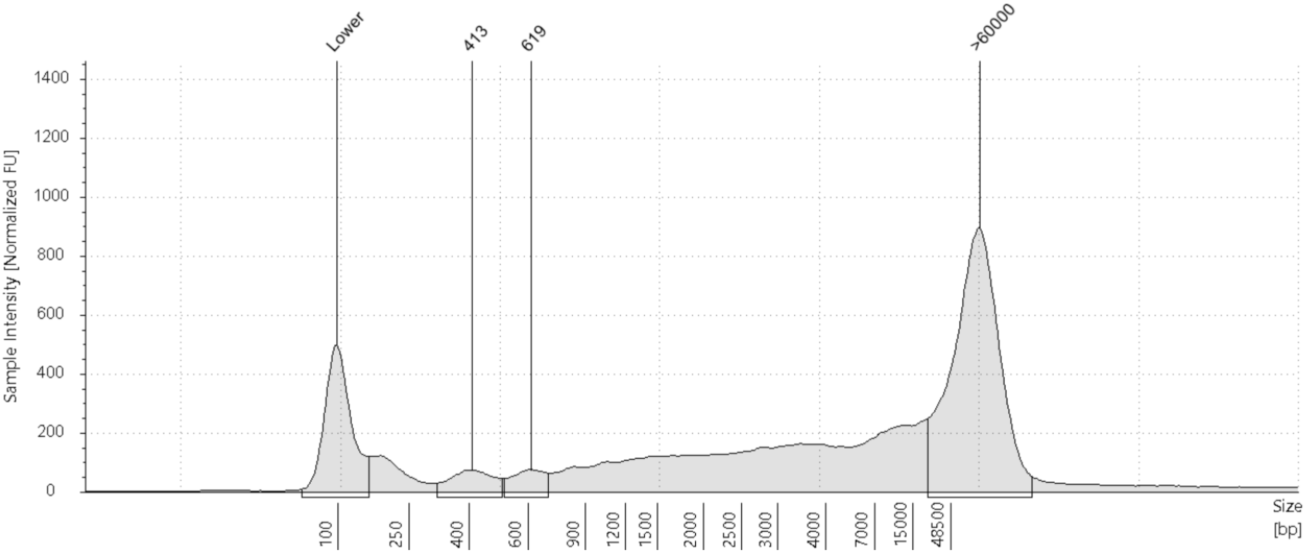

B2: [redacted] Individual 3_Iso 1_room temperature

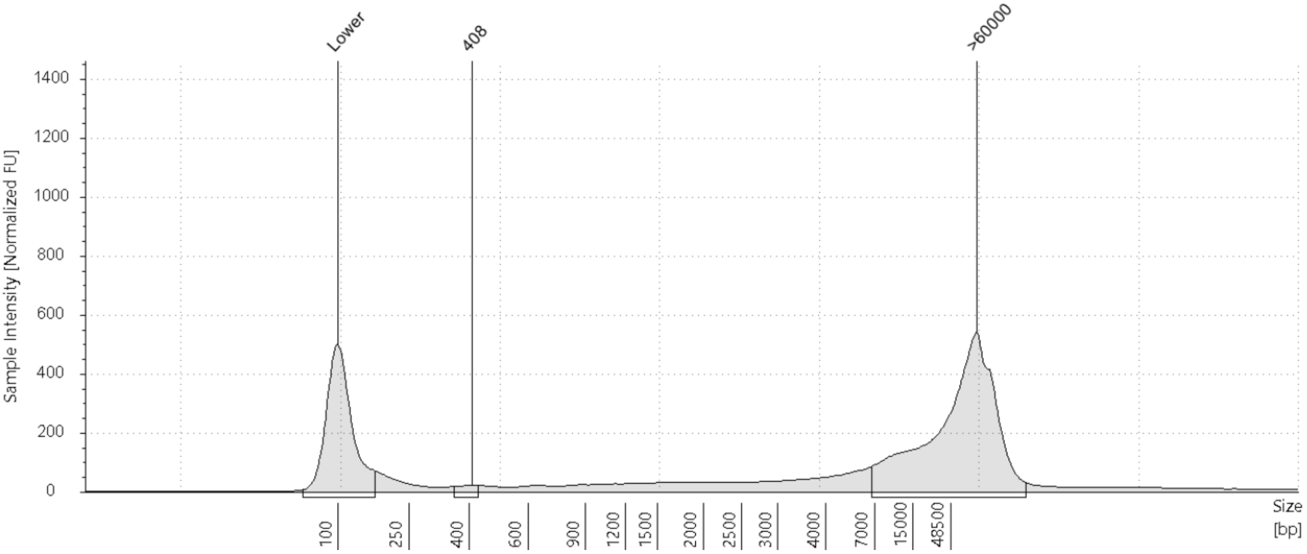

C2: [redacted] Individual 3_Iso 2_room temperature

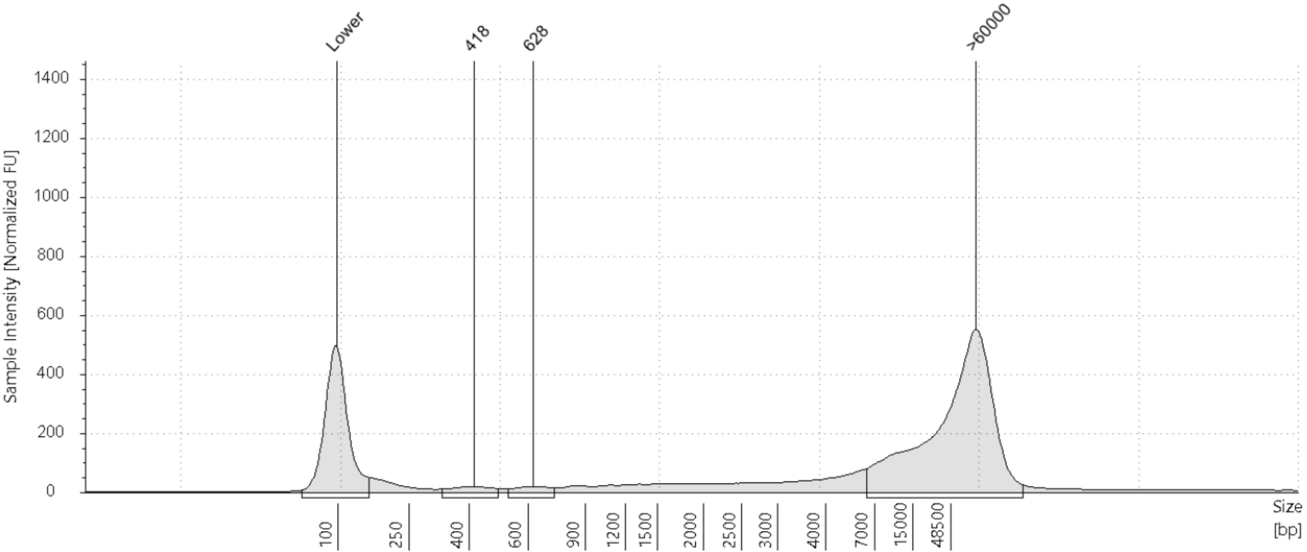

D2: [redacted] Individual 3_Iso 3_room temperature

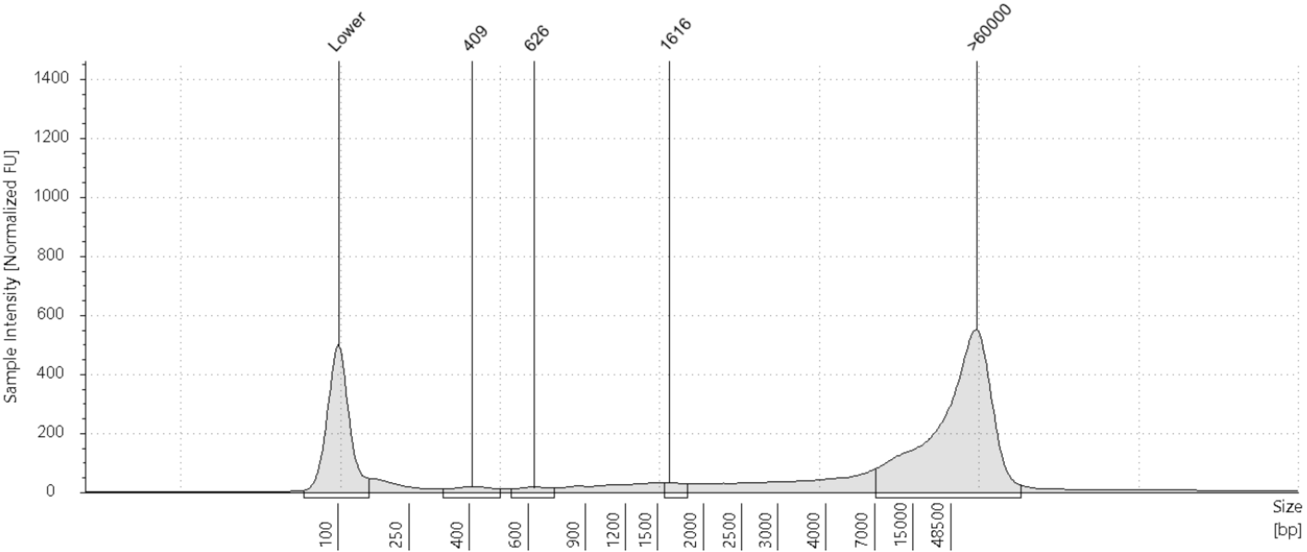

E2: [redacted] Individual 3_Iso 4_room temperature

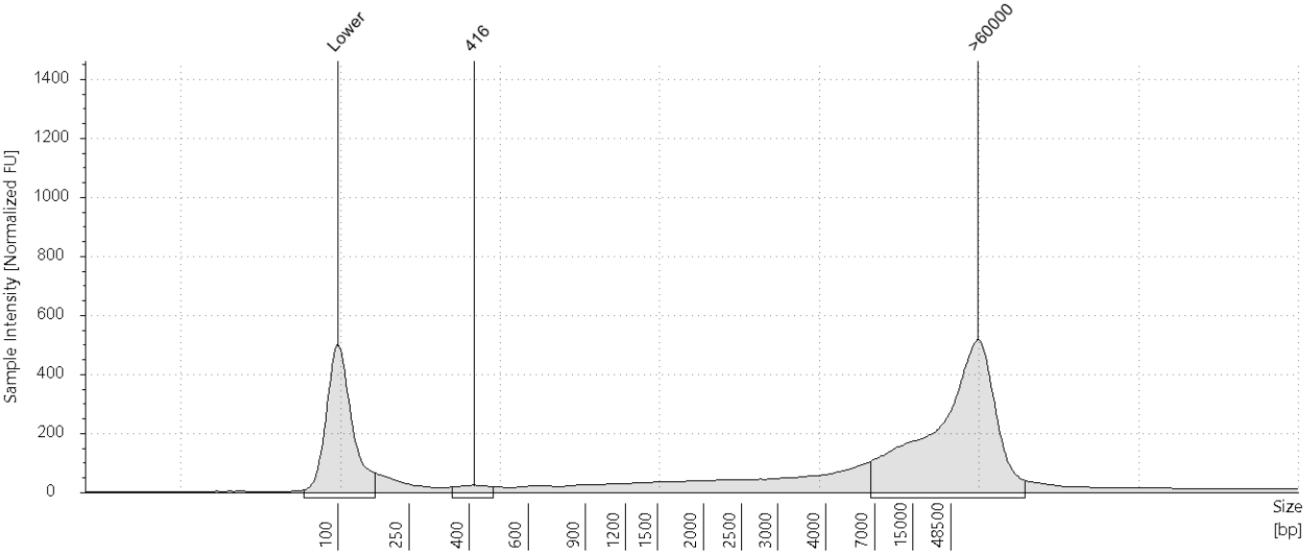

F2: [redacted] Individual 4_Iso 1_room temperature

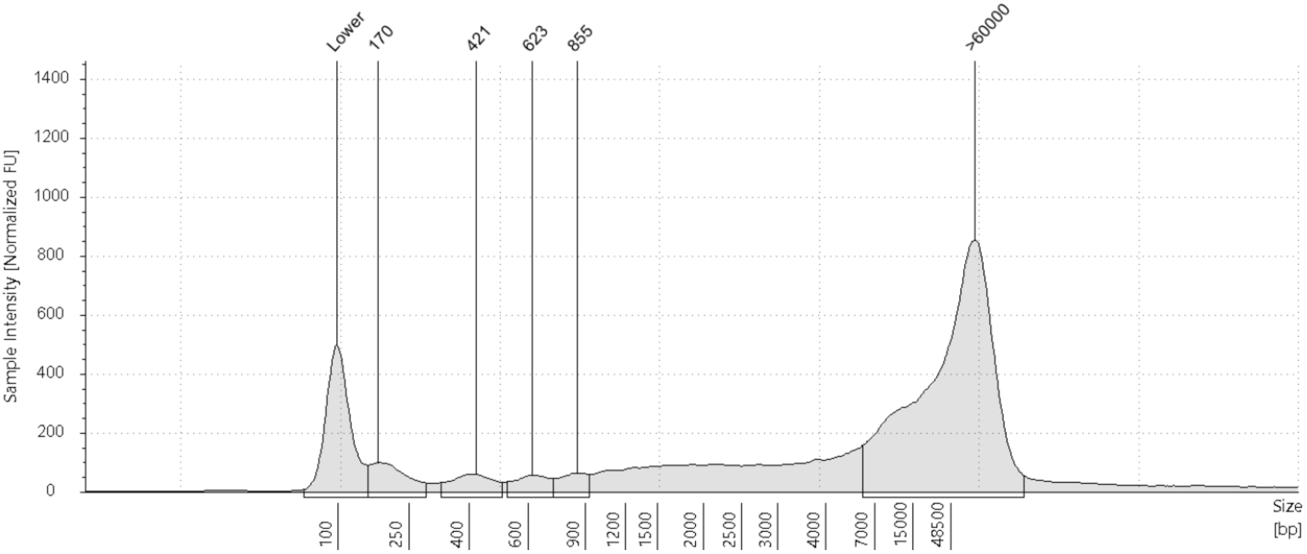

G2: [redacted] Individual 4_Iso 2_room temperature

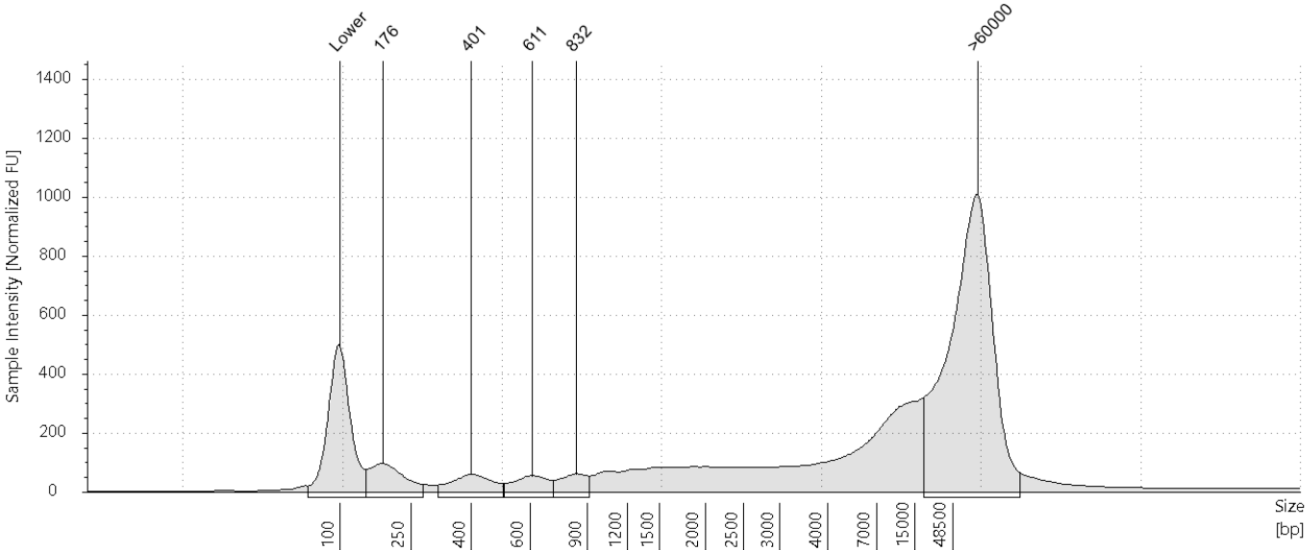

H2: [redacted] Individual 4_Iso 3_room temperature

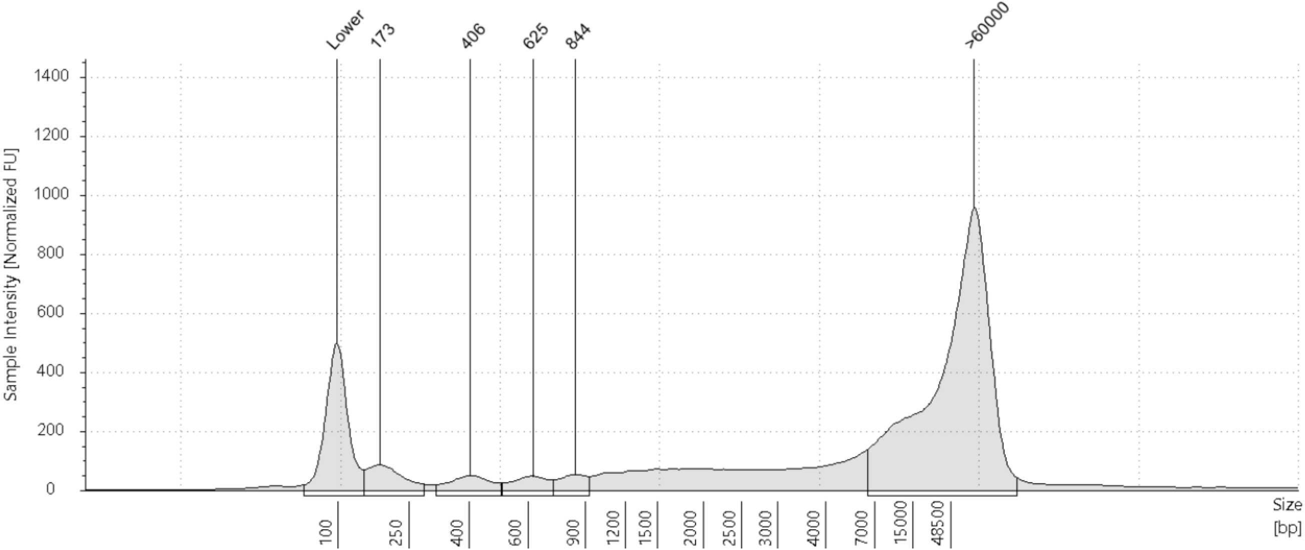

B1: [redacted] Individual 4_Iso 4_room temperature

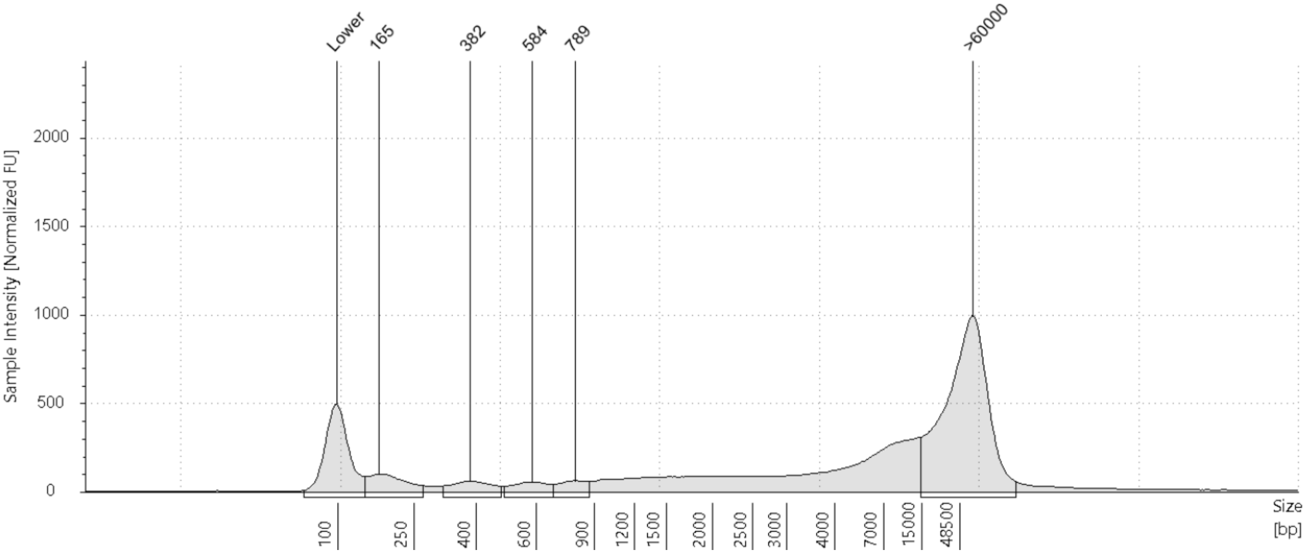

C1: [redacted] Individual 1_Iso 1_4°C

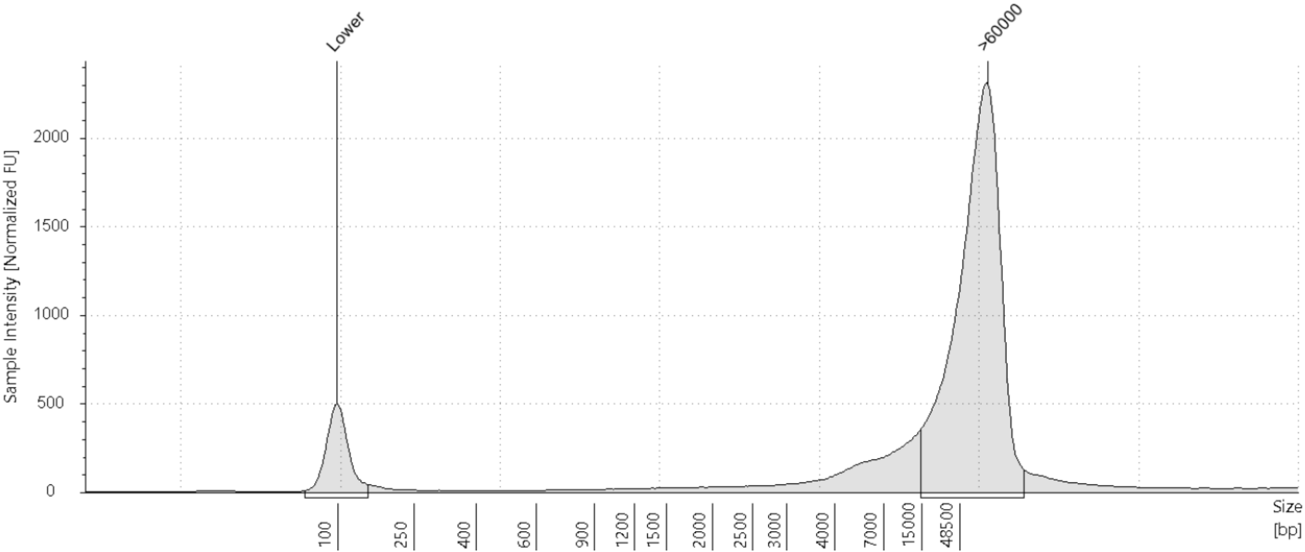

D1: [redacted] Individual 1_Iso 2_4°C

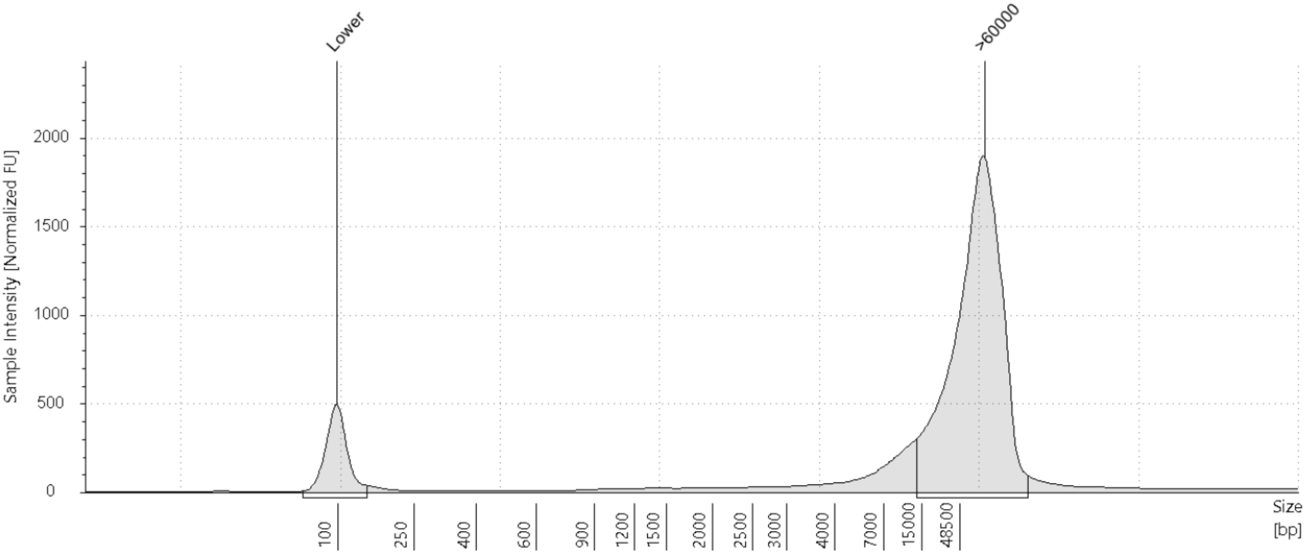

E1: [redacted] Individual 1_Iso 3_4°C

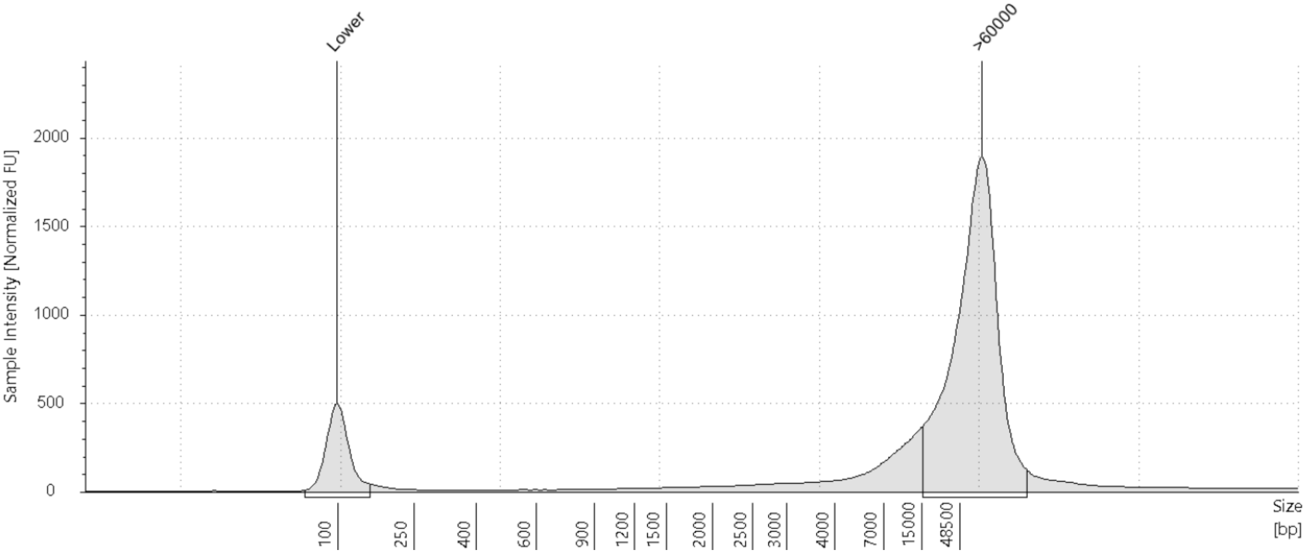

F1: [redacted] Individual 1_Iso 4_4°C

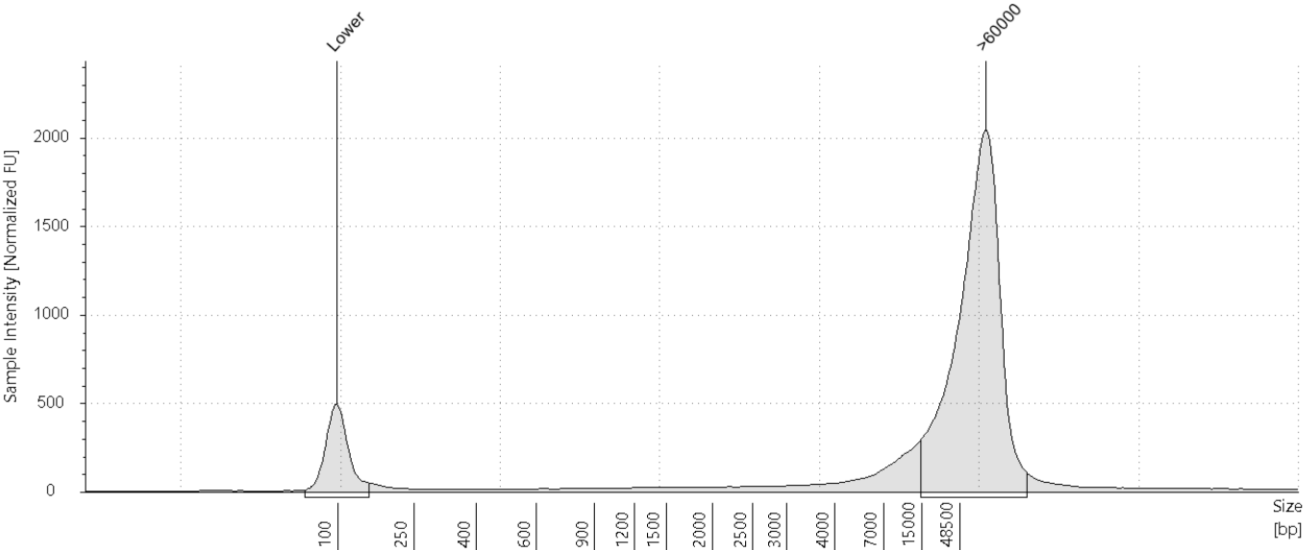

G1: [redacted] Individual 2_Iso 1_4°C

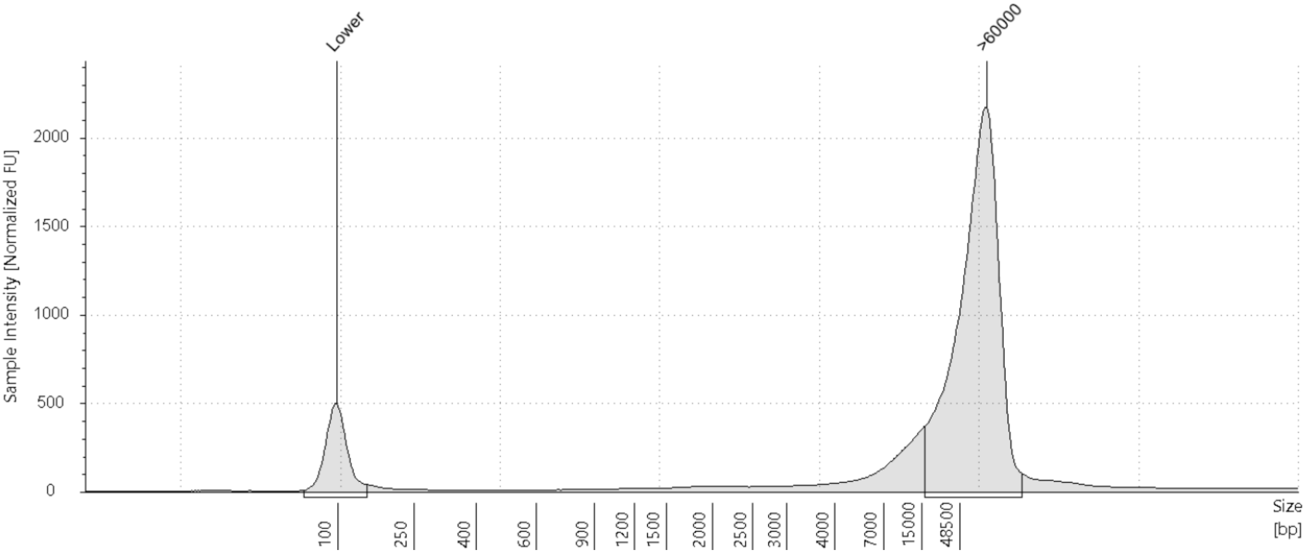

H1: [redacted] Individual 2_Iso 2_4°C

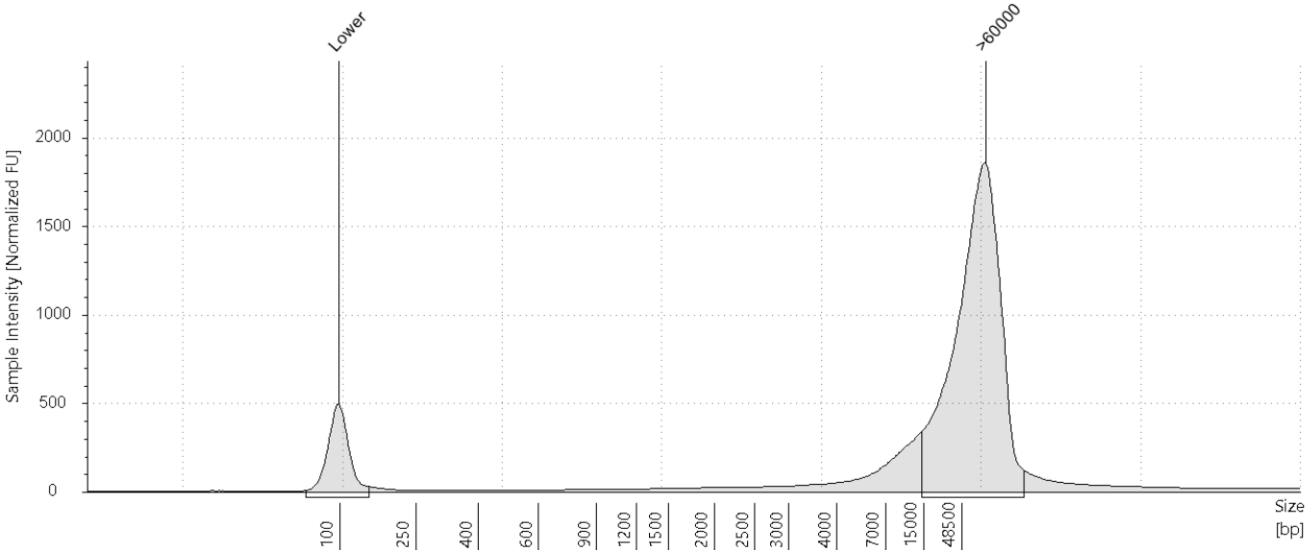

A2: [redacted] Individual 2_Iso 3_4°C

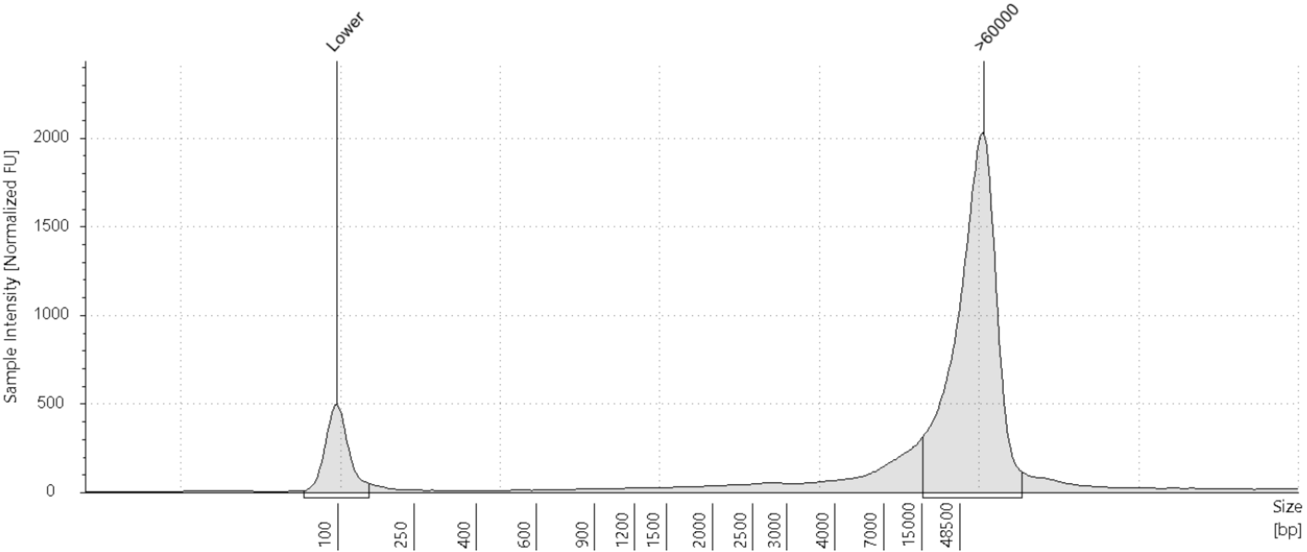

B2: [redacted] Individual 2_Iso 4_4°C

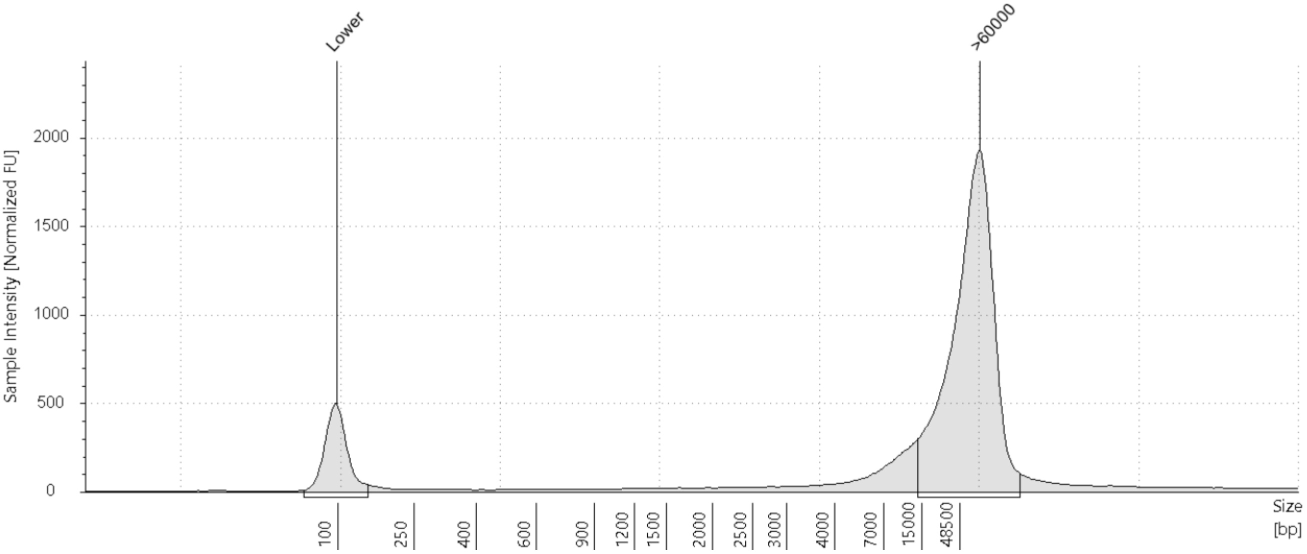

C2: [redacted] Individual 3_Iso 1_4°C

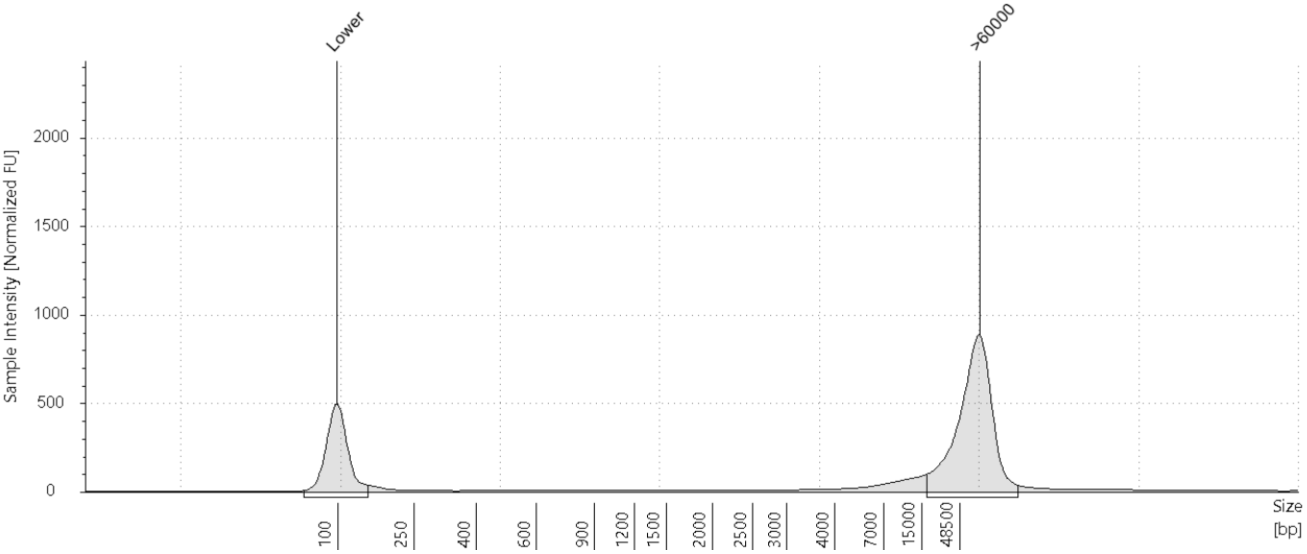

D2: [redacted] Individual 3_Iso 2_4°C

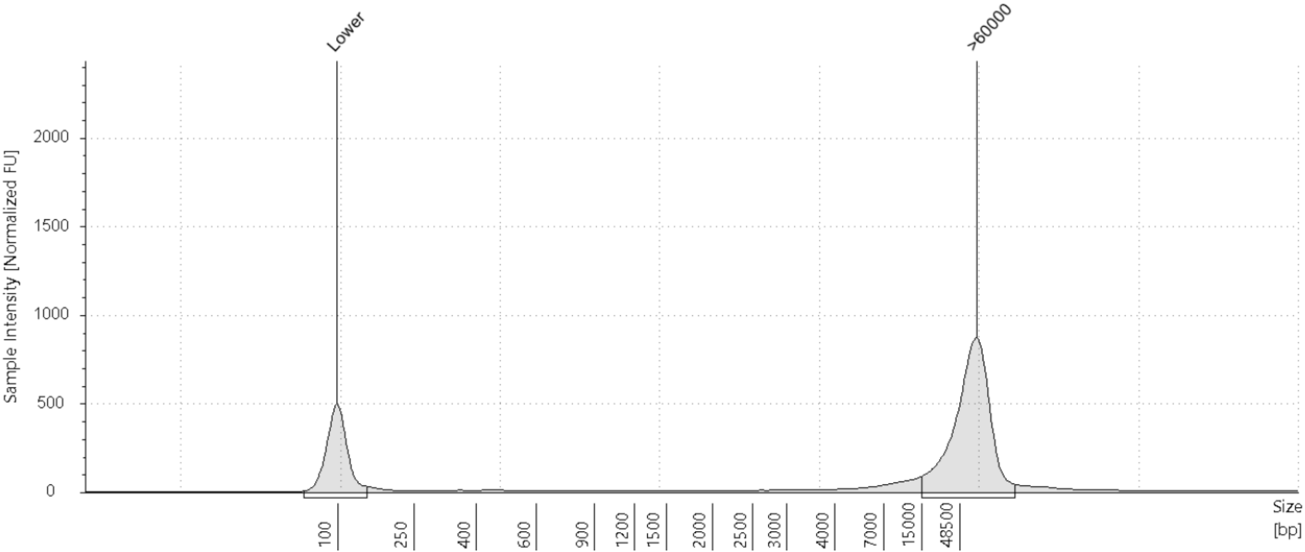

E2: [redacted] Individual 3_Iso 3_4°C

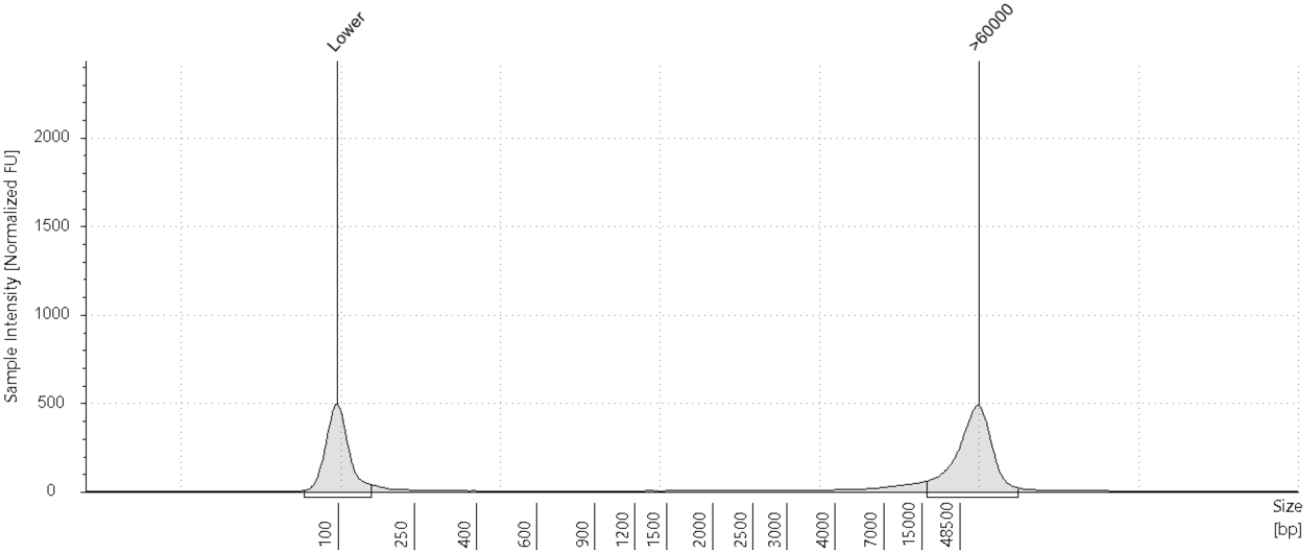

F2: [redacted] Individual 3_Iso 4_4°C

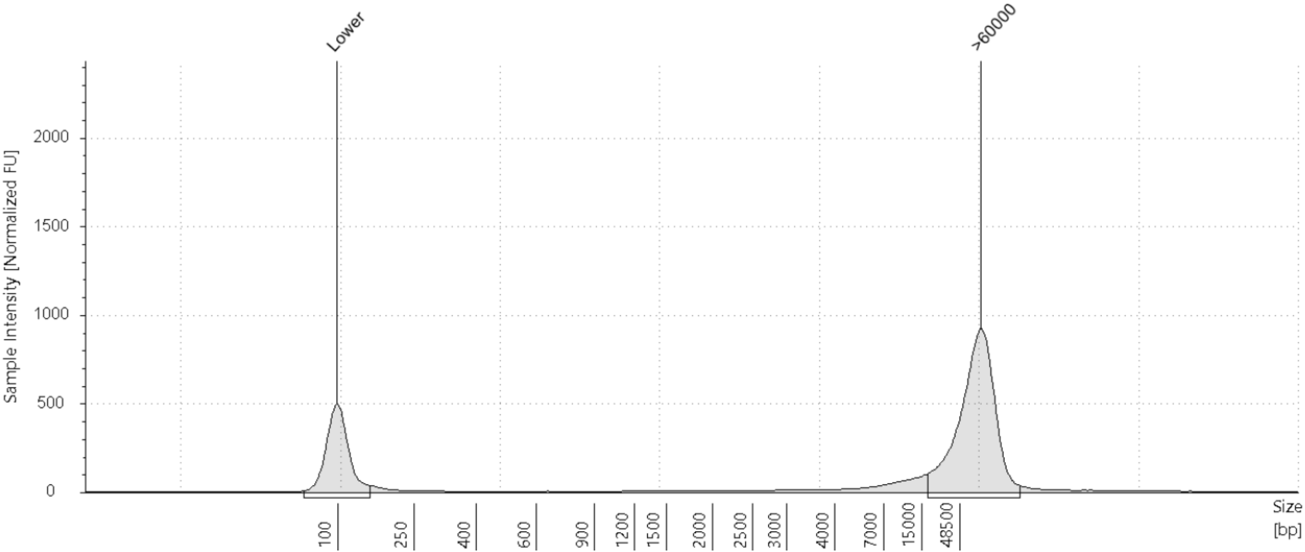

G2: Individual 4_Iso 1_4°C

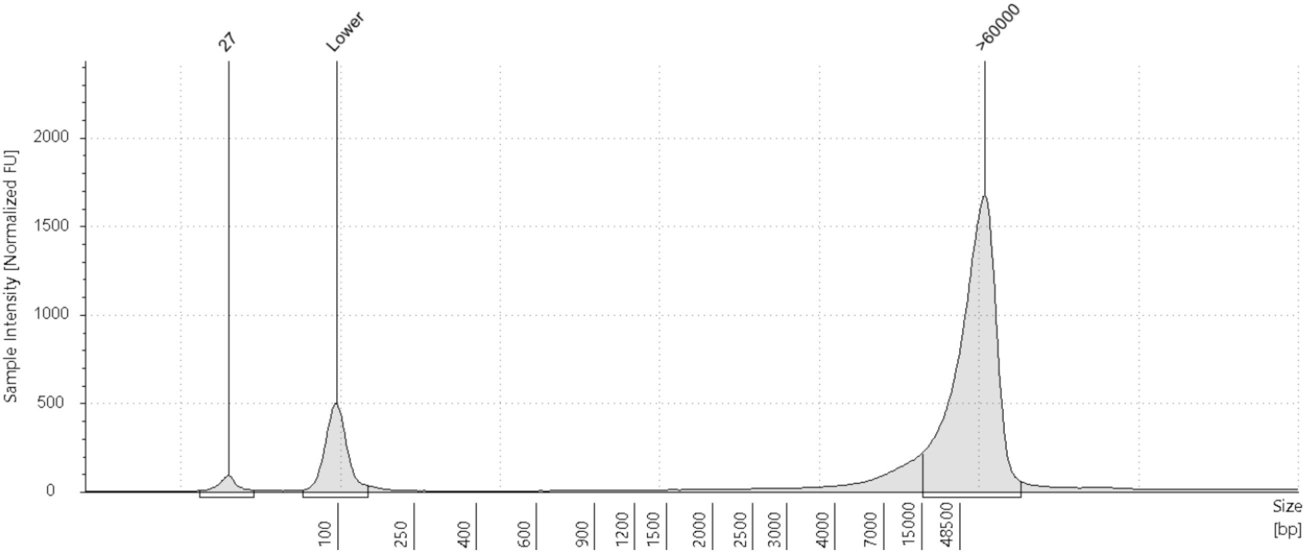

H2: [redacted] Individual 4_Iso 2_4°C

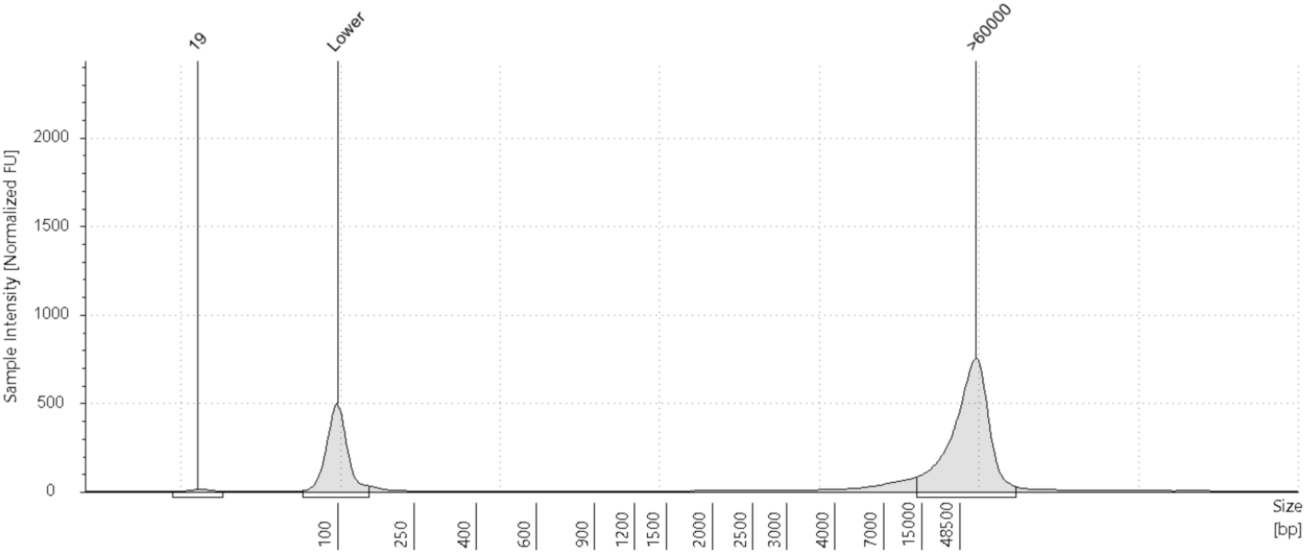

B1: [redacted] Individual 4_Iso 3_4°C

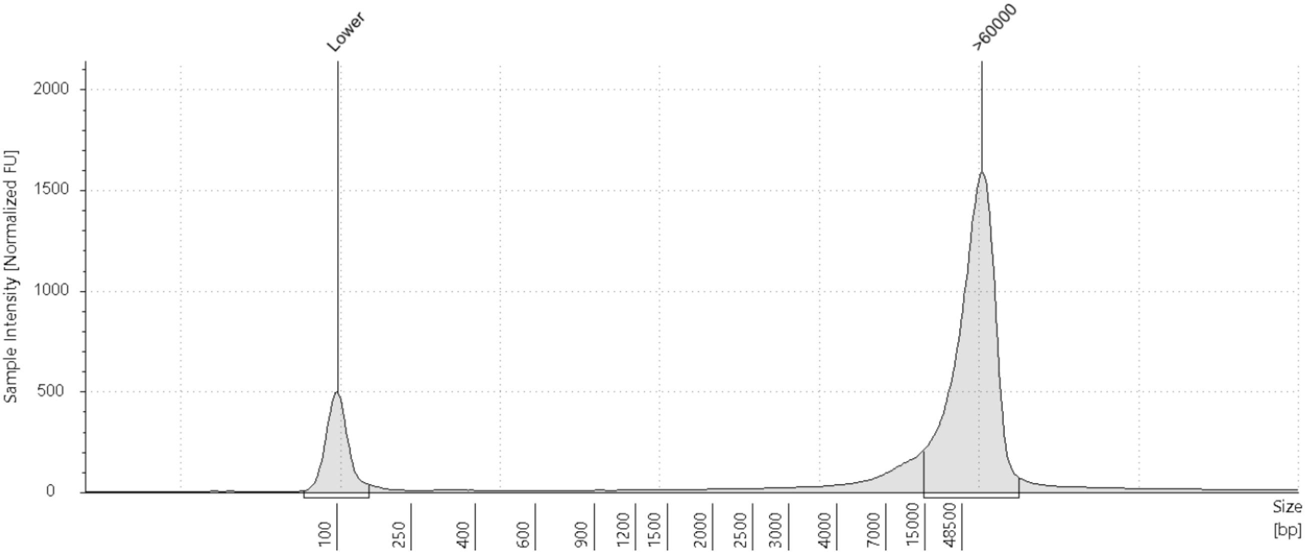

C1: [redacted] Individual 4_Iso 4_4°C

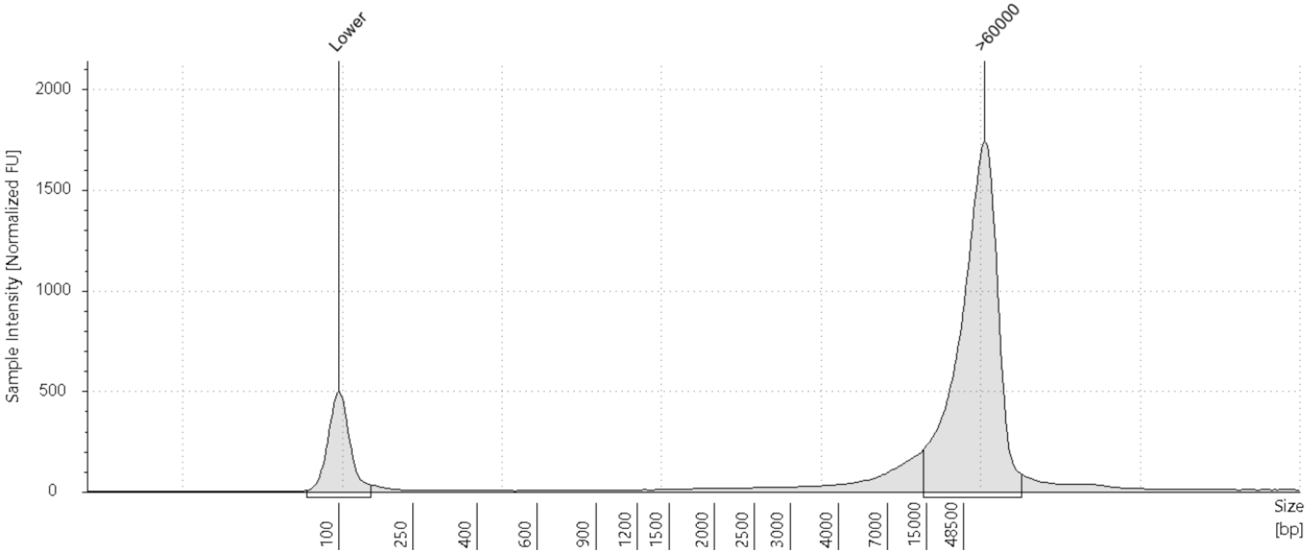

D1: [redacted] Individual 1_Iso 1_4-20°C

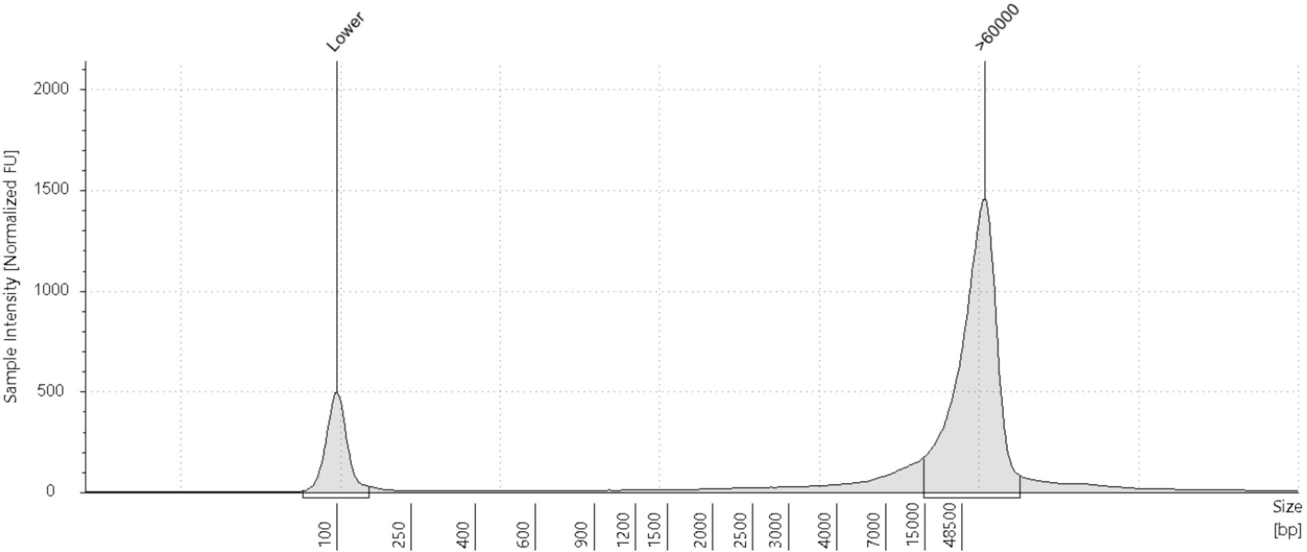

E1: [redacted] Individual 1_Iso 2_4-20°C

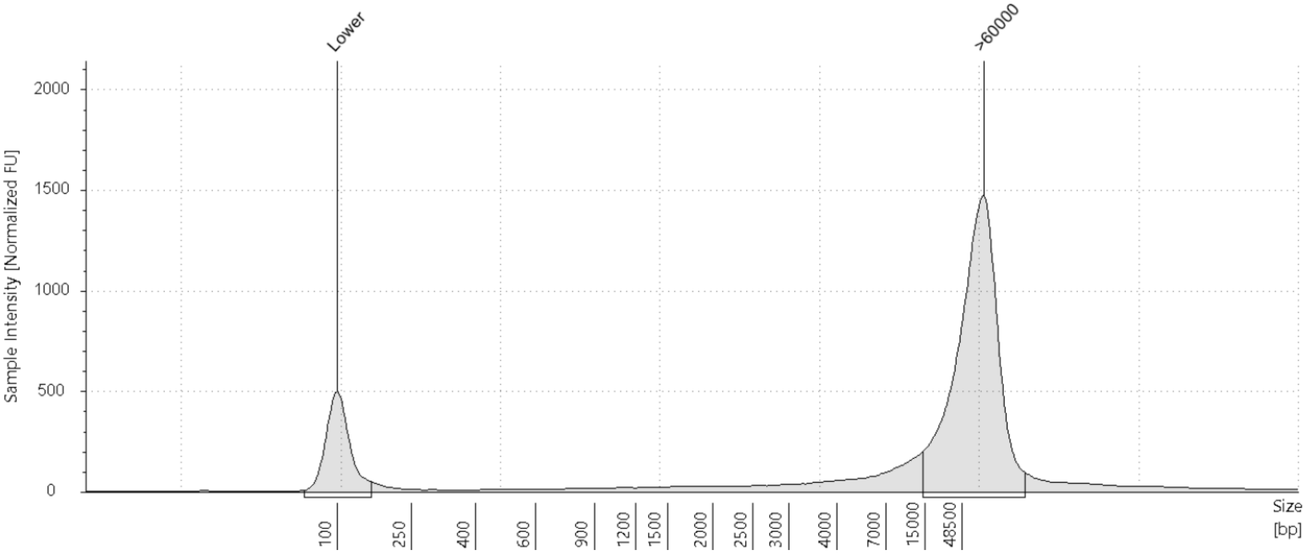

F1: [redacted] Individual 1_Iso 3_4-20°C

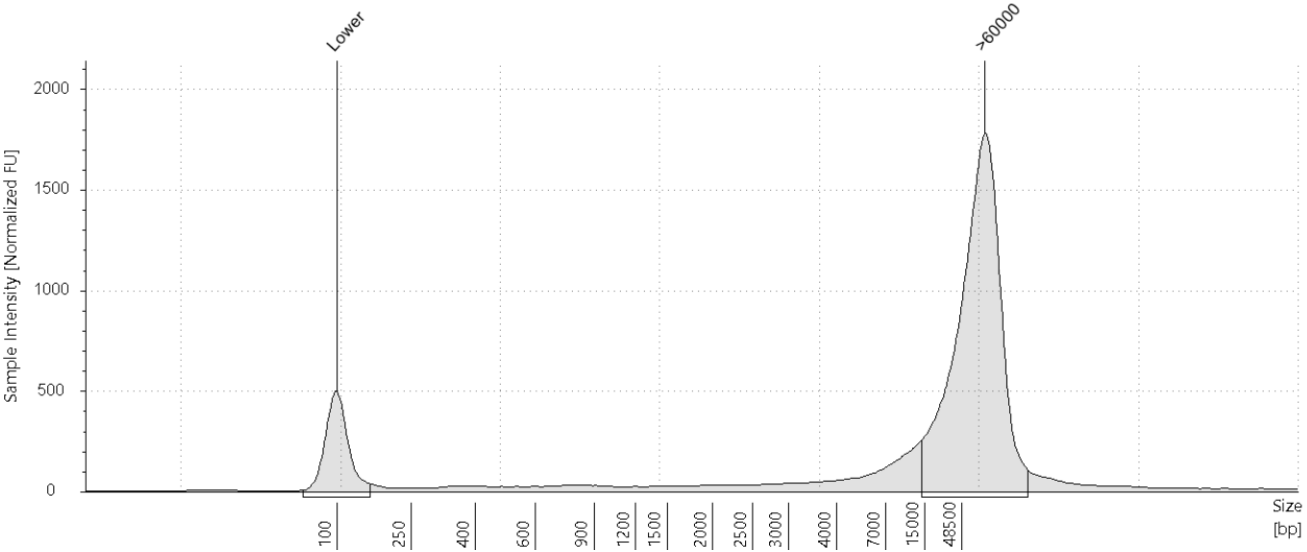

G1: [redacted] Individual 1_Iso 4_4-20°C

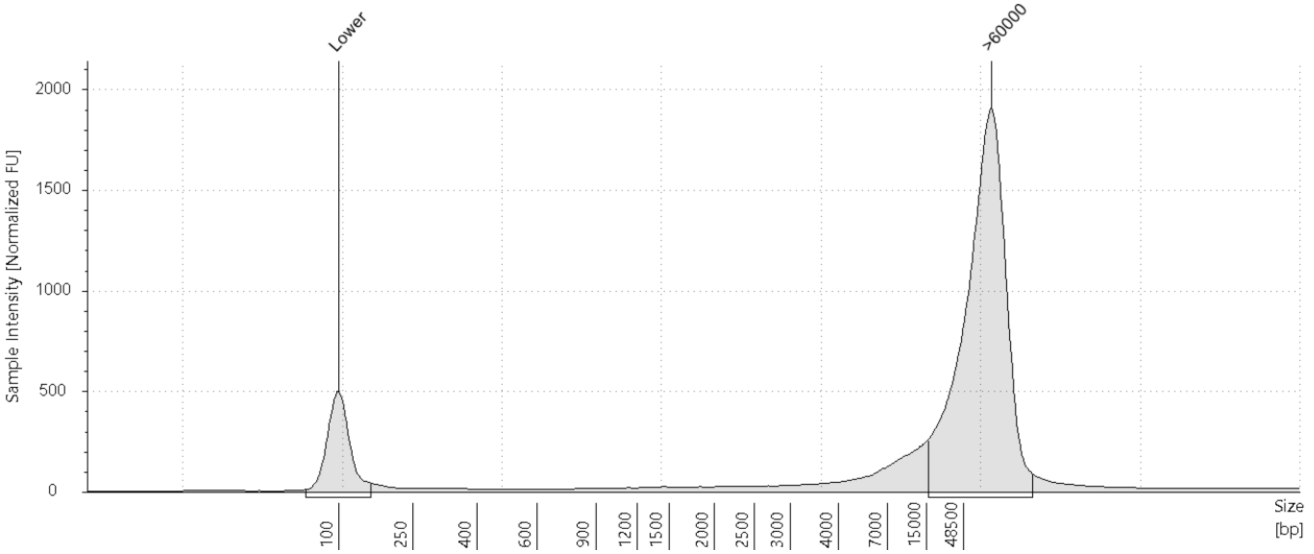

H1: [redacted] Individual 2_Iso 1_4-20°C

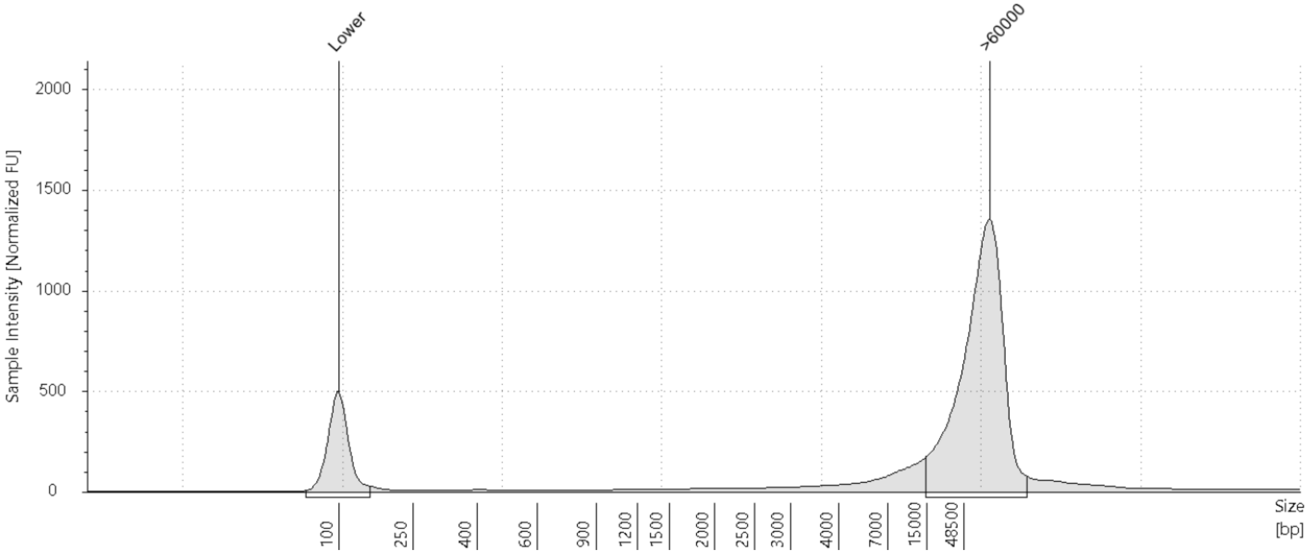

A2: [redacted] Individual 2_Iso 2_4-20°C

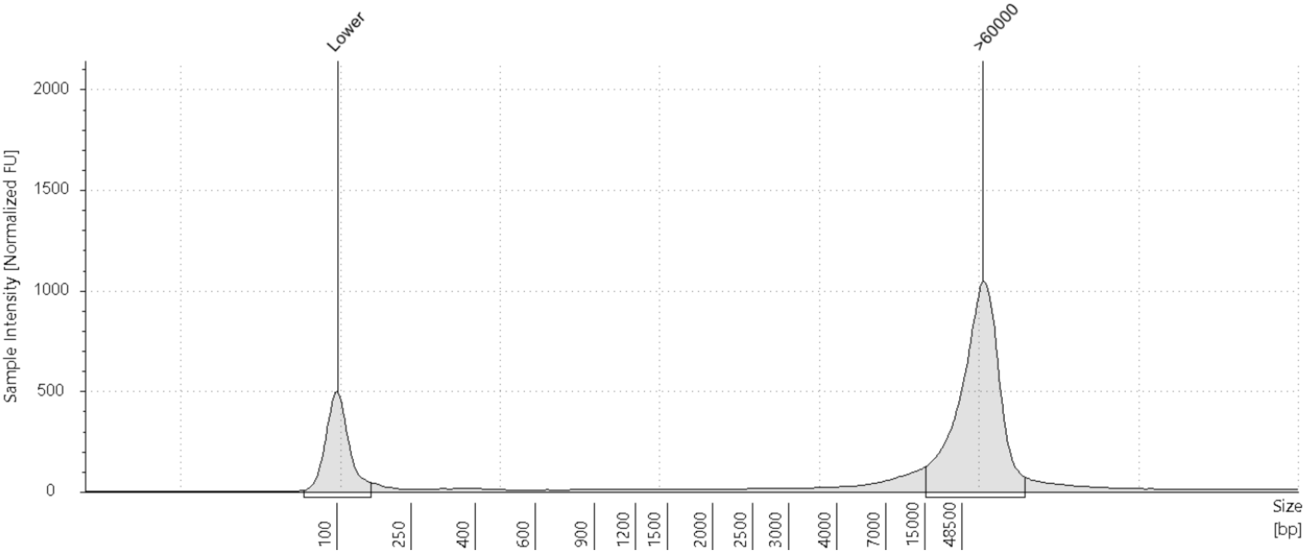

B2: [redacted] Individual 2_Iso 3_4-20°C

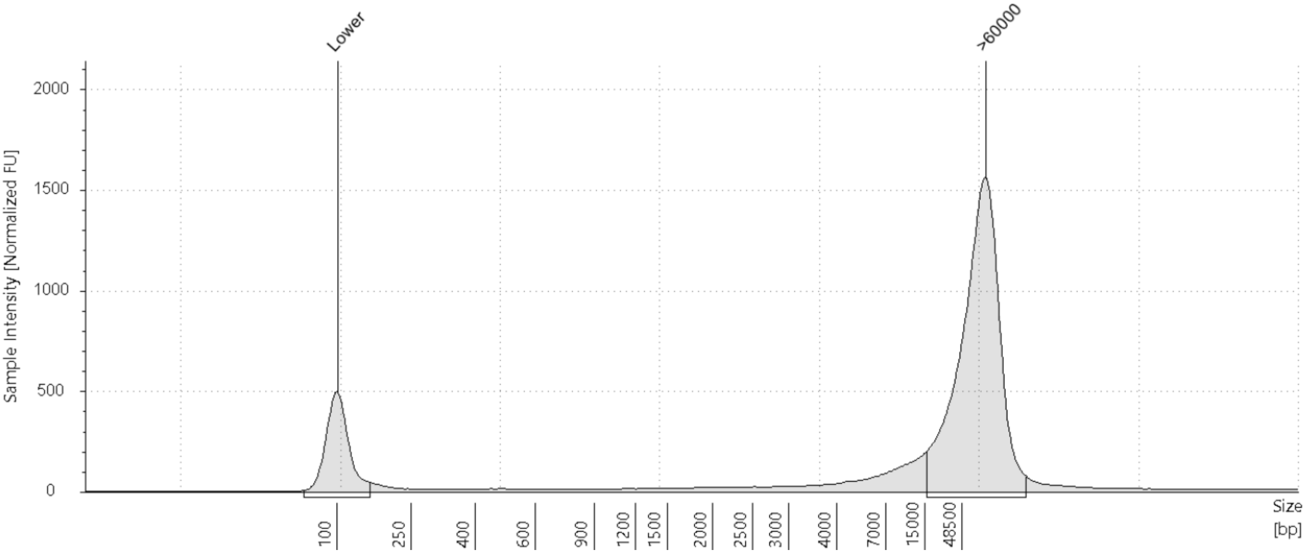

C2: [redacted] Individual 2_Iso 4_4-20°C

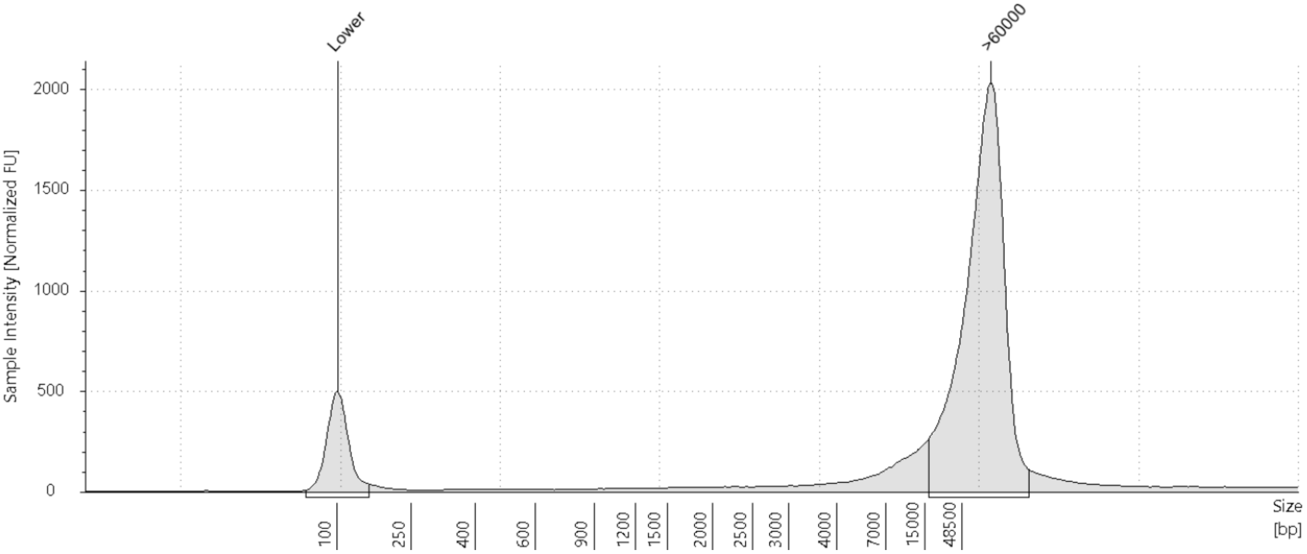

D2: [redacted] Individual 3_Iso 1_4-20°C

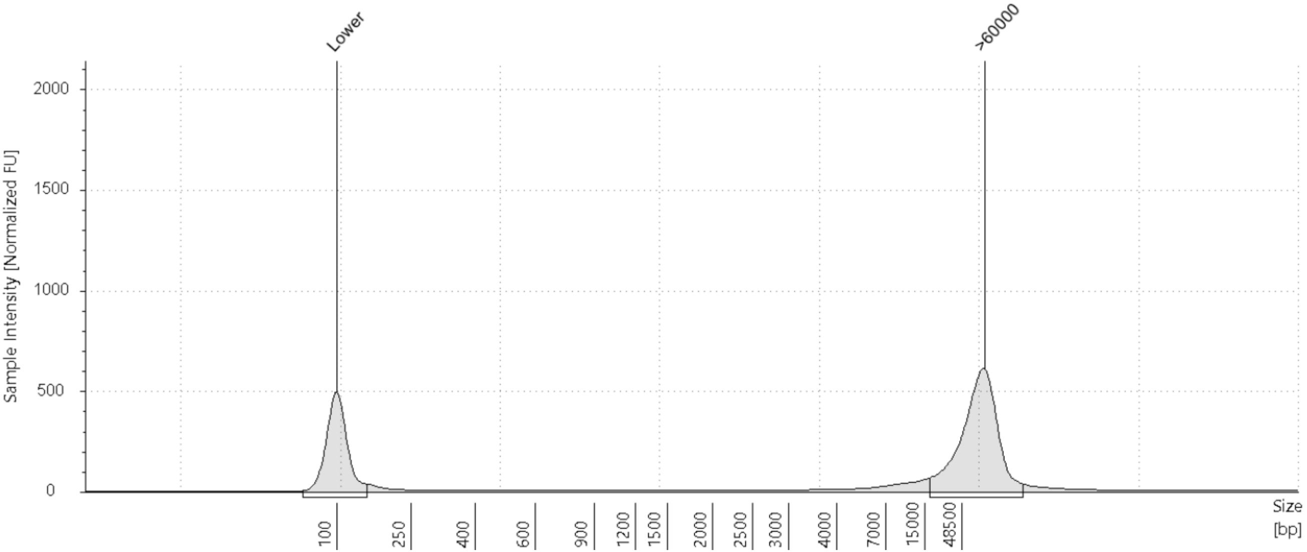

E2: [redacted] Individual 3_Iso 2_4-20°C

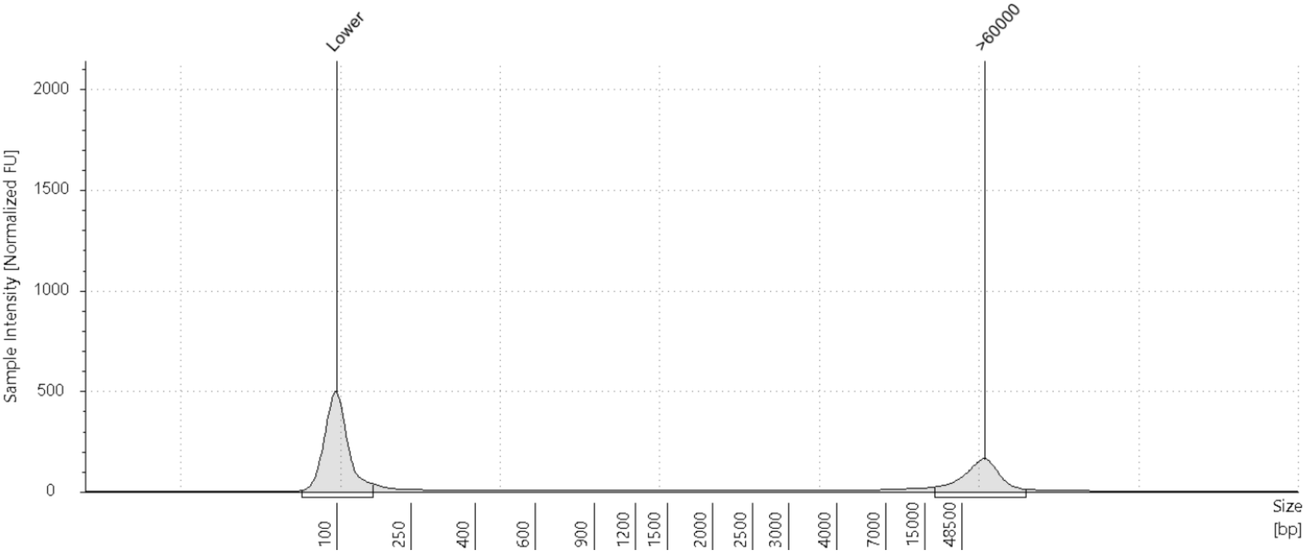

F2: [redacted] Individual 3_Iso 3_4-20°C

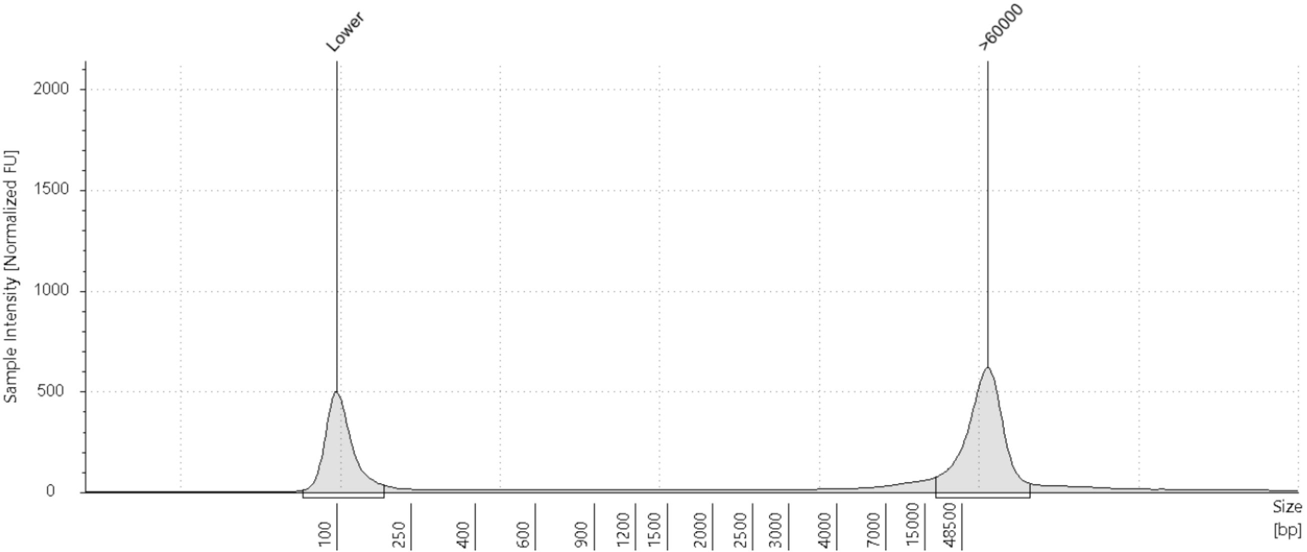

G2: [redacted] Individual 3_Iso 4_4-20°C

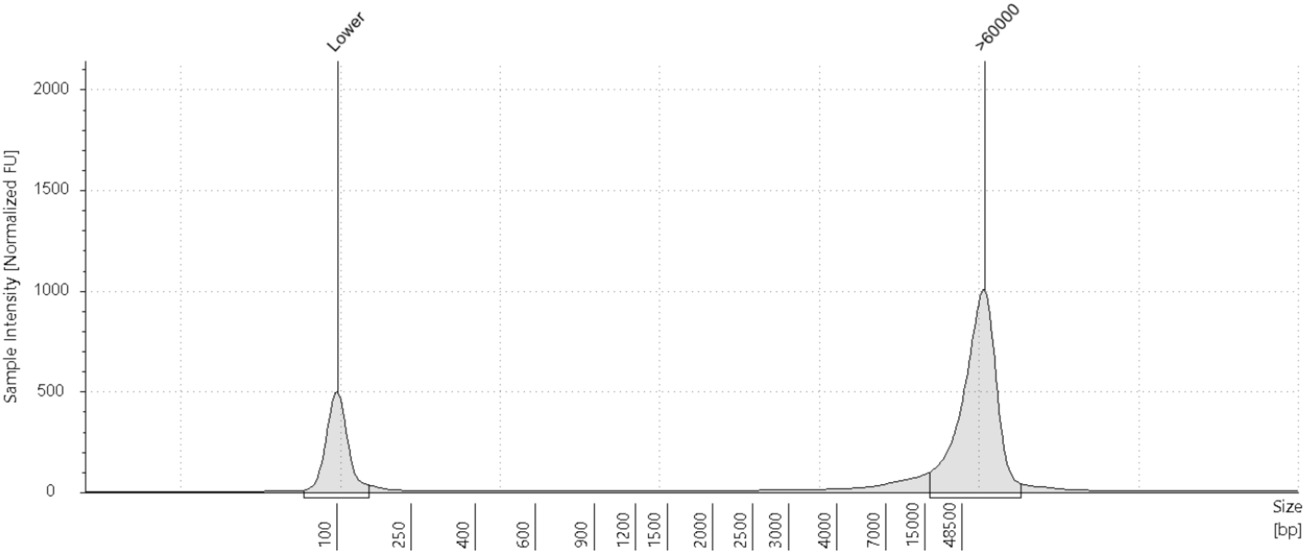

H2: [redacted] Individual 4_Iso 1_4-20°C

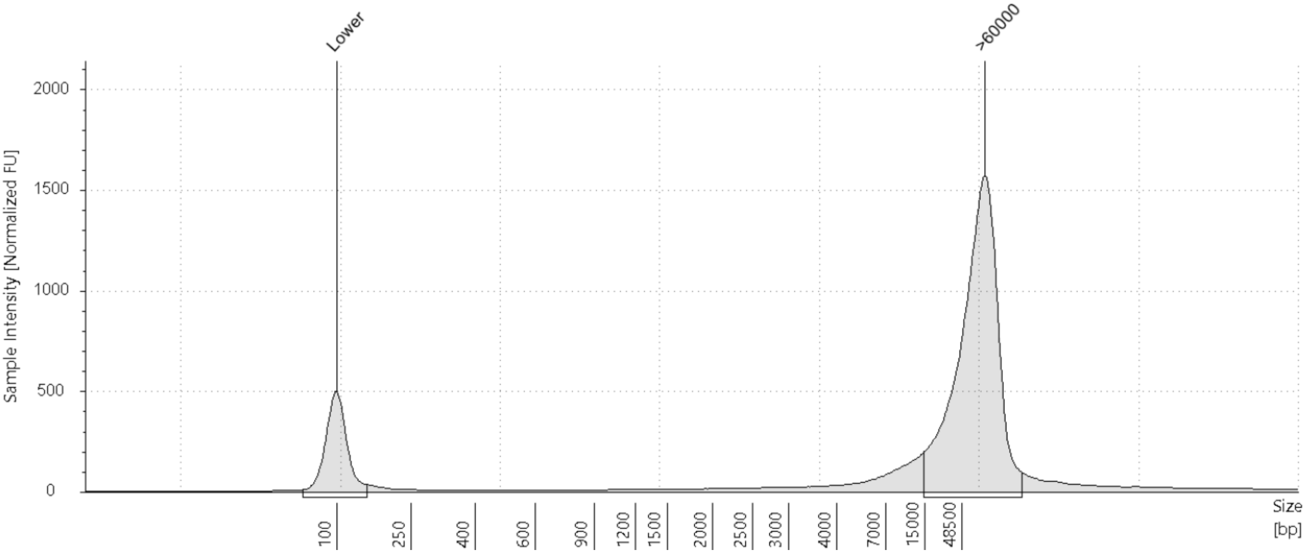

B1: [redacted] Individual 4_Iso 2_4-20°C

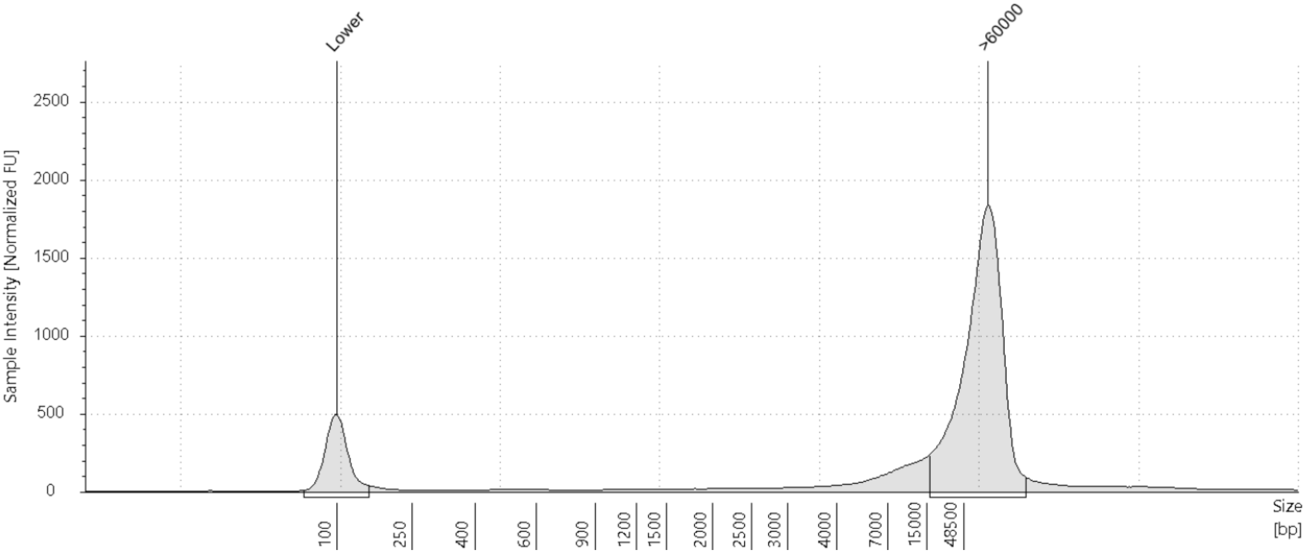

C1: [redacted] Individual 4_Iso 3_4-20°C

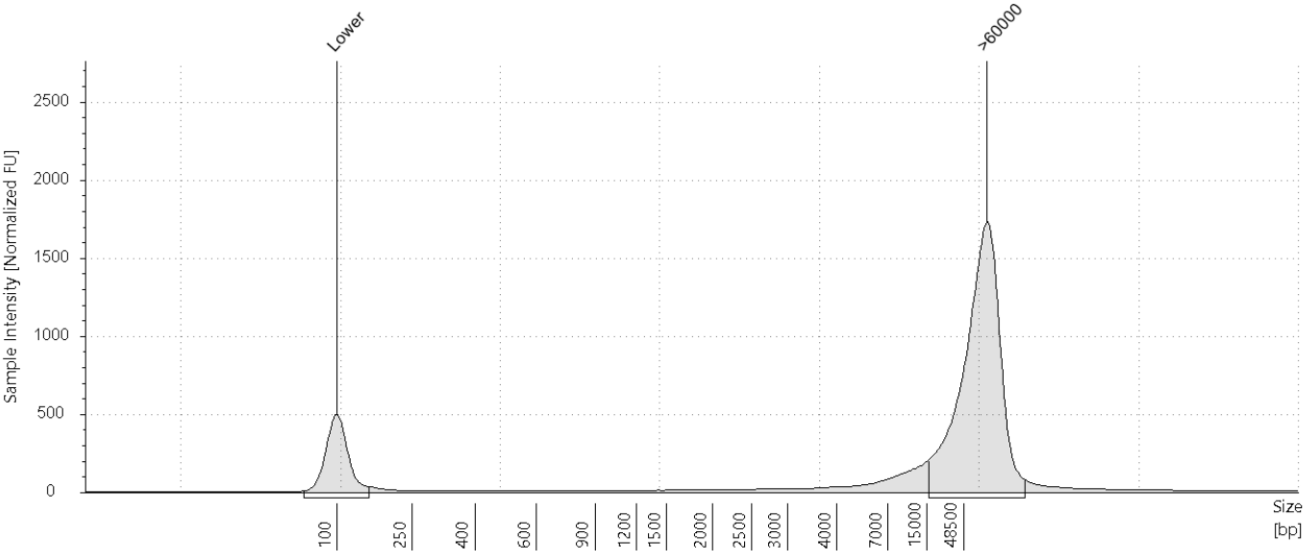

D1: [redacted] Individual 4_Iso 4_4-20°C

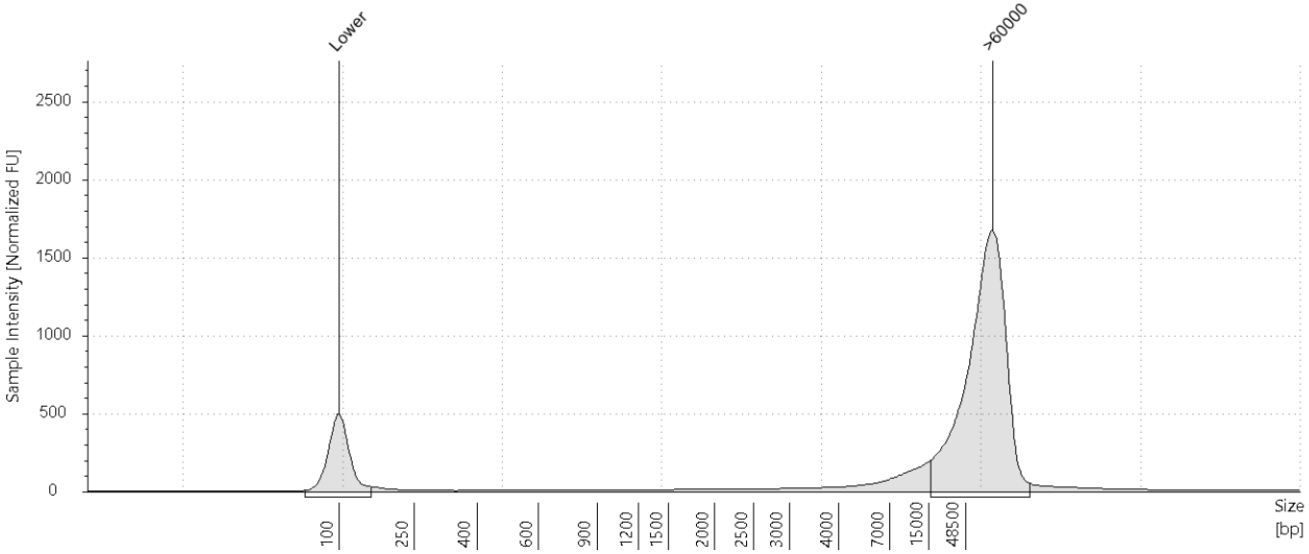

E1: [redacted] Individual 1_Iso 1_-20°C

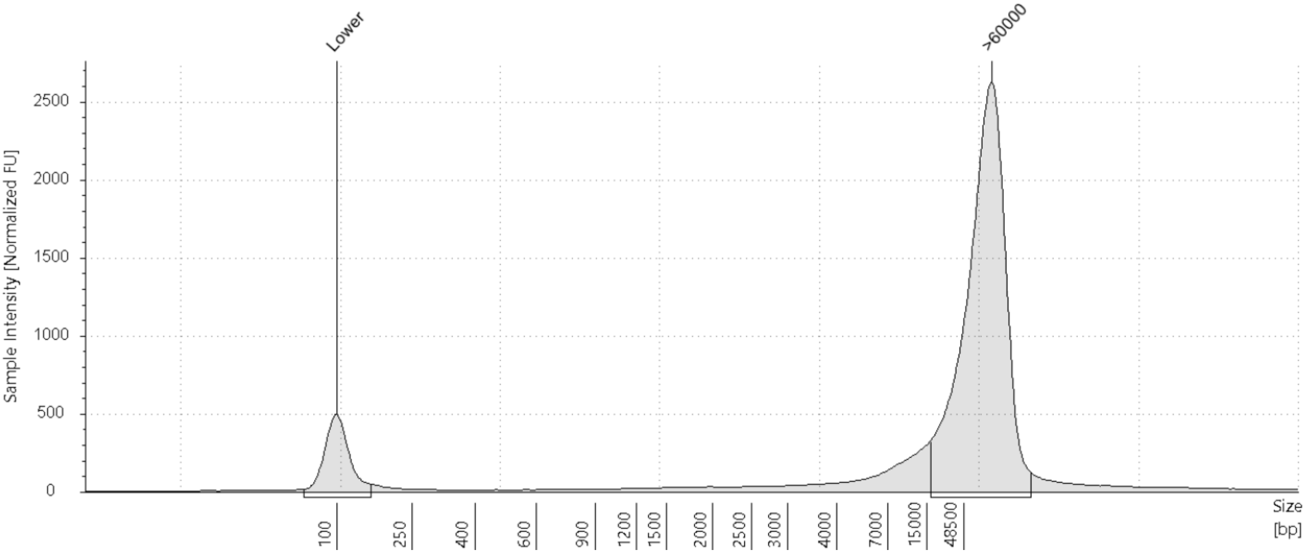

F1: [redacted] Individual 1_Iso 2_-20°C

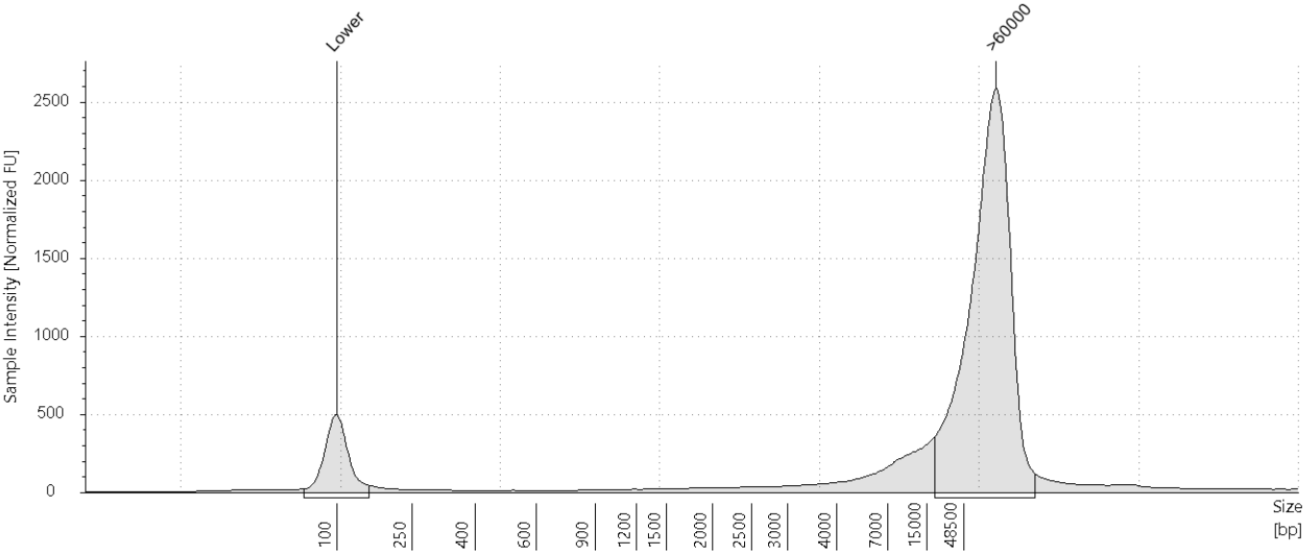

G1: Individual 1_Iso 3_-20°C

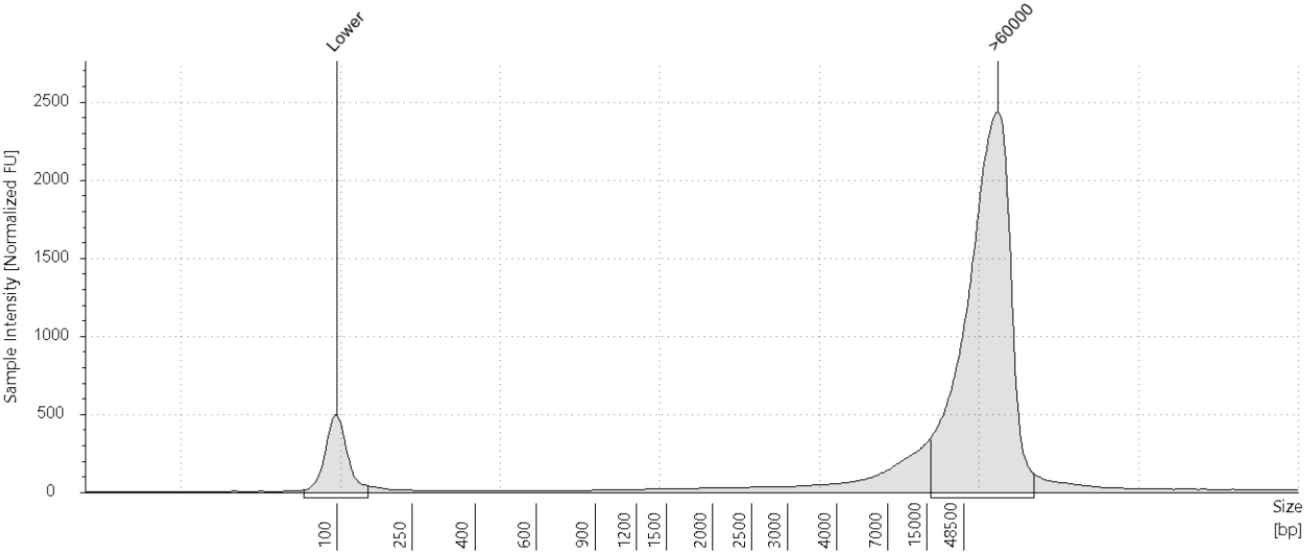

H1: [redacted] Individual 1_Iso 4_-20°C

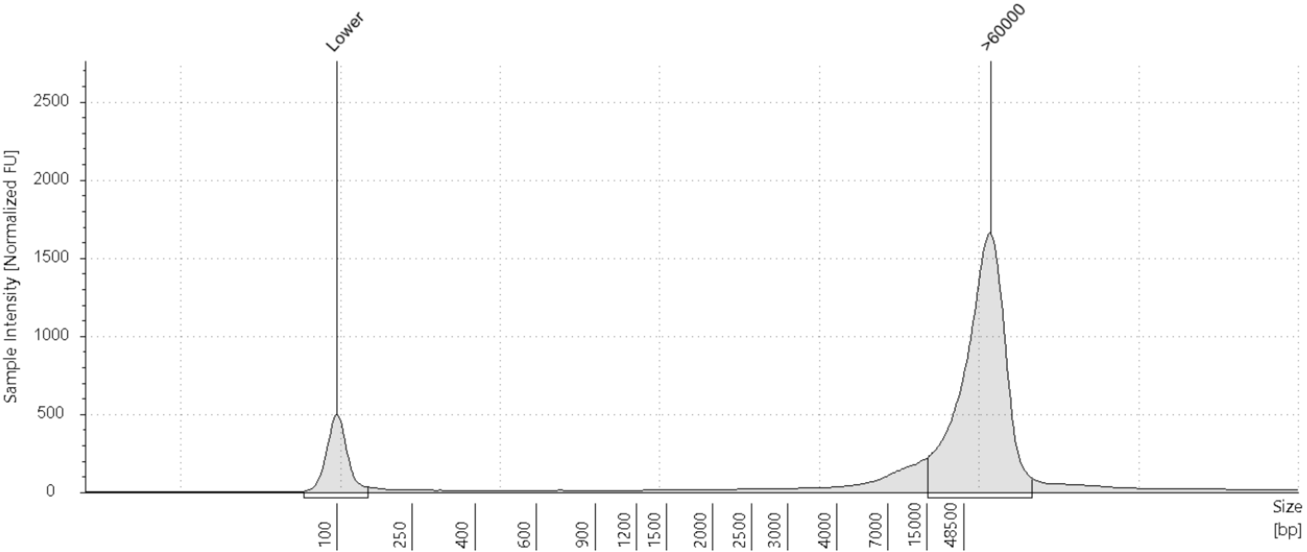

A2: [redacted] Individual 2_Iso 1_-20°C

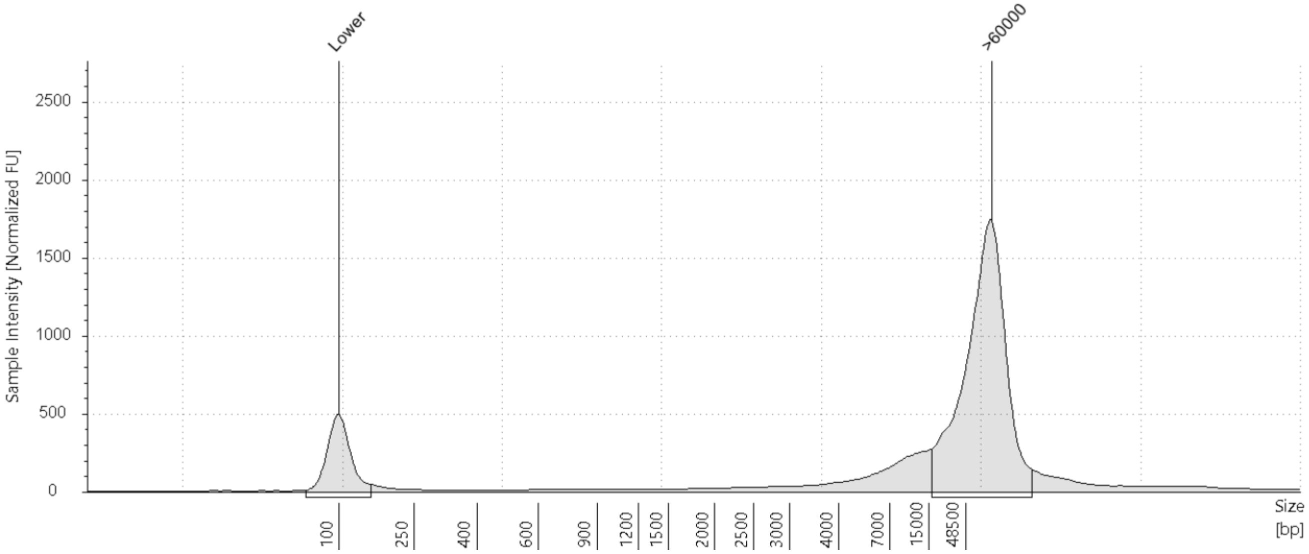

B2: [redacted] Individual 2_Iso 2_-20°C

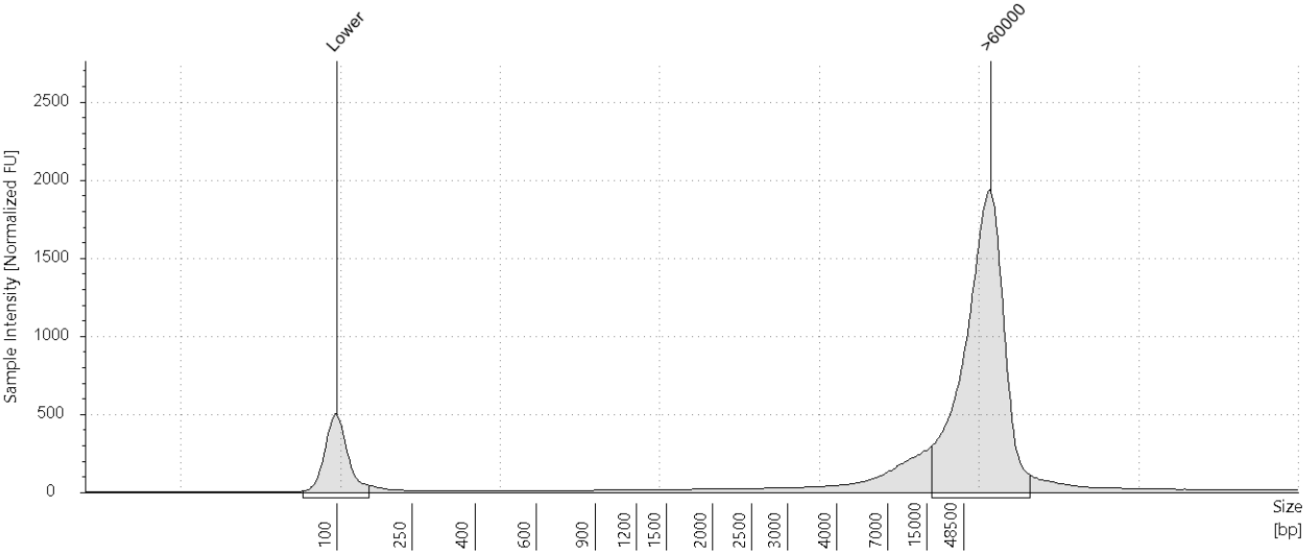

C2: [redacted] Individual 2_Iso 3_-20°C

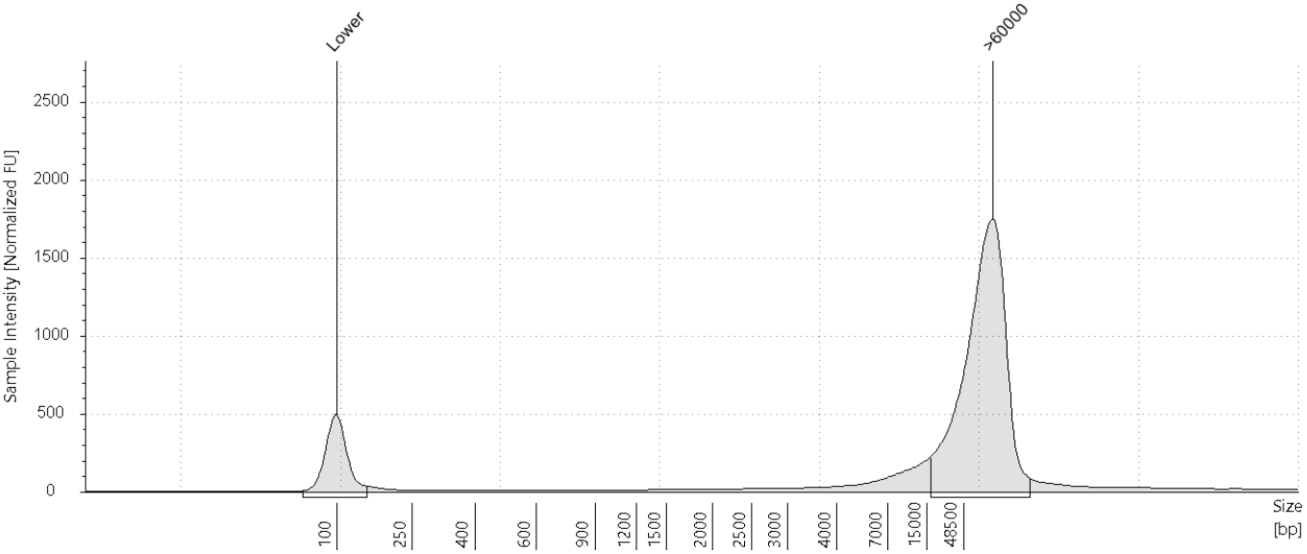

D2: [redacted] Individual 2_Iso 4_-20°C

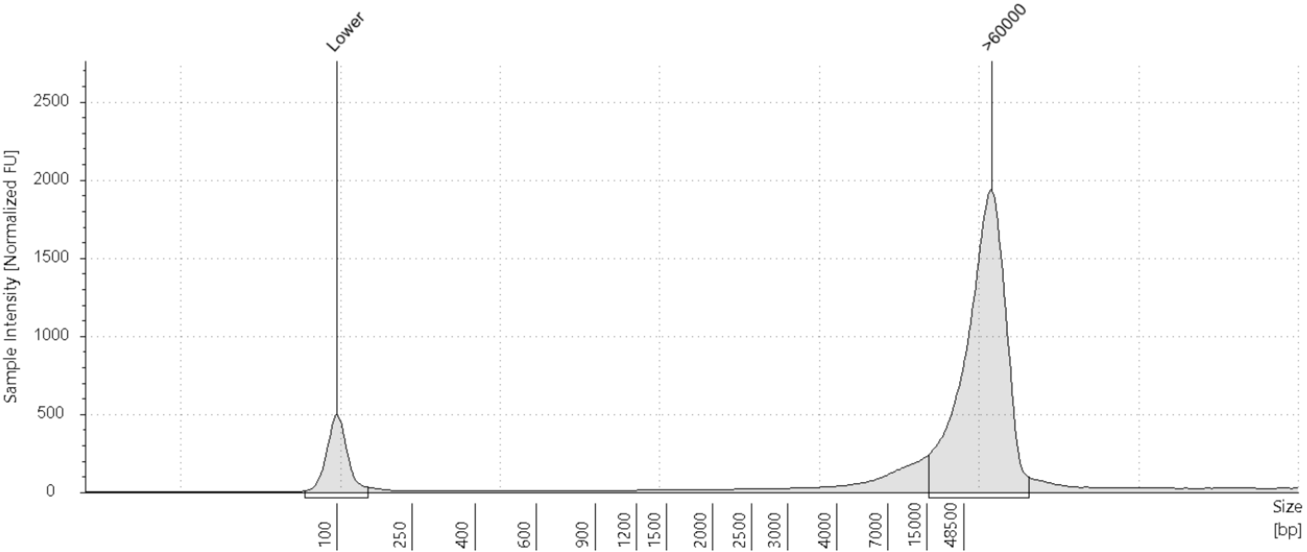

E2: [redacted] Individual 3_Iso 1_-20°C

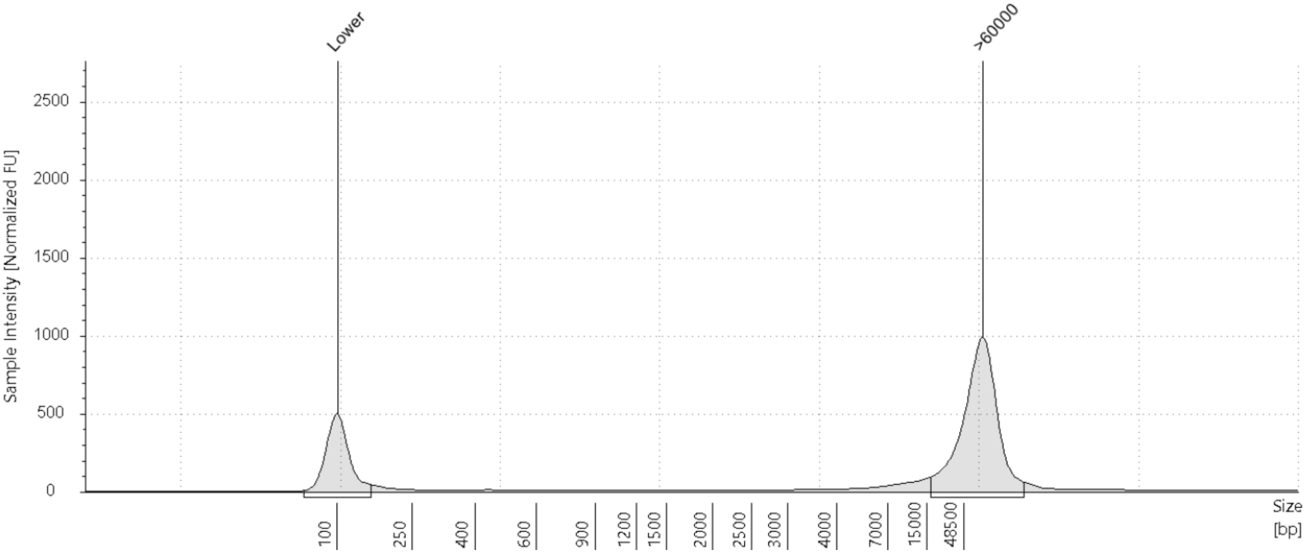

F2: [redacted] Individual 3_Iso 2_-20°C

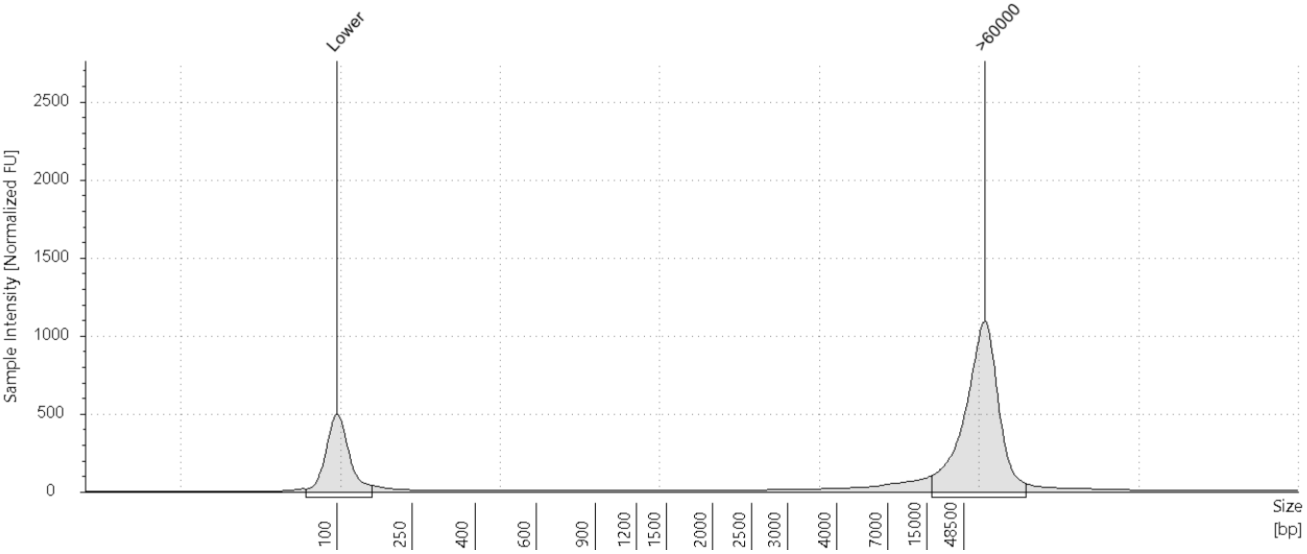

G2: [redacted] Individual 3_Iso 3_-20°C

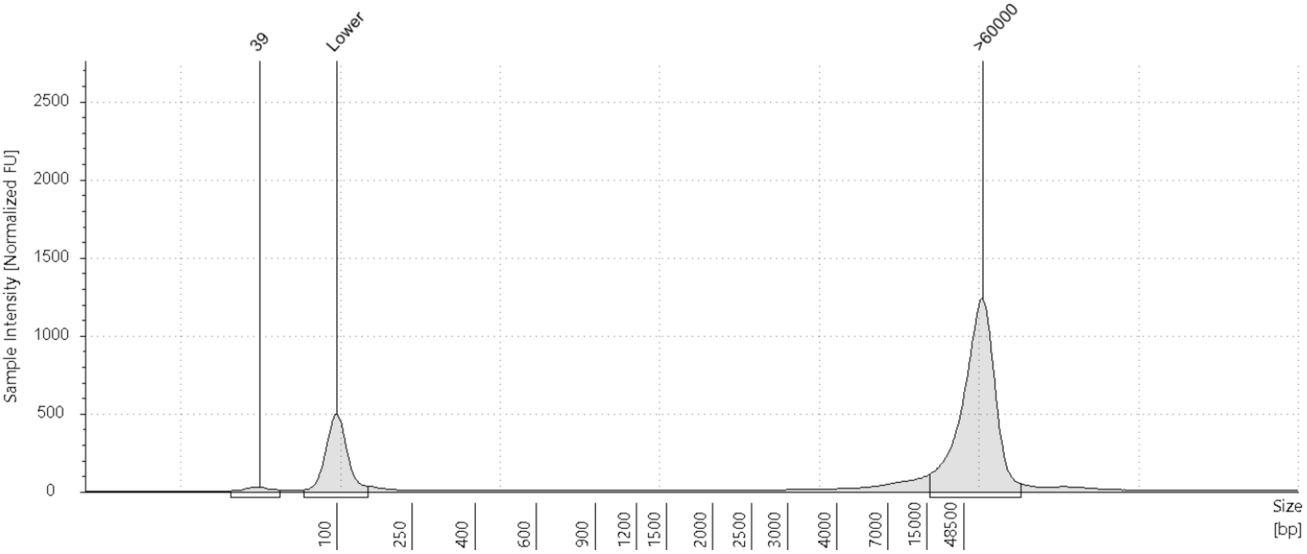

H2: [redacted] Individual 3_Iso 4_-20°C

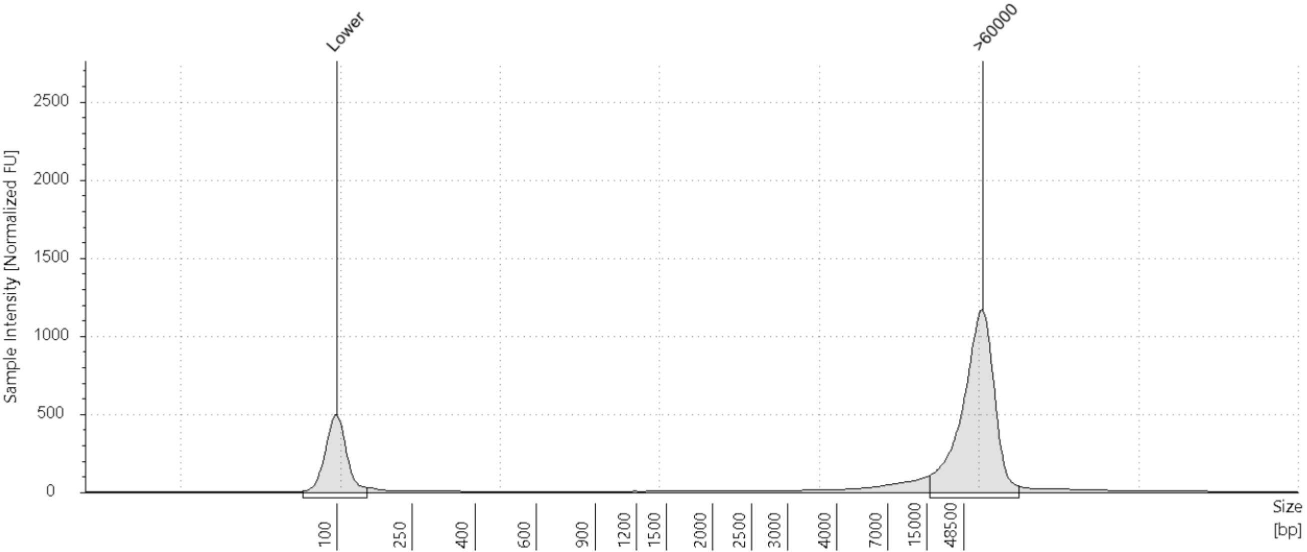

B1: [redacted] Individual 4_Iso 1_-20°C

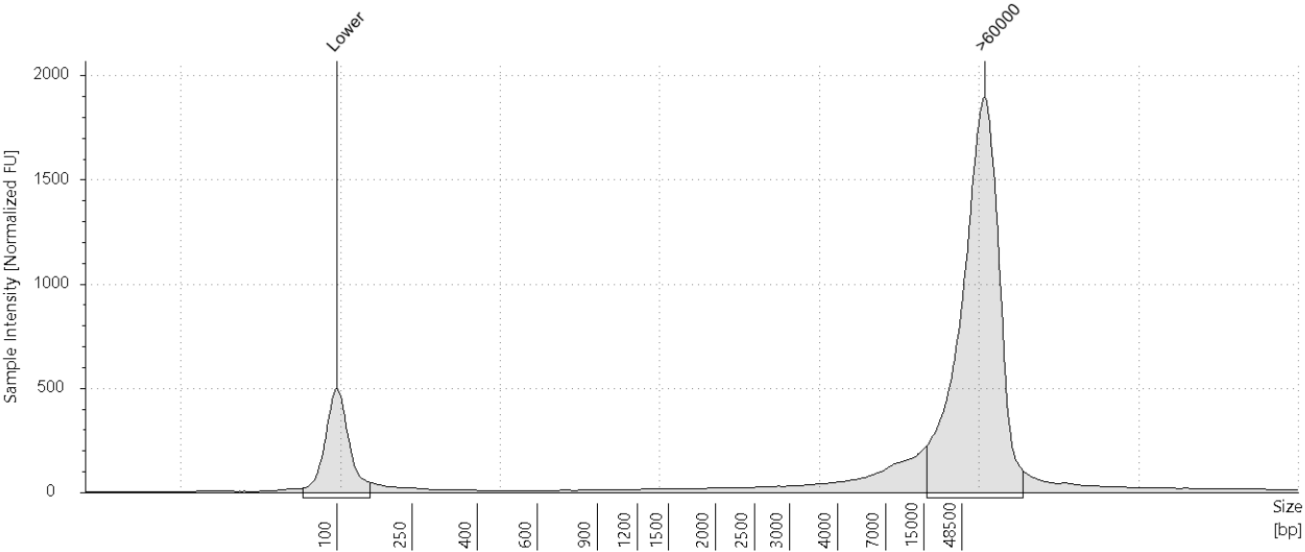

C1: [redacted] Individual 4_Iso 2_-20°C

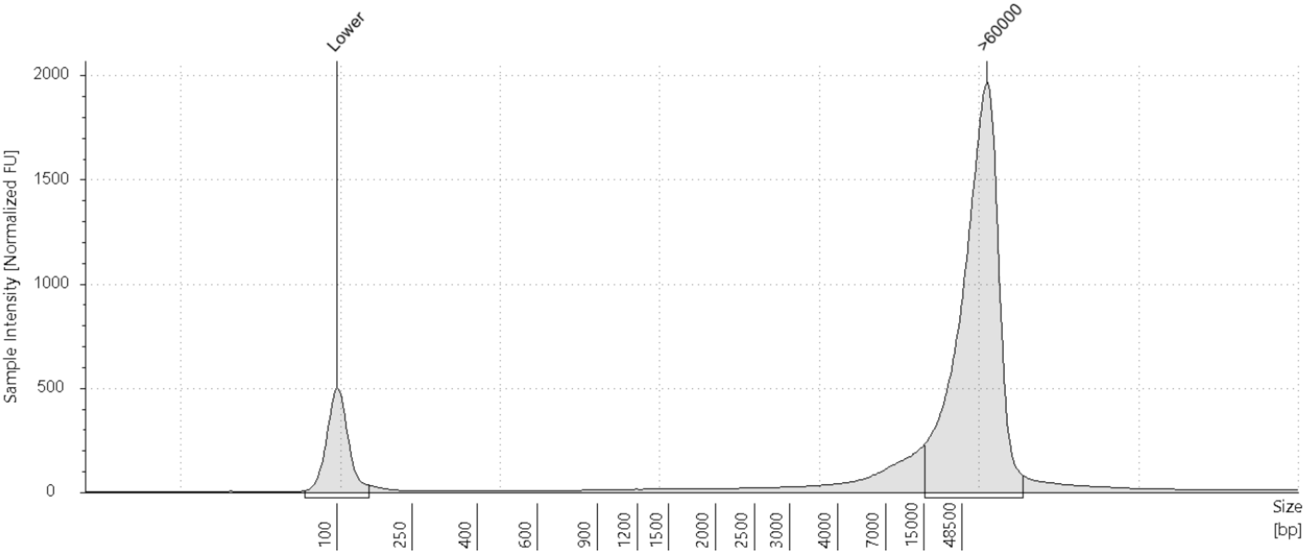

D1: [redacted] Individual 4_Iso 3_-20°C

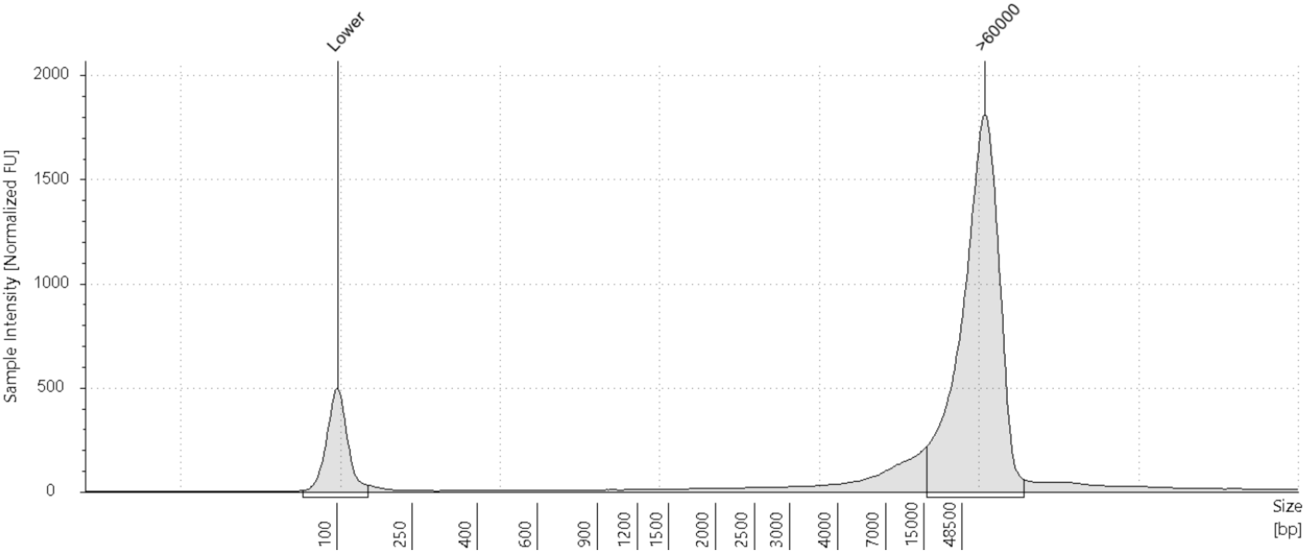

E1: [redacted] Individual 4_Iso 4_-20°C

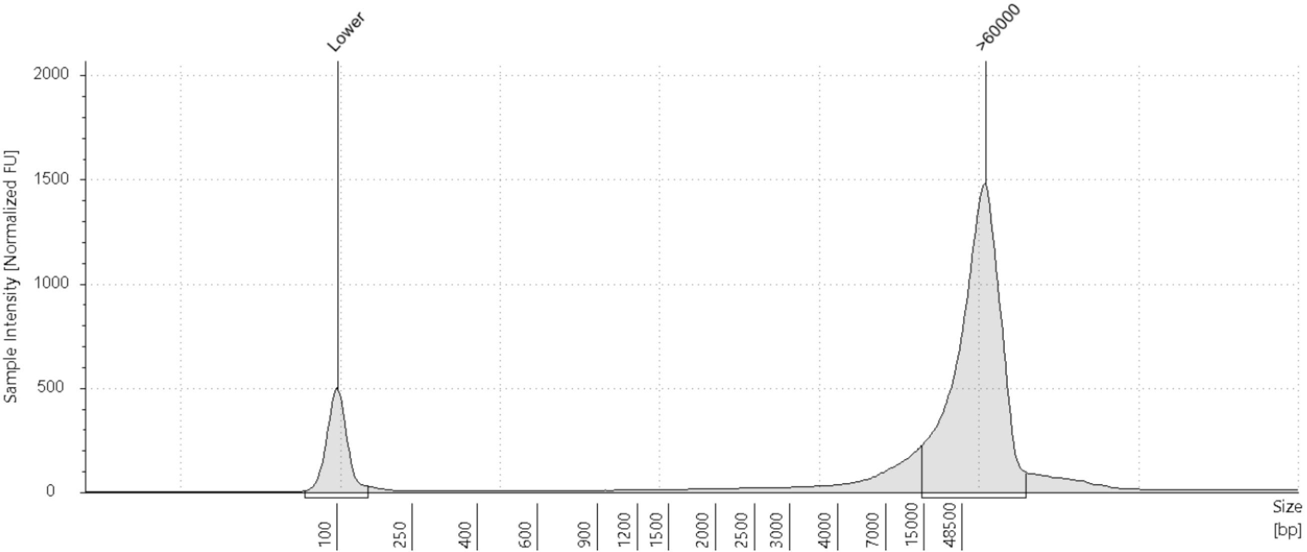

Supplement: Supplementary file 2 — Appendix S2 [file JCLA-38-e25029-s001.pdf]
