# Supplementary material for: Evaluation of Automated Magnetic Bead–Based DNA Extraction for Detection of Short Tandem Repeat Expansions With Nanopore Sequencing
Source: J Clin Lab Anal. 2024 Mar 20;38(6):e25029. doi: 10.1002/jcla.25029 (PMC10997813; doi:10.1002/jcla.25029)
Supplement: Supplementary file 3 — Appendix S3 [file JCLA-38-e25029-s003.html]

NanoComp Report

- Summary Statistics
- Plots
  - Comparing number of reads
  - Comparing throughput in bases
  - Comparing read length N50
  - Comparing read length
  - Comparing log-transformed read length
  - Comparing average base call quality score
  - Histogram of read lengths
  - Normalized histogram of read lengths
  - Weighted histogram of read lengths
  - Histogram of log transformed read lengths
  - Normalized histogram of log transformed read lengths
  - Weighted histogram of log transformed read lengths
- Report issue on Github

# NanoComp report

## Summary statistics

| feature |  |  |  |  |  |  |  |  |  |  |  |  |  |  |  |  |  |  |  |  |
| --- | --- | --- | --- | --- | --- | --- | --- | --- | --- | --- | --- | --- | --- | --- | --- | --- | --- | --- | --- | --- |
| General summary | Ind\_1\_Fresh\_blood | Ind\_1\_Room\_temp | Ind\_1\_Cooled | Ind\_1\_Frozen\_3x\_thawing | Ind\_1\_Frozen | Ind\_2\_Fresh\_blood | Ind\_2\_Room\_temp | Ind\_2\_Cooled | Ind\_2\_Frozen\_3x\_thawing | Ind\_2\_Frozen | Ind\_3\_Fresh\_blood | Ind\_3\_Room\_temp | Ind\_3\_Cooled | Ind\_3\_Frozen\_3x\_thawing | Ind\_3\_Frozen | Ind\_4\_Fresh\_blood | Ind\_4\_Room\_temp | Ind\_4\_Cooled | Ind\_4\_Frozen\_3x\_thawing | Ind\_4\_Frozen |
| Mean read length | 5,436.1 | 2,371.8 | 5,739.7 | 5,578.7 | 5,731.9 | 5,550.1 | 2,388.9 | 5,908.4 | 5,160.2 | 5,892.2 | 6,588.7 | 3,150.7 | 4,587.4 | 5,637.1 | 5,640.6 | 5,556.4 | 2,527.0 | 5,580.2 | 5,151.8 | 5,506.6 |
| Mean read quality | 13.7 | 13.7 | 13.6 | 13.6 | 13.6 | 13.6 | 13.7 | 13.6 | 13.5 | 13.6 | 13.6 | 13.7 | 13.6 | 13.6 | 13.6 | 13.6 | 13.7 | 13.6 | 13.6 | 13.6 |
| Median read length | 3,101.0 | 1,673.0 | 3,279.0 | 3,022.0 | 3,181.0 | 3,225.5 | 1,743.0 | 3,363.5 | 2,853.0 | 3,178.0 | 3,803.0 | 2,122.0 | 3,253.0 | 3,447.0 | 3,444.0 | 3,253.0 | 1,823.0 | 3,249.0 | 3,075.0 | 3,203.0 |
| Median read quality | 14.8 | 14.8 | 14.7 | 14.8 | 14.7 | 14.8 | 14.8 | 14.8 | 14.6 | 14.7 | 14.7 | 14.8 | 14.8 | 14.7 | 14.7 | 14.7 | 14.8 | 14.8 | 14.8 | 14.8 |
| Number of reads | 13,404.0 | 102,737.0 | 11,808.0 | 10,373.0 | 13,267.0 | 10,854.0 | 76,026.0 | 15,520.0 | 9,363.0 | 9,678.0 | 8,437.0 | 77,988.0 | 8,691.0 | 9,317.0 | 9,621.0 | 22,792.0 | 191,301.0 | 14,911.0 | 30,183.0 | 33,125.0 |
| Read length N50 | 8,415.0 | 2,528.0 | 9,317.0 | 9,553.0 | 9,467.0 | 8,564.0 | 2,580.0 | 9,800.0 | 8,544.0 | 10,058.0 | 10,240.0 | 3,854.0 | 5,679.0 | 8,009.0 | 7,861.0 | 8,365.0 | 2,842.0 | 8,664.0 | 7,685.0 | 8,448.0 |
| STDEV read length | 7,107.8 | 2,903.9 | 7,268.9 | 7,614.6 | 7,544.0 | 7,213.2 | 2,523.2 | 7,215.0 | 6,891.8 | 8,173.2 | 8,414.2 | 3,595.5 | 4,933.8 | 7,009.3 | 7,193.0 | 7,203.6 | 2,538.5 | 7,091.9 | 6,562.8 | 7,120.7 |
| Total bases | 72,865,463.0 | 243,667,489.0 | 67,774,847.0 | 57,867,951.0 | 76,044,613.0 | 60,240,666.0 | 181,615,881.0 | 91,697,596.0 | 48,314,729.0 | 57,024,794.0 | 55,588,838.0 | 245,714,230.0 | 39,869,219.0 | 52,520,770.0 | 54,268,209.0 | 126,640,626.0 | 483,420,167.0 | 83,206,173.0 | 155,497,030.0 | 182,406,878.0 |
| Number, percentage and megabases of reads above quality cutoffs |  |  |  |  |  |  |  |  |  |  |  |  |  |  |  |  |  |  |  |  |
| >Q5 | 13404 (100.0%) 72.9Mb | 102737 (100.0%) 243.7Mb | 11808 (100.0%) 67.8Mb | 10373 (100.0%) 57.9Mb | 13267 (100.0%) 76.0Mb | 10854 (100.0%) 60.2Mb | 76026 (100.0%) 181.6Mb | 15520 (100.0%) 91.7Mb | 9363 (100.0%) 48.3Mb | 9678 (100.0%) 57.0Mb | 8437 (100.0%) 55.6Mb | 77988 (100.0%) 245.7Mb | 8691 (100.0%) 39.9Mb | 9317 (100.0%) 52.5Mb | 9621 (100.0%) 54.3Mb | 22792 (100.0%) 126.6Mb | 191301 (100.0%) 483.4Mb | 14911 (100.0%) 83.2Mb | 30183 (100.0%) 155.5Mb | 33125 (100.0%) 182.4Mb |
| >Q7 | 13404 (100.0%) 72.9Mb | 102737 (100.0%) 243.7Mb | 11808 (100.0%) 67.8Mb | 10373 (100.0%) 57.9Mb | 13267 (100.0%) 76.0Mb | 10854 (100.0%) 60.2Mb | 76026 (100.0%) 181.6Mb | 15520 (100.0%) 91.7Mb | 9363 (100.0%) 48.3Mb | 9678 (100.0%) 57.0Mb | 8437 (100.0%) 55.6Mb | 77988 (100.0%) 245.7Mb | 8691 (100.0%) 39.9Mb | 9317 (100.0%) 52.5Mb | 9621 (100.0%) 54.3Mb | 22792 (100.0%) 126.6Mb | 191301 (100.0%) 483.4Mb | 14911 (100.0%) 83.2Mb | 30183 (100.0%) 155.5Mb | 33125 (100.0%) 182.4Mb |
| >Q10 | 13402 (100.0%) 72.9Mb | 102710 (100.0%) 243.6Mb | 11808 (100.0%) 67.8Mb | 10372 (100.0%) 57.9Mb | 13266 (100.0%) 76.0Mb | 10854 (100.0%) 60.2Mb | 76012 (100.0%) 181.6Mb | 15520 (100.0%) 91.7Mb | 9362 (100.0%) 48.3Mb | 9677 (100.0%) 57.0Mb | 8437 (100.0%) 55.6Mb | 77974 (100.0%) 245.7Mb | 8691 (100.0%) 39.9Mb | 9316 (100.0%) 52.5Mb | 9621 (100.0%) 54.3Mb | 22790 (100.0%) 126.6Mb | 191277 (100.0%) 483.4Mb | 14911 (100.0%) 83.2Mb | 30181 (100.0%) 155.5Mb | 33124 (100.0%) 182.4Mb |
| >Q12 | 11560 (86.2%) 62.7Mb | 87858 (85.5%) 207.6Mb | 10152 (86.0%) 58.1Mb | 8923 (86.0%) 50.2Mb | 11344 (85.5%) 64.7Mb | 9224 (85.0%) 51.0Mb | 65314 (85.9%) 155.9Mb | 13376 (86.2%) 79.0Mb | 7912 (84.5%) 40.4Mb | 8245 (85.2%) 47.9Mb | 7185 (85.2%) 47.5Mb | 66798 (85.7%) 209.6Mb | 7409 (85.2%) 34.0Mb | 7969 (85.5%) 44.9Mb | 8200 (85.2%) 46.5Mb | 19492 (85.5%) 108.8Mb | 163882 (85.7%) 412.8Mb | 12821 (86.0%) 72.2Mb | 25828 (85.6%) 133.0Mb | 28269 (85.3%) 155.1Mb |
| >Q15 | 6315 (47.1%) 34.3Mb | 47759 (46.5%) 110.3Mb | 5394 (45.7%) 30.3Mb | 4869 (46.9%) 26.9Mb | 6038 (45.5%) 33.7Mb | 5065 (46.7%) 27.9Mb | 36003 (47.4%) 84.2Mb | 7188 (46.3%) 42.1Mb | 4130 (44.1%) 20.0Mb | 4460 (46.1%) 25.1Mb | 3844 (45.6%) 24.9Mb | 36580 (46.9%) 113.1Mb | 4029 (46.4%) 18.4Mb | 4281 (45.9%) 23.5Mb | 4360 (45.3%) 25.1Mb | 10458 (45.9%) 57.4Mb | 89868 (47.0%) 222.5Mb | 6913 (46.4%) 38.3Mb | 14083 (46.7%) 71.0Mb | 15233 (46.0%) 81.9Mb |
| Top 5 highest mean basecall quality scores and their read lengths |  |  |  |  |  |  |  |  |  |  |  |  |  |  |  |  |  |  |  |  |
| 1 | 24.5 (1470) | 26.9 (1315) | 24.6 (3186) | 24.3 (1234) | 26.3 (1106) | 25.2 (1098) | 25.9 (1112) | 24.1 (1032) | 25.0 (1461) | 24.3 (2620) | 25.1 (1344) | 25.8 (1234) | 23.9 (4581) | 24.3 (2289) | 25.3 (1274) | 25.2 (1681) | 26.7 (1627) | 25.6 (2201) | 24.9 (1210) | 24.8 (1142) |
| 2 | 24.5 (1580) | 26.1 (1120) | 24.3 (2449) | 24.3 (2147) | 23.9 (1694) | 23.6 (2943) | 25.8 (1069) | 24.1 (1000) | 24.4 (2027) | 24.1 (2638) | 23.4 (1349) | 25.6 (1417) | 23.8 (2162) | 24.1 (1283) | 24.5 (1580) | 24.6 (1093) | 26.4 (1824) | 24.5 (1569) | 24.6 (1020) | 24.4 (2616) |
| 3 | 23.9 (3356) | 25.8 (1157) | 23.7 (1025) | 24.1 (1557) | 23.7 (1211) | 23.6 (1920) | 25.5 (1313) | 23.8 (1516) | 23.5 (1107) | 24.0 (3619) | 22.9 (1836) | 25.5 (1084) | 23.5 (1273) | 24.0 (2012) | 23.7 (1873) | 24.4 (2035) | 26.2 (1747) | 24.0 (1511) | 24.5 (3838) | 24.2 (1348) |
| 4 | 23.0 (1675) | 25.7 (1092) | 23.6 (4712) | 23.7 (3239) | 23.7 (1145) | 23.4 (2636) | 25.1 (1140) | 23.5 (1717) | 23.1 (1507) | 23.8 (1475) | 22.9 (1032) | 25.4 (1593) | 23.4 (4316) | 23.2 (2264) | 23.4 (3412) | 23.9 (1184) | 26.1 (1151) | 23.9 (2159) | 24.5 (1821) | 24.2 (1070) |
| 5 | 23.0 (1193) | 25.7 (1575) | 23.5 (1803) | 23.6 (1469) | 23.7 (2853) | 23.2 (1071) | 25.1 (1155) | 23.5 (3370) | 23.1 (1505) | 23.7 (1842) | 22.7 (1869) | 25.4 (1506) | 23.4 (2594) | 23.1 (3739) | 23.3 (1679) | 23.9 (1154) | 26.1 (1022) | 23.7 (2030) | 24.3 (1525) | 24.2 (4482) |
| Top 5 longest reads and their mean basecall quality score |  |  |  |  |  |  |  |  |  |  |  |  |  |  |  |  |  |  |  |  |
| 1 | 91815 (13.4) | 95570 (10.5) | 104633 (10.2) | 86760 (14.8) | 108048 (10.6) | 84644 (16.6) | 74837 (15.0) | 131356 (10.2) | 75259 (10.6) | 118758 (10.5) | 89261 (12.6) | 104511 (10.8) | 104933 (13.4) | 92698 (14.1) | 88120 (15.0) | 81842 (12.3) | 85306 (12.4) | 106551 (15.7) | 93795 (10.9) | 86112 (15.6) |
| 2 | 80535 (19.6) | 91372 (10.1) | 87076 (15.1) | 72749 (15.1) | 80219 (14.3) | 82177 (10.6) | 72157 (13.9) | 73114 (17.0) | 64083 (10.8) | 95168 (10.2) | 85967 (10.5) | 94266 (15.2) | 68033 (14.8) | 88596 (12.2) | 87593 (15.4) | 81519 (15.8) | 74669 (16.2) | 89135 (10.8) | 93774 (13.7) | 82884 (18.8) |
| 3 | 75972 (11.7) | 86861 (11.1) | 81125 (12.0) | 72371 (14.3) | 73038 (14.1) | 76808 (13.6) | 70076 (15.1) | 72466 (12.3) | 62075 (11.3) | 91815 (13.4) | 84561 (14.7) | 88864 (12.5) | 64199 (10.6) | 75034 (11.8) | 77502 (14.3) | 79862 (12.4) | 71561 (14.2) | 84960 (13.4) | 92541 (13.4) | 82808 (10.4) |
| 4 | 74084 (16.9) | 82247 (12.9) | 79083 (14.9) | 71559 (17.9) | 72947 (15.0) | 72081 (12.0) | 60821 (15.6) | 70727 (15.0) | 61426 (12.6) | 82685 (10.5) | 82541 (17.5) | 85290 (10.5) | 56878 (14.9) | 73852 (16.2) | 77019 (14.2) | 75635 (15.2) | 71349 (15.3) | 84365 (12.4) | 87141 (14.8) | 82646 (15.1) |
| 5 | 67636 (16.3) | 72418 (15.4) | 71671 (11.6) | 70033 (16.1) | 72727 (13.1) | 71256 (11.9) | 60495 (17.0) | 69661 (10.7) | 61287 (14.7) | 77776 (16.0) | 77604 (16.8) | 73055 (13.2) | 56688 (12.6) | 71382 (11.4) | 74392 (15.3) | 74769 (16.6) | 70234 (13.7) | 83901 (17.9) | 84961 (14.9) | 81755 (16.2) |

## Plots

Comparing number of reads

#### Comparing number of reads

Comparing throughput in bases

#### Comparing throughput in bases

Comparing read length N50

#### Comparing read length N50

Comparing read length

#### Comparing read length

Comparing log-transformed read length

#### Comparing log-transformed read length

Comparing average base call quality score

#### Comparing average base call quality score

Histogram of read lengths

#### Histogram of read lengths

Normalized histogram of read lengths

#### Normalized histogram of read lengths

Weighted histogram of read lengths

#### Weighted histogram of read lengths

Histogram of log transformed read lengths

#### Histogram of log transformed read lengths

Normalized histogram of log transformed read lengths

#### Normalized histogram of log transformed read lengths

Weighted histogram of log transformed read lengths

#### Weighted histogram of log transformed read lengths
