# Supplementary material for: Evaluation of Automated Magnetic Bead–Based DNA Extraction for Detection of Short Tandem Repeat Expansions With Nanopore Sequencing
Source: J Clin Lab Anal. 2024 Mar 20;38(6):e25029. doi: 10.1002/jcla.25029 (PMC10997813; doi:10.1002/jcla.25029)

B1: [redacted] sample 1

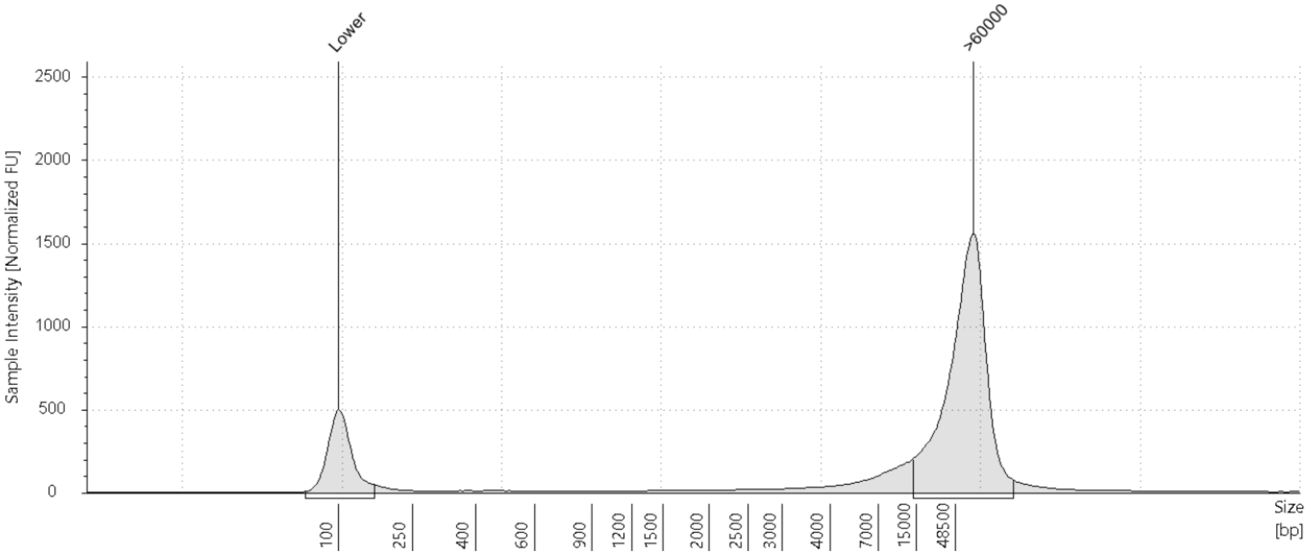

C1: [redacted] sample 2

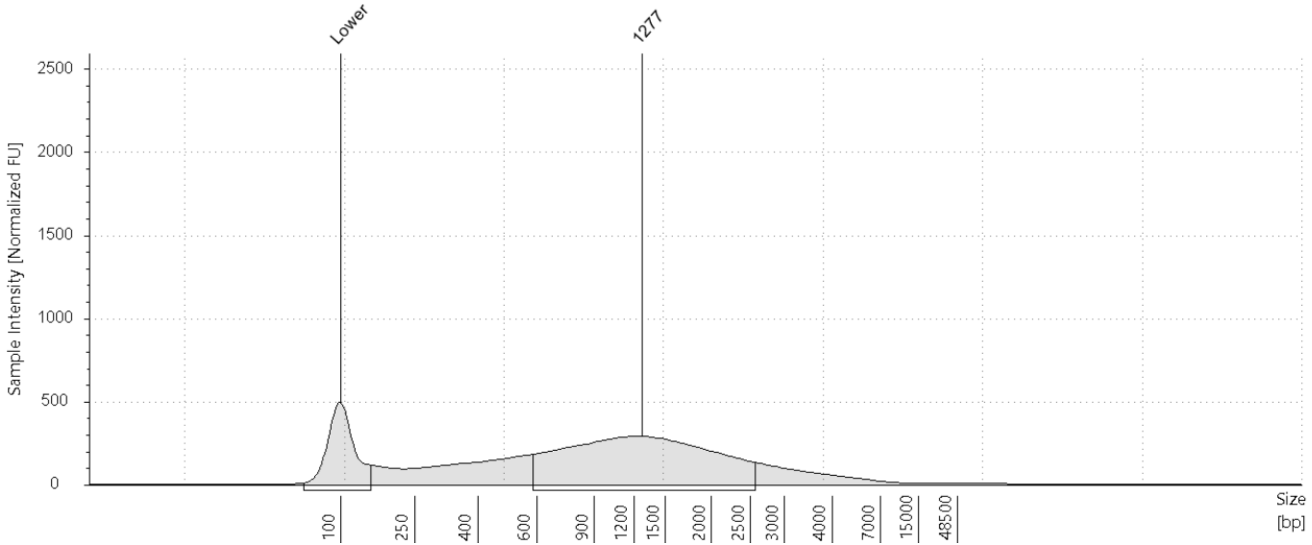

D1: [redacted] sample 3

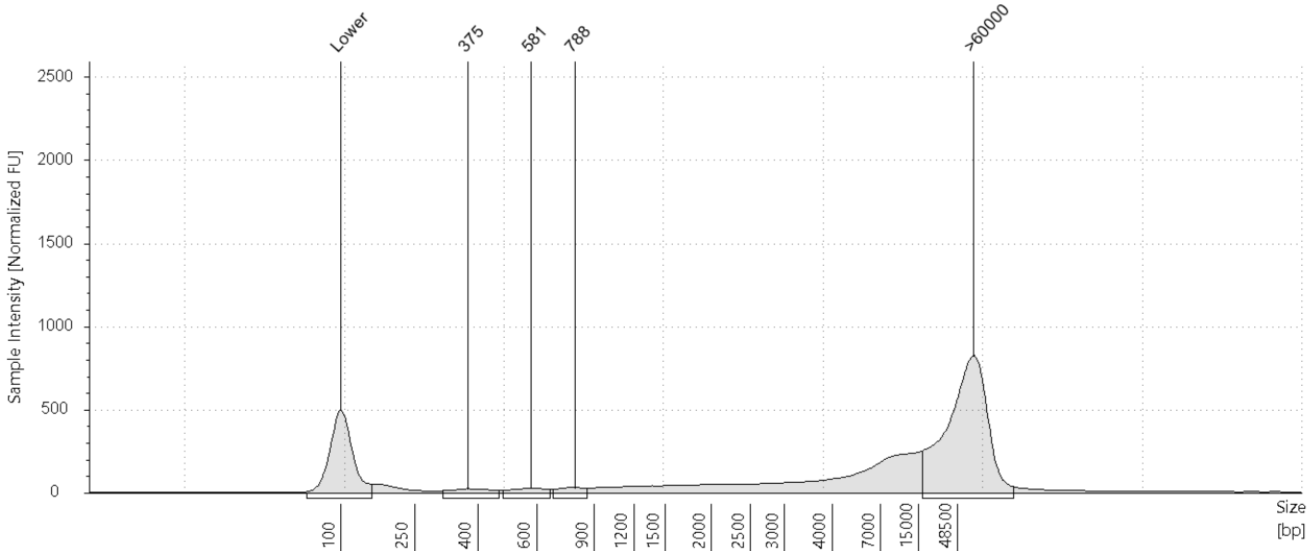

E1: [redacted] sample 4

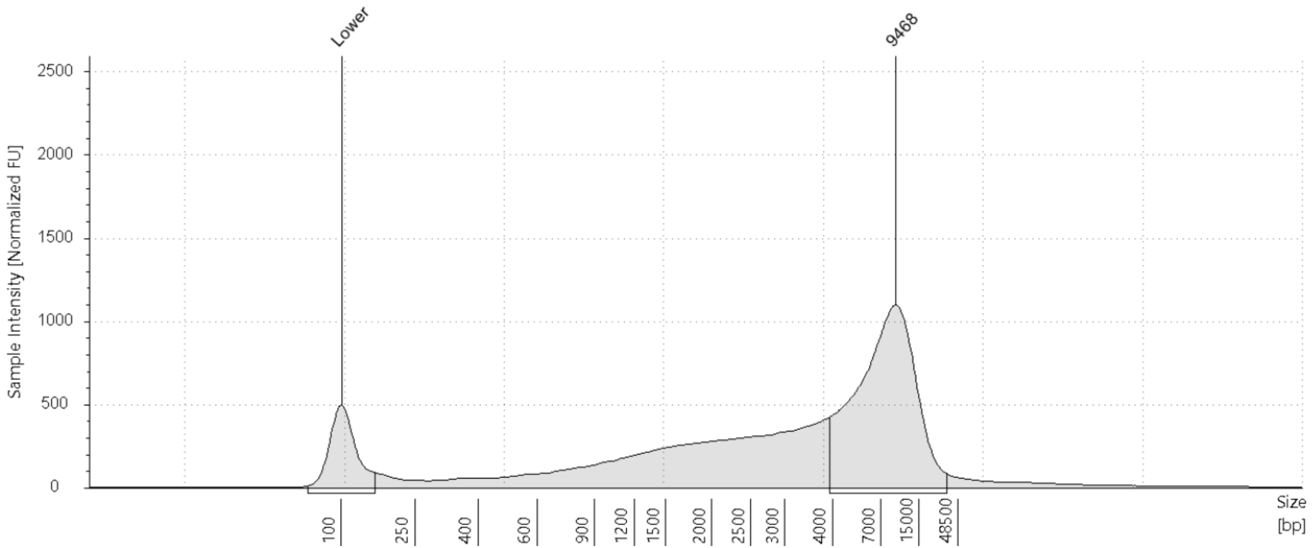

F1: [redacted] sample 5

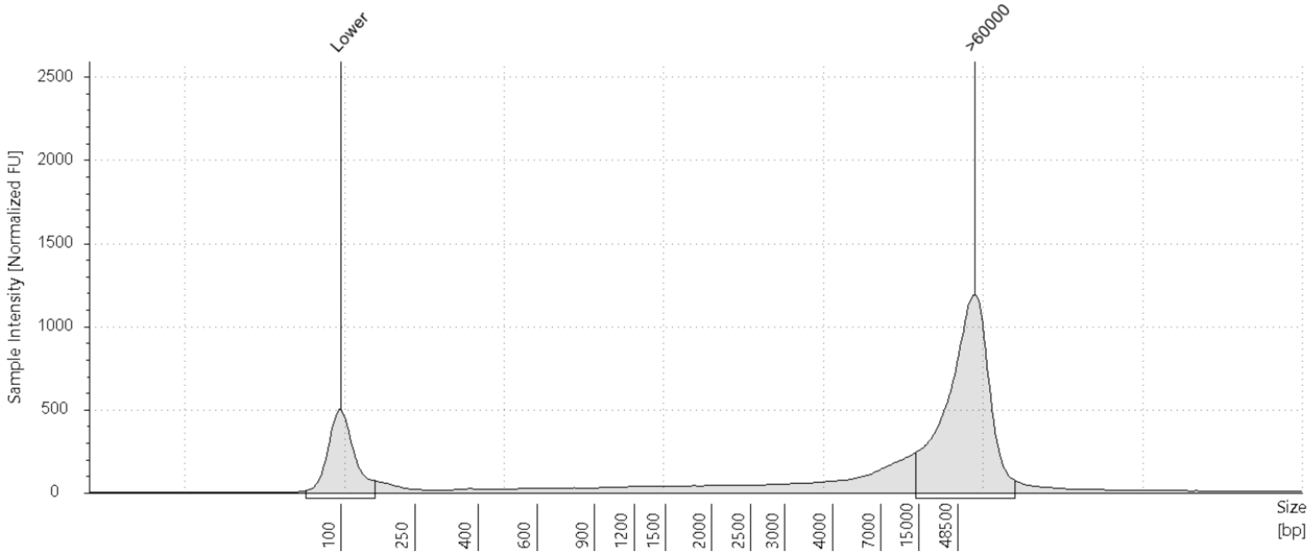

G1: [redacted] sample 6

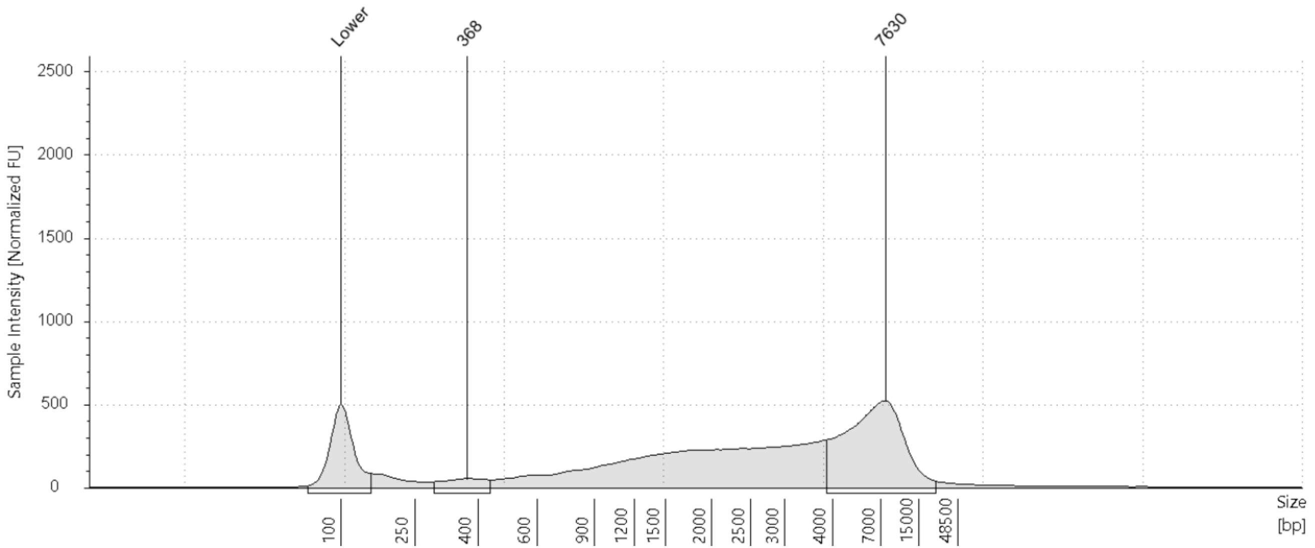

H1: [redacted] sample 7

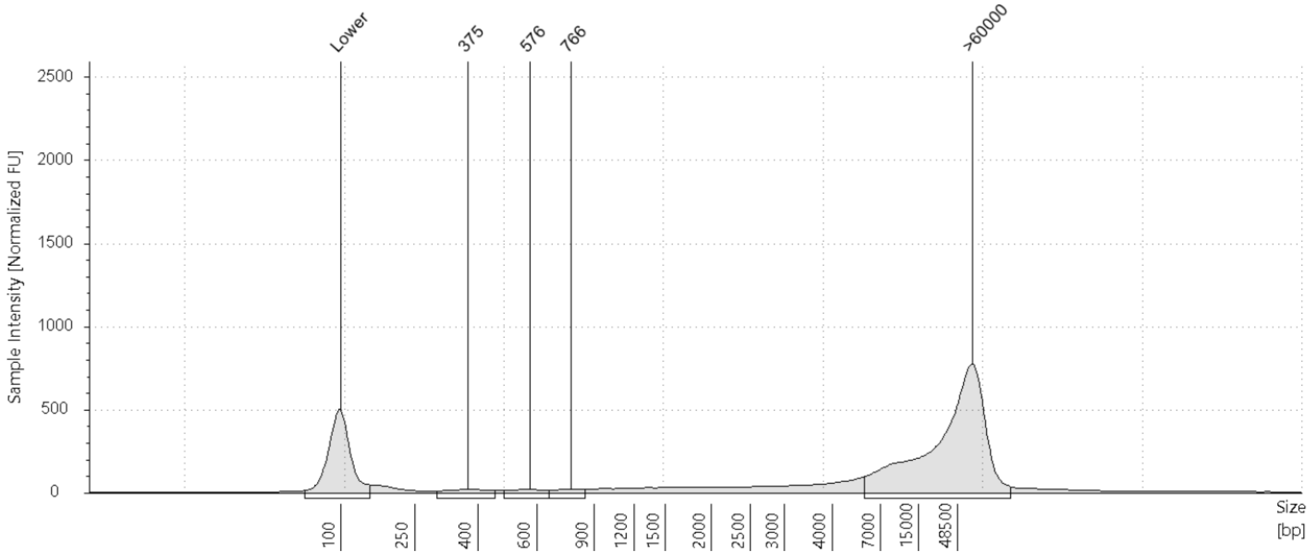

A2: [redacted] sample 8

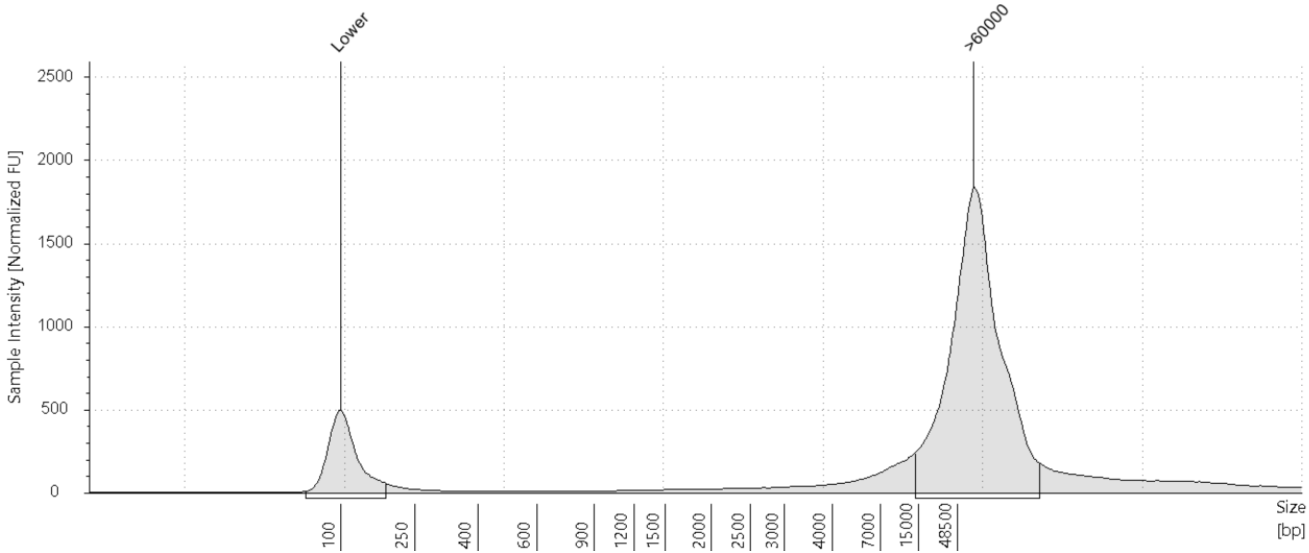

B2: [redacted] sample 9

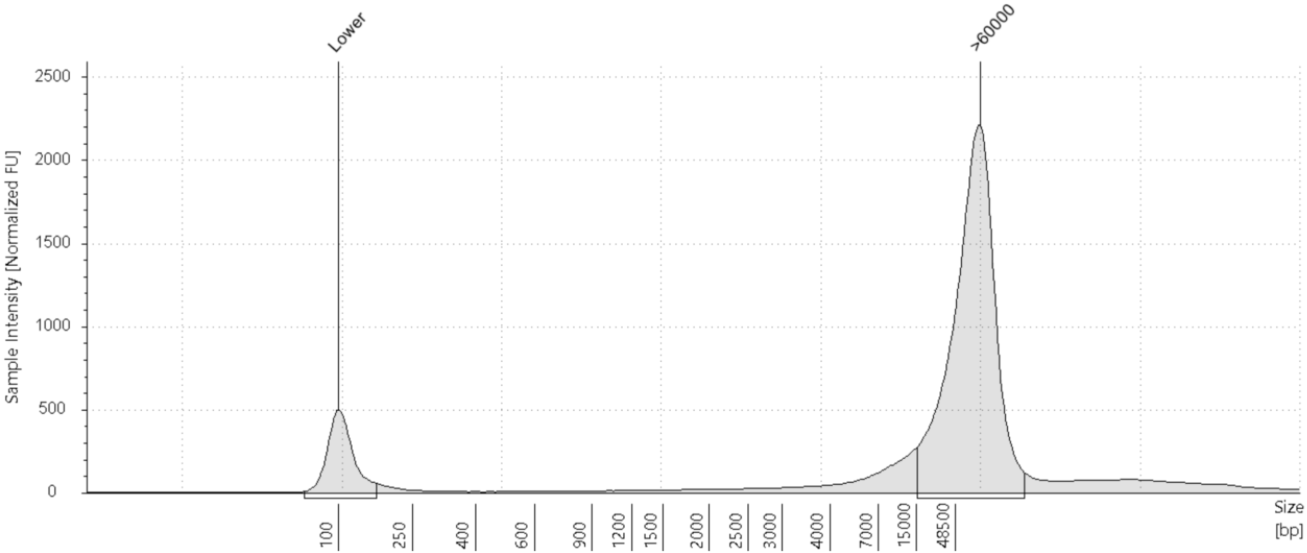

C2: [redacted] sample 10

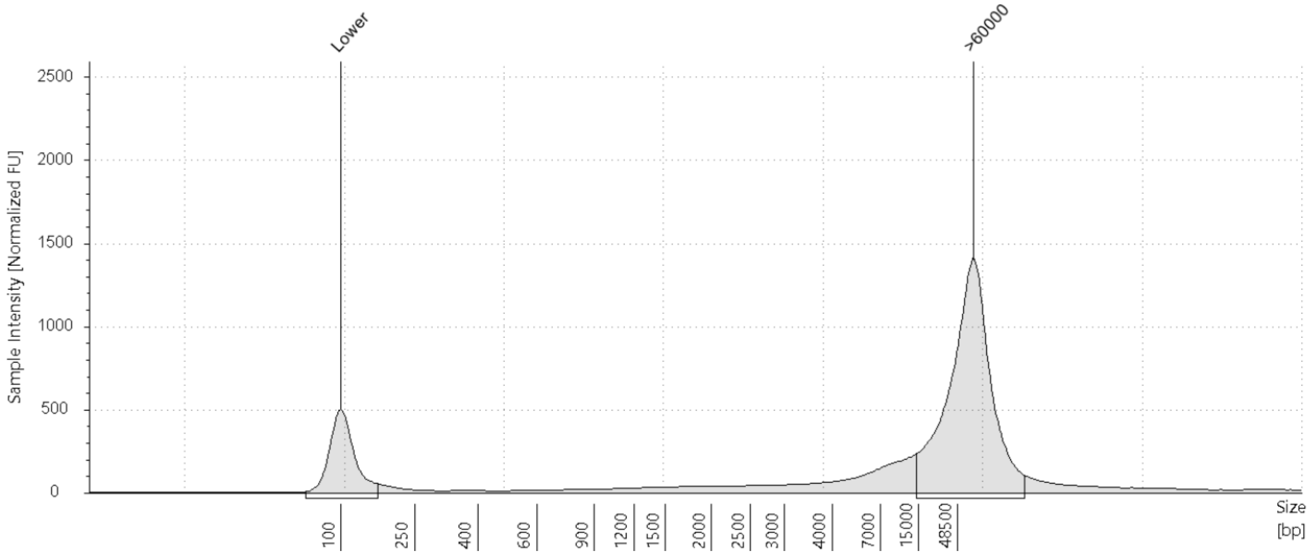

D2: [redacted] sample 11

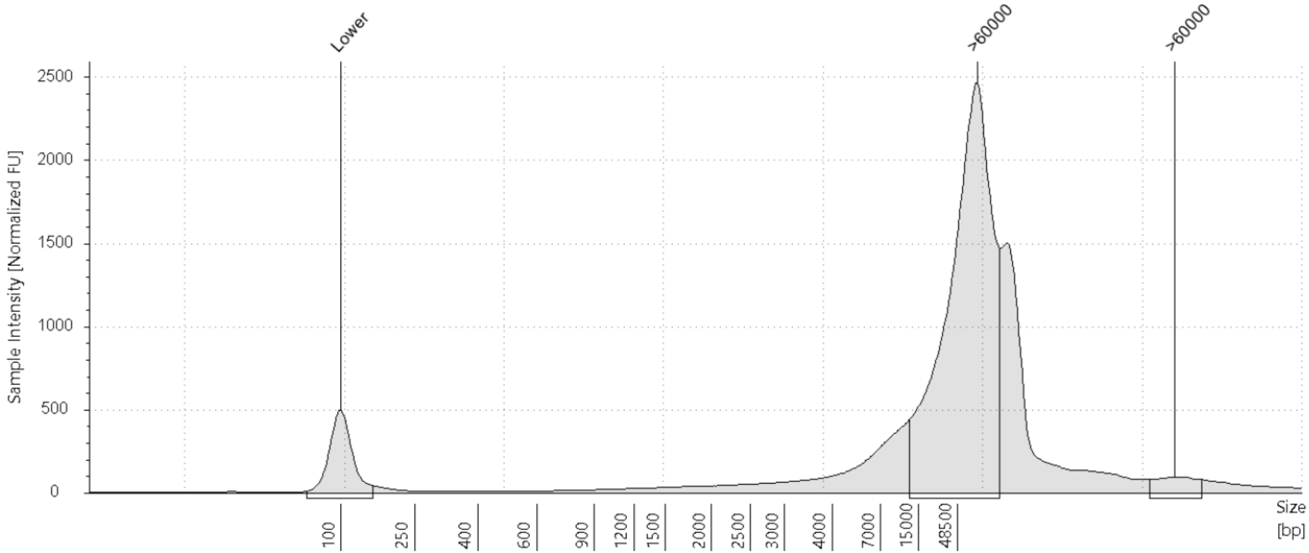

E2: [redacted] sample 12

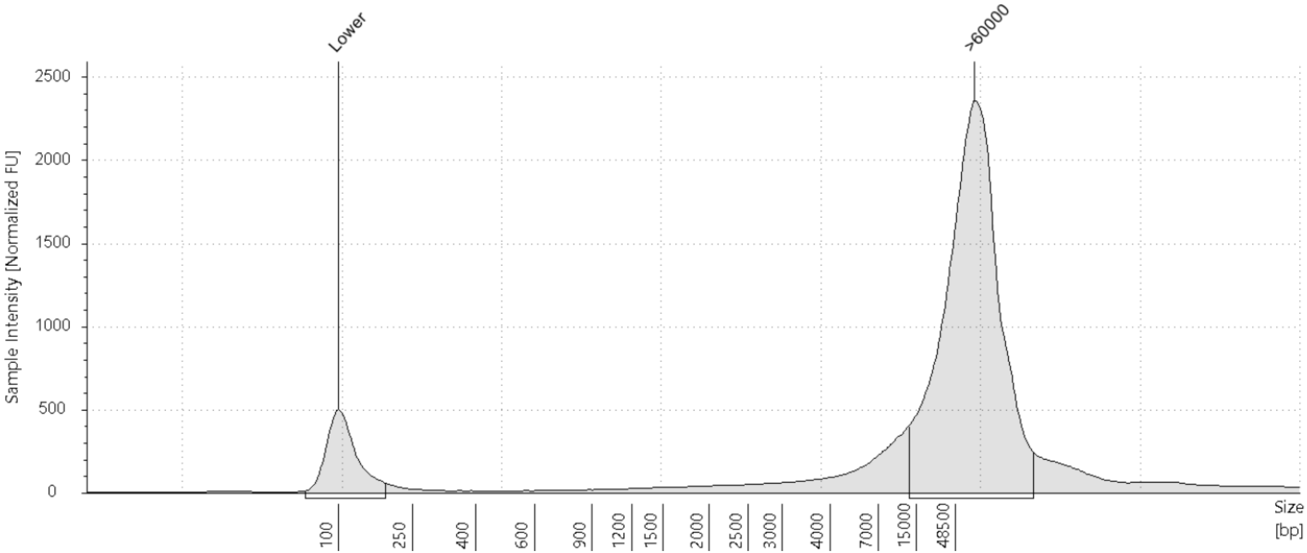

F2: [redacted] sample 13

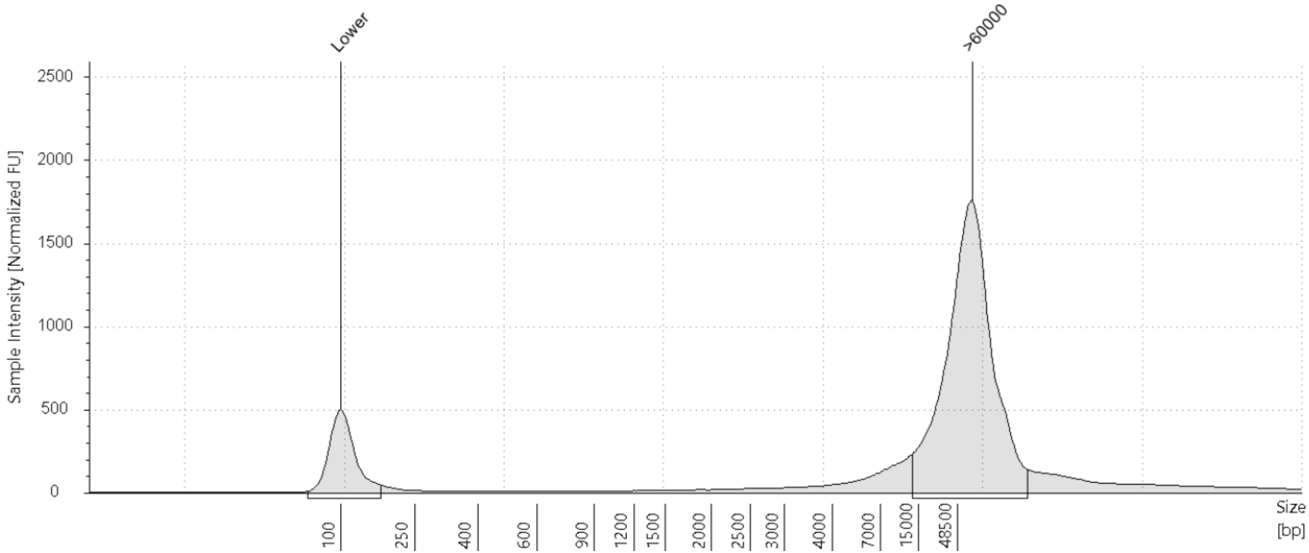

G2: [redacted] sample 14

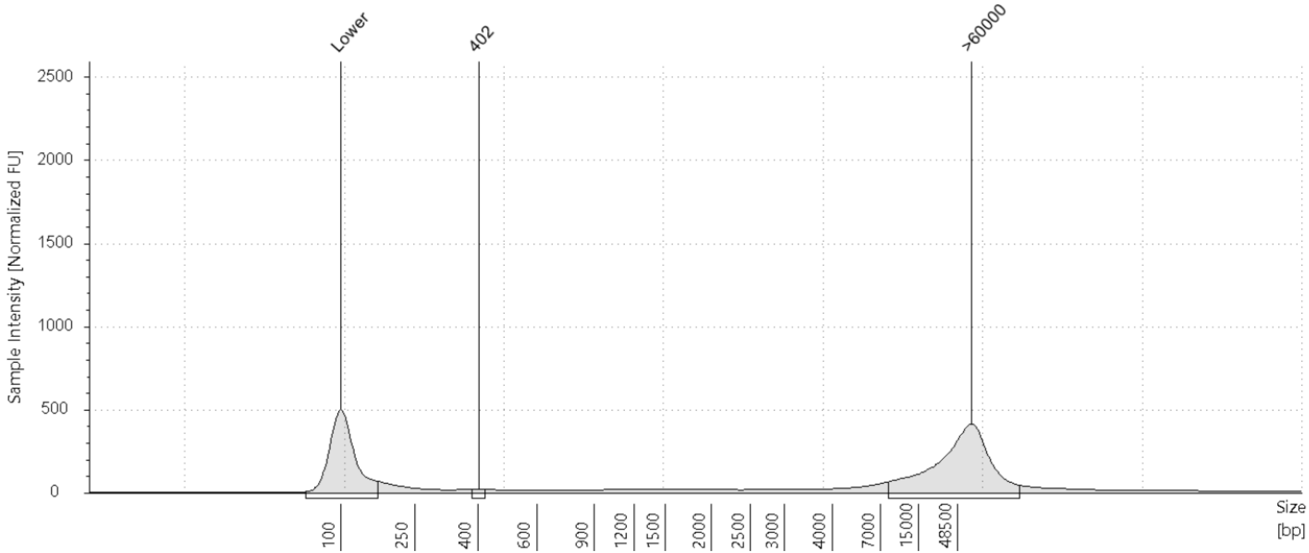

H2: [redacted] sample 15

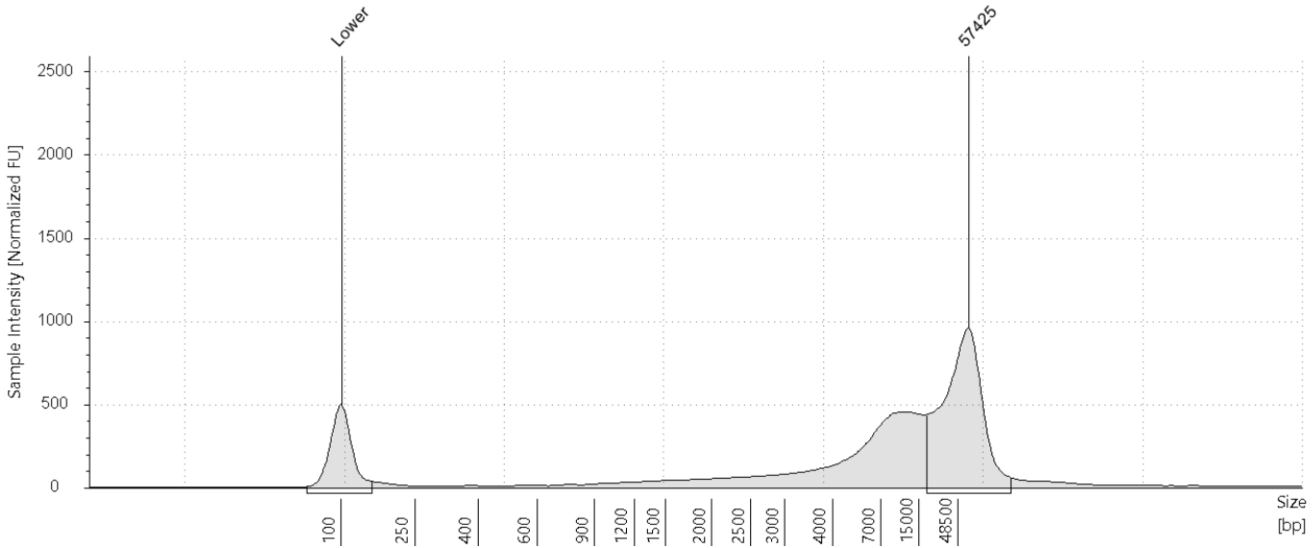

B1: [redacted] sample 16

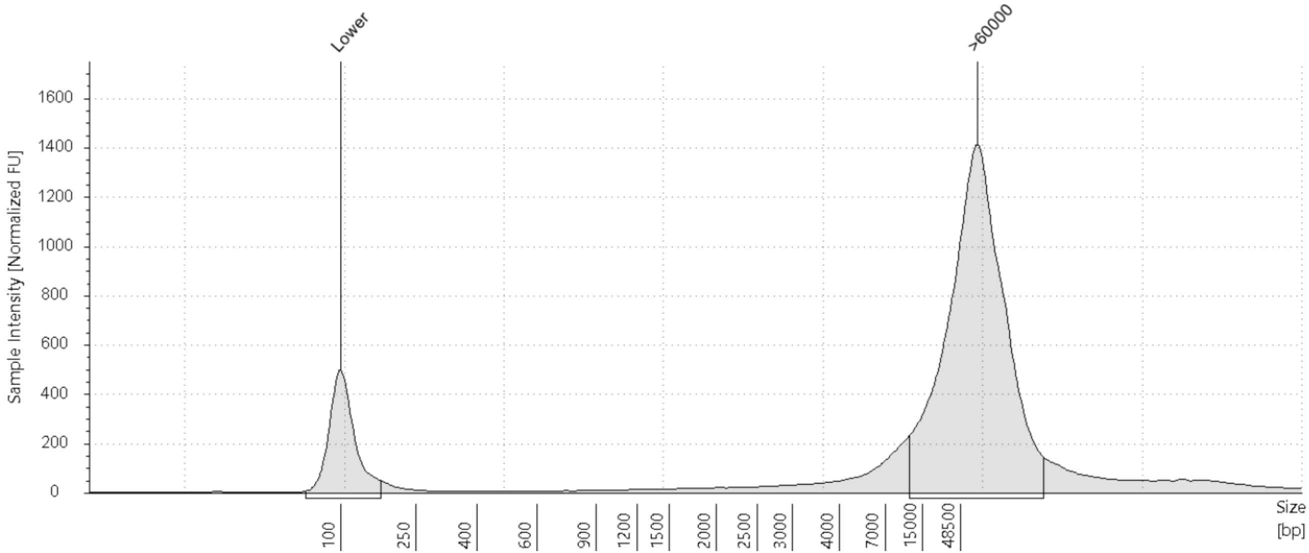

C1: [redacted] sample 17

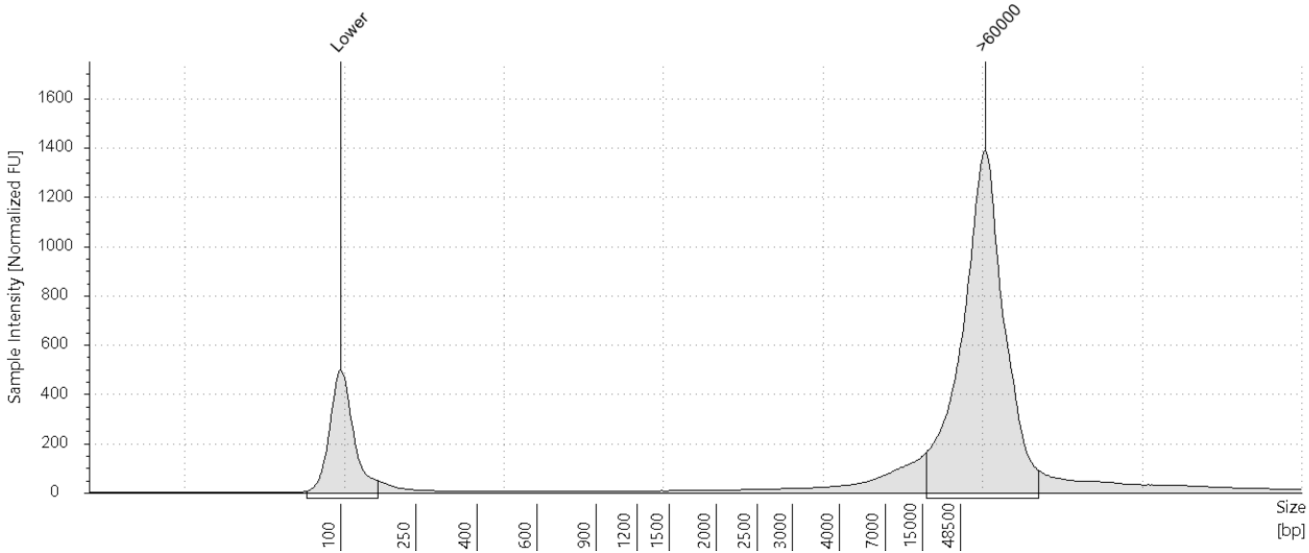

D1: [redacted] sample 18

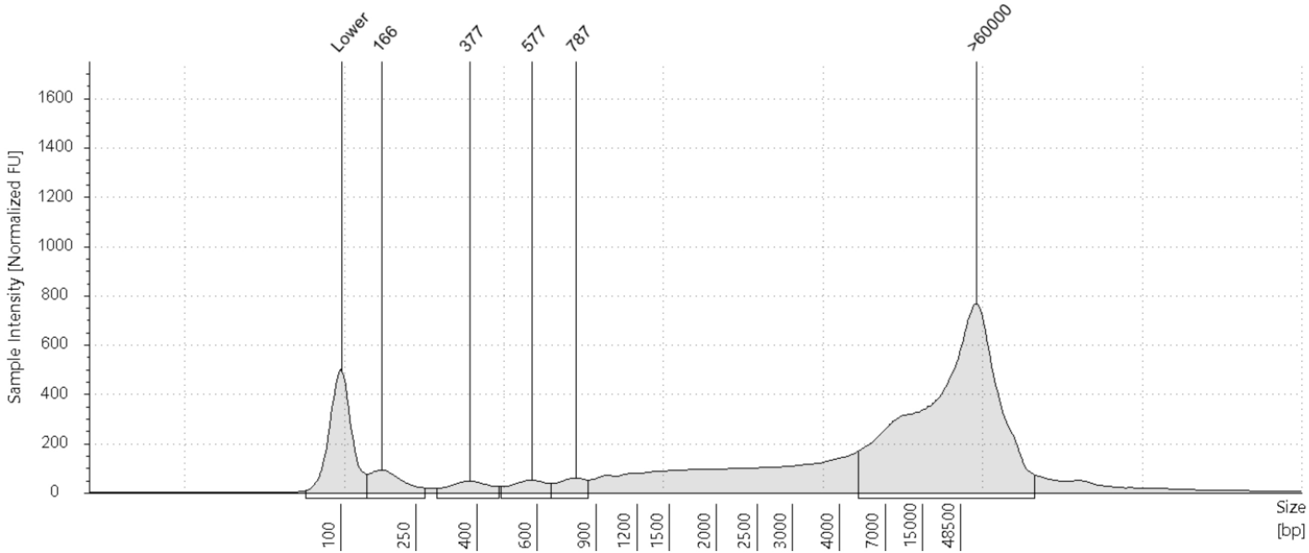

E1: [redacted] sample 19

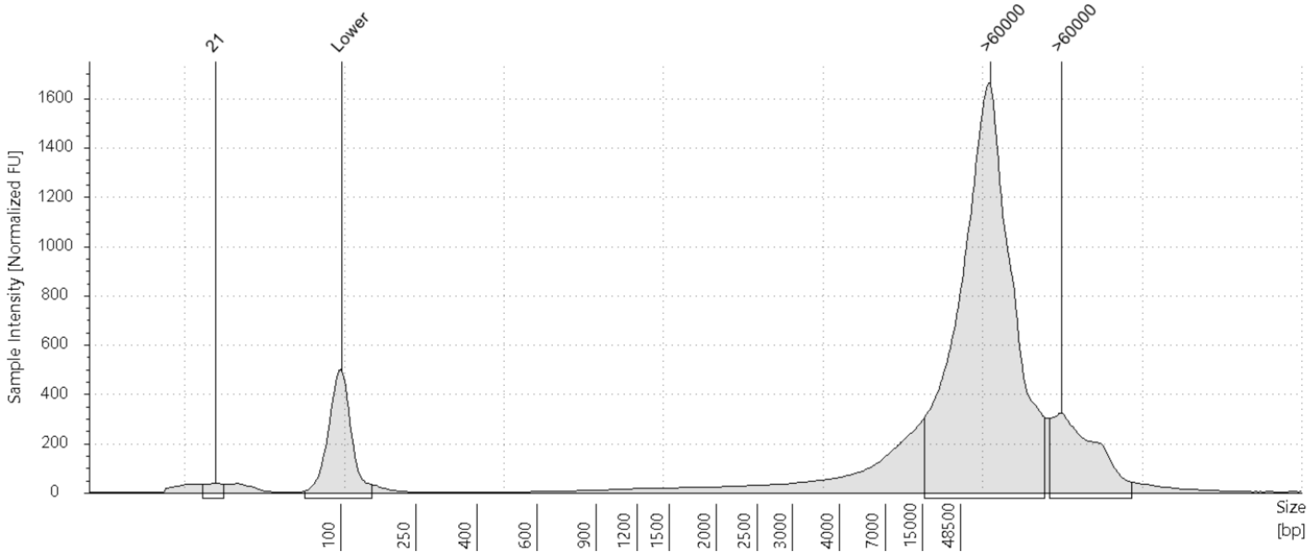

B1: [redacted] sample 20

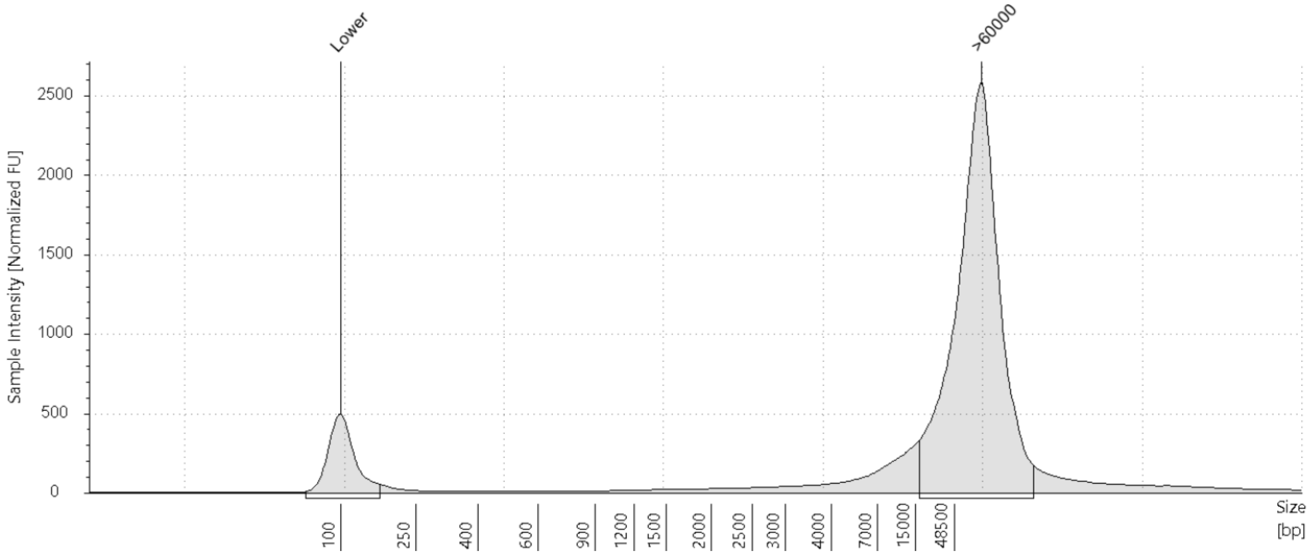

D1: [redacted] sample 21

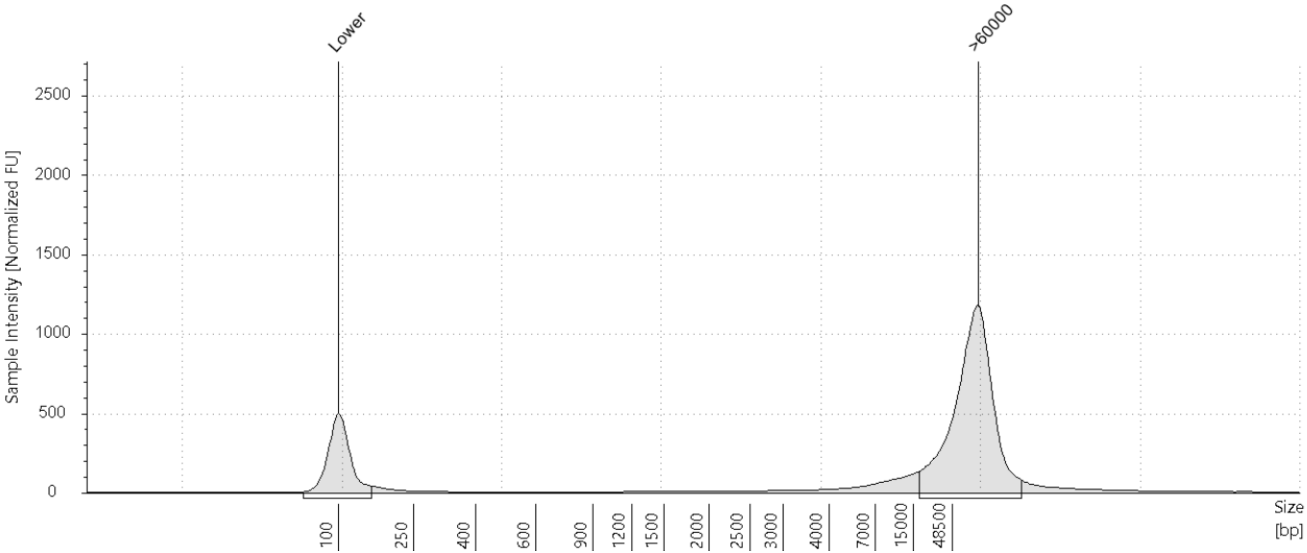

B1: [redacted] sample 22

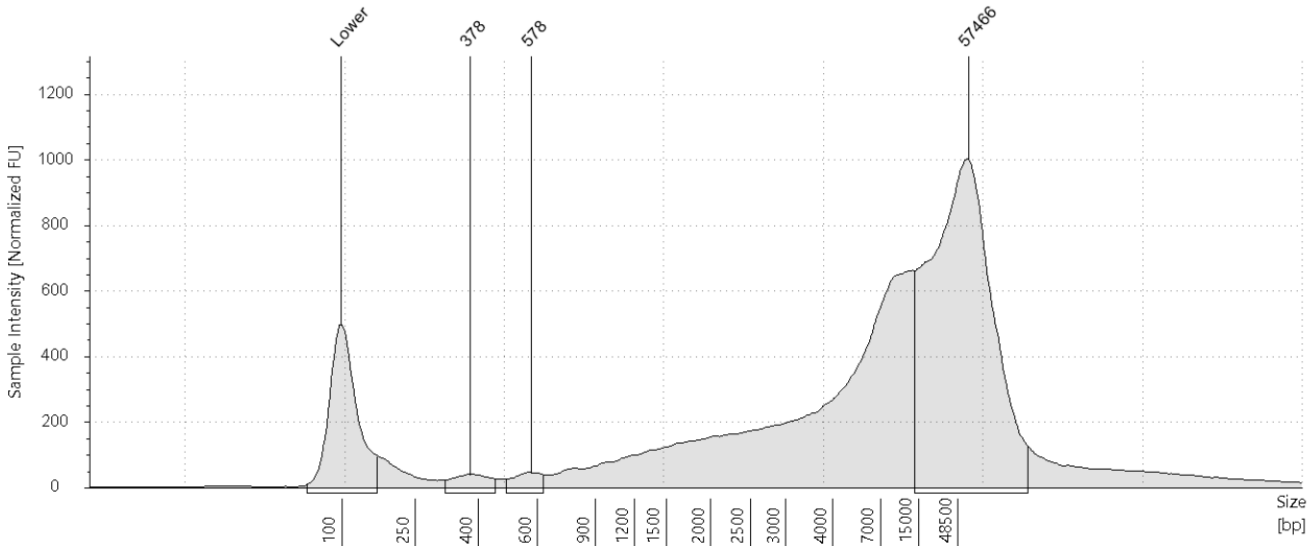

C1: [redacted] sample 23

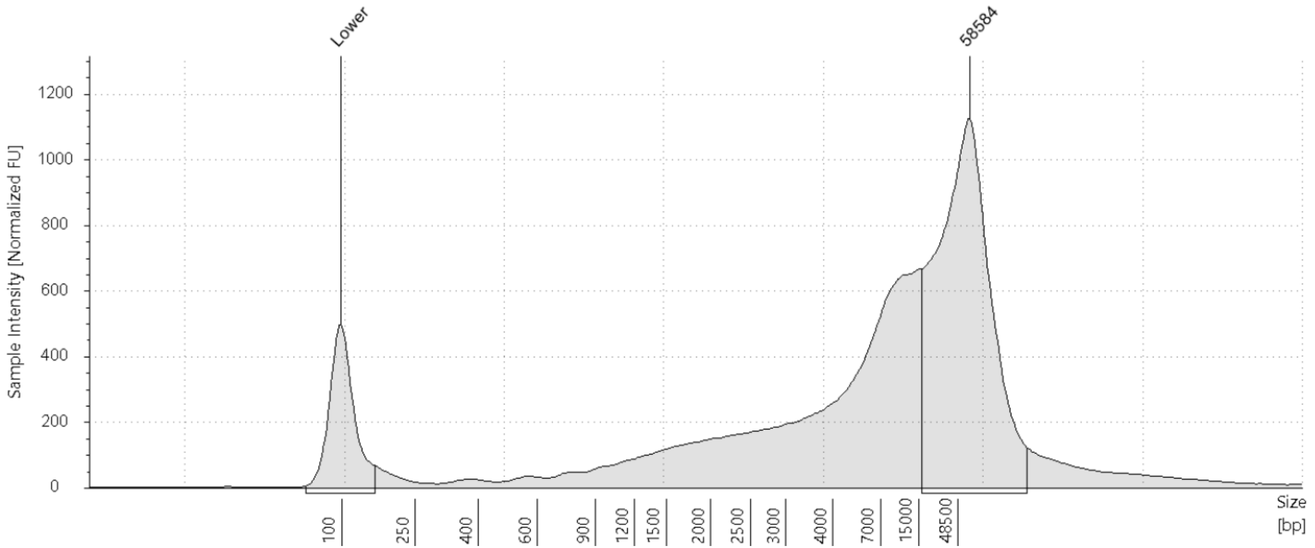

D1: [redacted] sample 24

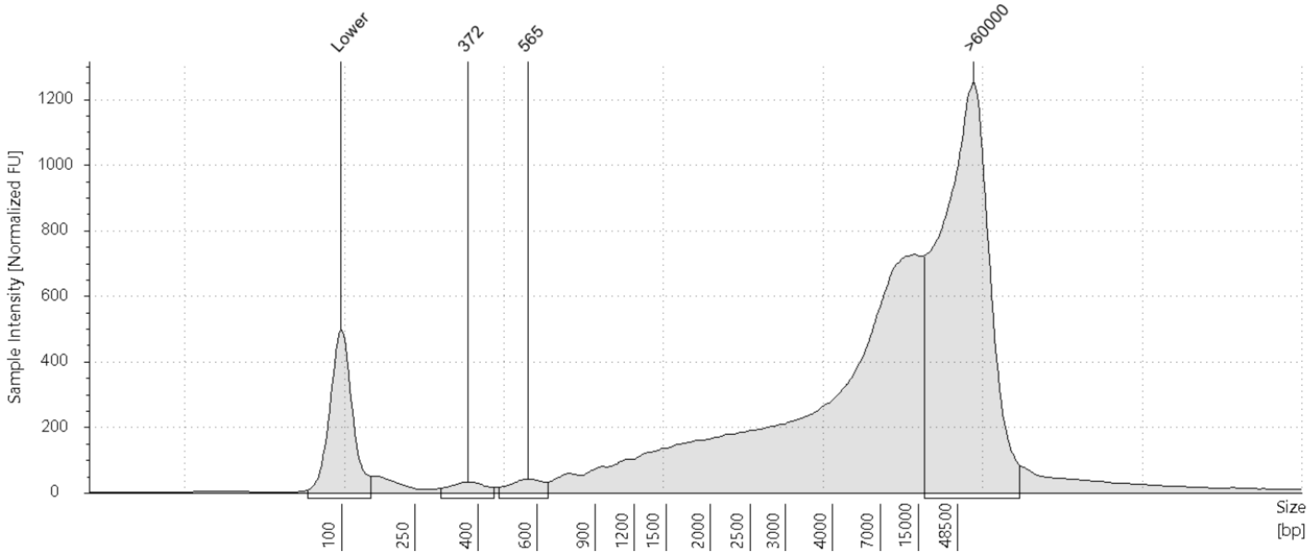

Supplement: Supplementary file 4 — Appendix S4 [file JCLA-38-e25029-s002.pdf]
