# Supplementary material for: Evaluation of Automated Magnetic Bead–Based DNA Extraction for Detection of Short Tandem Repeat Expansions With Nanopore Sequencing
Source: J Clin Lab Anal. 2024 Mar 20;38(6):e25029. doi: 10.1002/jcla.25029 (PMC10997813; doi:10.1002/jcla.25029)
Supplement: Supplementary file 5 — Appendix S5 [file JCLA-38-e25029-s006.html]

NanoComp Report

- Summary Statistics
- Plots
  - Comparing number of reads
  - Comparing throughput in bases
  - Comparing read length N50
  - Comparing read length
  - Comparing log-transformed read length
  - Comparing average base call quality score
  - Histogram of read lengths
  - Normalized histogram of read lengths
  - Weighted histogram of read lengths
  - Histogram of log transformed read lengths
  - Normalized histogram of log transformed read lengths
  - Weighted histogram of log transformed read lengths
- Report issue on Github

# NanoComp report

## Summary statistics

| feature |  |  |  |  |  |  |  |  |  |  |  |  |  |  |  |  |  |  |  |  |  |  |  |  |
| --- | --- | --- | --- | --- | --- | --- | --- | --- | --- | --- | --- | --- | --- | --- | --- | --- | --- | --- | --- | --- | --- | --- | --- | --- |
| General summary | NB01\_DIN\_9.2 | NB02\_DIN\_2.6 | NB03\_DIN\_7.4 | NB04\_DIN\_5.9 | NB05\_DIN\_7.9 | NB06\_DIN\_5.2 | NB07\_DIN\_7.6 | NB08\_DIN\_9.4 | NB09\_DIN\_9.4 | NB10\_DIN\_8.4 | NB11\_DIN\_9.1 | NB12\_DIN\_9.1 | NB13\_DIN\_9.3 | NB14\_DIN\_7.6 | NB15\_DIN\_7.5 | NB16\_DIN\_9.3 | NB17\_DIN\_9.6 | NB18\_DIN\_6.8 | NB19\_DIN\_9.3 | NB20\_DIN\_9.5 | NB21\_DIN\_9.6 | NB22\_DIN\_6.9 | NB23\_DIN\_7.0 | NB24\_DIN\_6.9 |
| Mean read length | 10,050.1 | 1,580.6 | 4,358.1 | 2,466.3 | 5,709.2 | 2,125.4 | 4,664.5 | 7,734.4 | 8,295.7 | 5,187.5 | 7,118.6 | 6,452.0 | 8,832.8 | 3,728.2 | 4,728.2 | 7,744.2 | 8,853.7 | 3,902.7 | 8,392.0 | 9,007.4 | 8,152.3 | 3,789.4 | 3,850.4 | 3,749.2 |
| Mean read quality | 16.0 | 16.7 | 16.3 | 16.5 | 16.2 | 16.5 | 16.3 | 16.1 | 16.2 | 16.3 | 16.1 | 16.2 | 16.1 | 16.2 | 16.2 | 16.2 | 16.1 | 16.3 | 16.0 | 16.2 | 16.1 | 16.3 | 16.2 | 16.2 |
| Median read length | 5,059.0 | 1,408.0 | 3,073.0 | 2,254.0 | 3,403.5 | 1,950.0 | 3,224.0 | 4,394.5 | 4,977.0 | 3,215.0 | 4,362.0 | 4,043.5 | 4,908.0 | 2,079.0 | 3,616.0 | 4,859.0 | 4,891.0 | 2,869.0 | 4,848.5 | 4,898.0 | 4,882.0 | 2,948.0 | 2,999.0 | 2,990.0 |
| Median read quality | 17.9 | 18.8 | 18.2 | 18.4 | 18.2 | 18.5 | 18.2 | 18.0 | 18.1 | 18.2 | 18.0 | 18.1 | 18.0 | 18.1 | 18.1 | 18.0 | 18.0 | 18.2 | 18.0 | 18.1 | 18.0 | 18.2 | 18.2 | 18.2 |
| Number of reads | 6,459.0 | 20,333.0 | 37,868.0 | 101,025.0 | 14,316.0 | 49,876.0 | 29,389.0 | 5,358.0 | 5,996.0 | 15,608.0 | 10,087.0 | 7,530.0 | 7,131.0 | 12,444.0 | 34,493.0 | 6,077.0 | 4,639.0 | 32,557.0 | 8,166.0 | 7,857.0 | 6,859.0 | 48,847.0 | 44,625.0 | 39,096.0 |
| Read length N50 | 20,316.0 | 1,603.0 | 5,171.0 | 2,708.0 | 8,282.0 | 2,319.0 | 5,687.0 | 12,189.0 | 12,808.0 | 6,785.0 | 10,178.0 | 9,061.0 | 15,290.0 | 5,562.0 | 5,560.0 | 11,705.0 | 15,192.0 | 4,450.0 | 13,779.0 | 15,681.0 | 12,910.0 | 4,283.0 | 4,346.0 | 4,224.0 |
| STDEV read length | 12,430.5 | 794.8 | 5,035.4 | 1,153.0 | 7,428.0 | 970.5 | 5,479.2 | 9,674.6 | 9,427.4 | 6,986.4 | 8,541.1 | 7,310.0 | 10,296.4 | 5,856.8 | 4,307.8 | 8,271.5 | 10,543.9 | 3,995.3 | 9,653.7 | 10,744.1 | 8,757.1 | 3,401.7 | 3,397.3 | 2,967.5 |
| Total bases | 64,913,909.0 | 32,137,581.0 | 165,031,509.0 | 249,158,630.0 | 81,732,247.0 | 106,005,779.0 | 137,086,163.0 | 41,440,687.0 | 49,741,282.0 | 80,965,841.0 | 71,805,165.0 | 48,583,437.0 | 62,986,553.0 | 46,393,559.0 | 163,091,212.0 | 47,061,742.0 | 41,072,254.0 | 127,060,317.0 | 68,528,818.0 | 70,771,154.0 | 55,916,712.0 | 185,101,201.0 | 171,823,856.0 | 146,579,837.0 |
| Number, percentage and megabases of reads above quality cutoffs |  |  |  |  |  |  |  |  |  |  |  |  |  |  |  |  |  |  |  |  |  |  |  |  |
| >Q5 | 6459 (100.0%) 64.9Mb | 20333 (100.0%) 32.1Mb | 37868 (100.0%) 165.0Mb | 101025 (100.0%) 249.2Mb | 14316 (100.0%) 81.7Mb | 49876 (100.0%) 106.0Mb | 29389 (100.0%) 137.1Mb | 5358 (100.0%) 41.4Mb | 5996 (100.0%) 49.7Mb | 15608 (100.0%) 81.0Mb | 10087 (100.0%) 71.8Mb | 7530 (100.0%) 48.6Mb | 7131 (100.0%) 63.0Mb | 12444 (100.0%) 46.4Mb | 34493 (100.0%) 163.1Mb | 6077 (100.0%) 47.1Mb | 4639 (100.0%) 41.1Mb | 32557 (100.0%) 127.1Mb | 8166 (100.0%) 68.5Mb | 7857 (100.0%) 70.8Mb | 6859 (100.0%) 55.9Mb | 48847 (100.0%) 185.1Mb | 44625 (100.0%) 171.8Mb | 39096 (100.0%) 146.6Mb |
| >Q7 | 6459 (100.0%) 64.9Mb | 20333 (100.0%) 32.1Mb | 37868 (100.0%) 165.0Mb | 101025 (100.0%) 249.2Mb | 14316 (100.0%) 81.7Mb | 49876 (100.0%) 106.0Mb | 29389 (100.0%) 137.1Mb | 5358 (100.0%) 41.4Mb | 5996 (100.0%) 49.7Mb | 15608 (100.0%) 81.0Mb | 10087 (100.0%) 71.8Mb | 7530 (100.0%) 48.6Mb | 7131 (100.0%) 63.0Mb | 12444 (100.0%) 46.4Mb | 34493 (100.0%) 163.1Mb | 6077 (100.0%) 47.1Mb | 4639 (100.0%) 41.1Mb | 32557 (100.0%) 127.1Mb | 8166 (100.0%) 68.5Mb | 7857 (100.0%) 70.8Mb | 6859 (100.0%) 55.9Mb | 48847 (100.0%) 185.1Mb | 44625 (100.0%) 171.8Mb | 39096 (100.0%) 146.6Mb |
| >Q10 | 6435 (99.6%) 64.5Mb | 20313 (99.9%) 32.1Mb | 37818 (99.9%) 164.8Mb | 100924 (99.9%) 248.8Mb | 14282 (99.8%) 81.3Mb | 49818 (99.9%) 105.9Mb | 29353 (99.9%) 136.9Mb | 5350 (99.9%) 41.3Mb | 5990 (99.9%) 49.7Mb | 15582 (99.8%) 80.8Mb | 10061 (99.7%) 71.6Mb | 7512 (99.8%) 48.5Mb | 7118 (99.8%) 62.9Mb | 12428 (99.9%) 46.3Mb | 34436 (99.8%) 162.7Mb | 6066 (99.8%) 46.9Mb | 4628 (99.8%) 41.0Mb | 32506 (99.8%) 126.9Mb | 8139 (99.7%) 68.2Mb | 7846 (99.9%) 70.7Mb | 6842 (99.8%) 55.8Mb | 48778 (99.9%) 184.6Mb | 44557 (99.8%) 171.3Mb | 39030 (99.8%) 146.3Mb |
| >Q12 | 6115 (94.7%) 60.9Mb | 19543 (96.1%) 30.8Mb | 36252 (95.7%) 157.1Mb | 96814 (95.8%) 238.1Mb | 13631 (95.2%) 76.9Mb | 47893 (96.0%) 101.5Mb | 28065 (95.5%) 130.4Mb | 5076 (94.7%) 38.8Mb | 5729 (95.5%) 47.5Mb | 14889 (95.4%) 77.1Mb | 9581 (95.0%) 67.8Mb | 7147 (94.9%) 46.0Mb | 6759 (94.8%) 59.5Mb | 11896 (95.6%) 43.7Mb | 32932 (95.5%) 155.0Mb | 5800 (95.4%) 44.9Mb | 4420 (95.3%) 39.2Mb | 31074 (95.4%) 120.6Mb | 7730 (94.7%) 64.0Mb | 7508 (95.6%) 67.3Mb | 6532 (95.2%) 52.8Mb | 46648 (95.5%) 175.8Mb | 42525 (95.3%) 162.5Mb | 37207 (95.2%) 138.9Mb |
| >Q15 | 5242 (81.2%) 51.5Mb | 17107 (84.1%) 26.9Mb | 31446 (83.0%) 134.5Mb | 84562 (83.7%) 207.2Mb | 11763 (82.2%) 65.3Mb | 41887 (84.0%) 88.3Mb | 24364 (82.9%) 111.7Mb | 4391 (82.0%) 33.3Mb | 4977 (83.0%) 41.1Mb | 12944 (82.9%) 66.3Mb | 8238 (81.7%) 57.9Mb | 6215 (82.5%) 39.4Mb | 5841 (81.9%) 51.1Mb | 10181 (81.8%) 36.1Mb | 28353 (82.2%) 132.3Mb | 5030 (82.8%) 38.8Mb | 3796 (81.8%) 33.2Mb | 26936 (82.7%) 103.9Mb | 6666 (81.6%) 54.7Mb | 6495 (82.7%) 57.6Mb | 5651 (82.4%) 45.8Mb | 40495 (82.9%) 151.9Mb | 36786 (82.4%) 140.1Mb | 32153 (82.2%) 119.4Mb |
| Top 5 highest mean basecall quality scores and their read lengths |  |  |  |  |  |  |  |  |  |  |  |  |  |  |  |  |  |  |  |  |  |  |  |  |
| 1 | 29.5 (2213) | 34.4 (1220) | 32.6 (1138) | 33.9 (1093) | 32.7 (1573) | 33.9 (1324) | 33.0 (1419) | 28.3 (1012) | 30.1 (1461) | 33.8 (1639) | 30.2 (1775) | 31.3 (1521) | 29.5 (1598) | 34.1 (1257) | 32.5 (1287) | 31.1 (1433) | 28.8 (1284) | 33.1 (1159) | 31.1 (1719) | 30.6 (1025) | 29.9 (3432) | 31.8 (1043) | 33.9 (1180) | 34.1 (1843) |
| 2 | 28.6 (1628) | 32.9 (1510) | 32.1 (2340) | 33.7 (1452) | 31.9 (1010) | 32.5 (1460) | 32.8 (1213) | 27.9 (2176) | 29.9 (3210) | 31.7 (1889) | 29.4 (5217) | 29.9 (2920) | 29.0 (1188) | 31.2 (1114) | 31.6 (1900) | 30.9 (1098) | 28.7 (4196) | 31.8 (1328) | 29.8 (1292) | 30.3 (1677) | 28.8 (2187) | 31.4 (1112) | 31.9 (1165) | 32.9 (1141) |
| 3 | 27.6 (2330) | 32.6 (1056) | 32.0 (1090) | 32.9 (1520) | 28.9 (2207) | 32.0 (1258) | 31.7 (1645) | 27.9 (4208) | 29.8 (2243) | 31.0 (1050) | 29.3 (1377) | 29.7 (2613) | 28.8 (1441) | 30.9 (1181) | 30.9 (1293) | 28.7 (3461) | 28.1 (2149) | 31.7 (1694) | 29.0 (3154) | 28.9 (2465) | 28.7 (1292) | 31.0 (2742) | 31.8 (1252) | 31.6 (1223) |
| 4 | 26.9 (3655) | 32.5 (1152) | 31.3 (2185) | 32.5 (1040) | 28.4 (5516) | 32.0 (1169) | 31.4 (1704) | 27.8 (3115) | 28.9 (2504) | 30.6 (1398) | 29.2 (5154) | 29.0 (1800) | 28.4 (1162) | 30.8 (1014) | 30.7 (2035) | 28.2 (2637) | 26.9 (1151) | 31.7 (1144) | 28.6 (1201) | 28.6 (2882) | 28.2 (4174) | 30.9 (1235) | 31.6 (4081) | 31.5 (1982) |
| 5 | 26.2 (2899) | 32.2 (2147) | 30.9 (1374) | 32.5 (1502) | 28.4 (1292) | 31.9 (1045) | 30.8 (2121) | 27.6 (1634) | 27.8 (1997) | 30.4 (1217) | 29.1 (2609) | 28.8 (1519) | 28.0 (3879) | 30.8 (1811) | 30.7 (1366) | 27.9 (1510) | 26.9 (6454) | 31.4 (1885) | 28.5 (2086) | 28.5 (1665) | 27.5 (2398) | 30.7 (1141) | 31.5 (1039) | 31.2 (1022) |
| Top 5 longest reads and their mean basecall quality score |  |  |  |  |  |  |  |  |  |  |  |  |  |  |  |  |  |  |  |  |  |  |  |  |
| 1 | 99919 (16.5) | 45112 (17.9) | 127732 (10.2) | 29283 (12.6) | 169051 (11.5) | 39486 (18.9) | 143305 (11.0) | 126057 (12.1) | 98165 (19.1) | 166681 (14.2) | 91043 (17.5) | 79918 (13.7) | 129168 (12.1) | 102687 (13.7) | 94696 (12.8) | 81108 (11.0) | 90983 (17.0) | 70429 (17.5) | 102421 (14.4) | 97228 (18.5) | 93371 (17.4) | 102531 (10.1) | 94018 (9.8) | 61233 (18.7) |
| 2 | 96221 (17.6) | 38128 (16.7) | 92053 (15.3) | 27482 (17.1) | 98324 (11.8) | 30793 (11.6) | 109005 (18.4) | 98335 (18.3) | 87205 (19.1) | 88953 (20.5) | 86815 (10.1) | 77524 (17.2) | 80618 (19.4) | 99479 (15.0) | 82272 (11.9) | 77322 (20.0) | 81966 (10.8) | 69070 (13.3) | 83634 (16.5) | 93413 (13.3) | 80492 (11.9) | 101749 (9.9) | 91447 (18.4) | 59545 (15.1) |
| 3 | 95332 (18.5) | 30491 (10.7) | 91982 (18.3) | 27124 (12.7) | 87075 (18.7) | 29276 (15.6) | 103540 (19.1) | 96261 (10.4) | 81965 (21.0) | 85999 (16.9) | 83778 (18.6) | 75944 (10.5) | 79744 (18.7) | 93080 (12.9) | 78915 (14.4) | 75791 (17.9) | 77157 (13.2) | 65487 (17.8) | 82996 (17.0) | 91384 (18.3) | 75574 (10.6) | 75821 (16.8) | 70295 (11.0) | 59411 (15.6) |
| 4 | 93645 (12.8) | 29986 (19.0) | 90210 (12.6) | 21853 (14.0) | 80706 (18.8) | 24309 (14.5) | 97401 (10.6) | 94819 (12.4) | 80294 (13.5) | 85301 (15.1) | 83761 (11.0) | 69455 (17.2) | 76026 (19.6) | 83584 (14.0) | 74782 (18.4) | 74519 (19.8) | 76404 (12.7) | 64115 (18.6) | 74617 (15.8) | 91008 (20.4) | 62550 (15.9) | 74422 (16.0) | 66870 (19.1) | 48361 (18.4) |
| 5 | 91025 (15.9) | 19969 (18.4) | 84411 (12.9) | 21528 (19.9) | 78067 (13.2) | 20087 (10.1) | 94111 (14.6) | 83483 (12.3) | 79949 (18.2) | 83068 (14.0) | 83730 (13.6) | 66770 (16.4) | 75356 (10.4) | 77901 (17.7) | 70788 (11.4) | 73058 (22.2) | 70748 (18.3) | 63307 (18.3) | 74097 (18.4) | 86861 (13.0) | 62392 (14.3) | 73260 (10.5) | 65815 (10.9) | 46910 (10.0) |

## Plots

Comparing number of reads

#### Comparing number of reads

Comparing throughput in bases

#### Comparing throughput in bases

Comparing read length N50

#### Comparing read length N50

Comparing read length

#### Comparing read length

Comparing log-transformed read length

#### Comparing log-transformed read length

Comparing average base call quality score

#### Comparing average base call quality score

Histogram of read lengths

#### Histogram of read lengths

Normalized histogram of read lengths

#### Normalized histogram of read lengths

Weighted histogram of read lengths

#### Weighted histogram of read lengths

Histogram of log transformed read lengths

#### Histogram of log transformed read lengths

Normalized histogram of log transformed read lengths

#### Normalized histogram of log transformed read lengths

Weighted histogram of log transformed read lengths

#### Weighted histogram of log transformed read lengths
