# Supplementary material for: Evaluation of Automated Magnetic Bead–Based DNA Extraction for Detection of Short Tandem Repeat Expansions With Nanopore Sequencing
Source: J Clin Lab Anal. 2024 Mar 20;38(6):e25029. doi: 10.1002/jcla.25029 (PMC10997813; doi:10.1002/jcla.25029)
Supplement: Supplementary file 6 — Appendix S6 [file JCLA-38-e25029-s004.html]

NanoComp Report

- Summary Statistics
- Plots
  - Comparing number of reads
  - Comparing throughput in bases
  - Comparing read length N50
  - Comparing read length
  - Comparing log-transformed read length
  - Comparing average base call quality score
  - Comparing percent reference identity
  - Histogram of percent reference identity
  - Histogram of Phred scores
  - Histogram of read lengths
  - Normalized histogram of read lengths
  - Weighted histogram of read lengths
  - Histogram of log transformed read lengths
  - Normalized histogram of log transformed read lengths
  - Weighted histogram of log transformed read lengths
- Report issue on Github

# NanoComp report

## Summary statistics

| feature |  |  |  |
| --- | --- | --- | --- |
| General summary | FXN | C9orf72 | CNBP |
| Average percent identity | 95.9 | 97.5 | 96.1 |
| Fraction of bases aligned | 1.0 | 0.9 | 1.0 |
| Mean read length | 1,502.4 | 891.6 | 1,025.5 |
| Mean read quality | 14.0 | 14.8 | 13.9 |
| Median percent identity | 96.8 | 98.2 | 96.9 |
| Median read length | 1,100.0 | 440.0 | 1,013.0 |
| Median read quality | 15.2 | 16.1 | 15.2 |
| Number of reads | 8,976,179.0 | 7,172,584.0 | 21,254,960.0 |
| Read length N50 | 1,958.0 | 1,202.0 | 1,235.0 |
| STDEV read length | 1,442.6 | 2,684.6 | 630.6 |
| Total bases | 13,485,986,518.0 | 6,394,864,134.0 | 21,796,814,640.0 |
| Total bases aligned | 13,291,533,596.0 | 6,067,493,270.0 | 21,447,653,838.0 |
| Number, percentage and megabases of reads above quality cutoffs |  |  |  |
| >Q5 | 8976139 (100.0%) 13486.0Mb | 7172527 (100.0%) 6394.8Mb | 21254937 (100.0%) 21796.8Mb |
| >Q7 | 8972748 (100.0%) 13484.3Mb | 7171741 (100.0%) 6394.1Mb | 21252771 (100.0%) 21796.1Mb |
| >Q10 | 8927897 (99.5%) 13461.4Mb | 7162608 (99.9%) 6385.5Mb | 21209047 (99.8%) 21781.4Mb |
| >Q12 | 7861806 (87.6%) 11846.2Mb | 6693969 (93.3%) 6015.3Mb | 18482550 (87.0%) 18953.1Mb |
| >Q15 | 4795766 (53.4%) 7167.1Mb | 4732501 (66.0%) 4455.3Mb | 11183557 (52.6%) 11354.4Mb |
| Top 5 highest mean basecall quality scores and their read lengths |  |  |  |
| 1 | 30.1 (55; efc3c9a5-58a4-4f31-b2bd-d77a2346bc4b) | 31.5 (54; f561eb2e-2fa9-4637-a3cf-8ffb2c7db19b) | 30.7 (79; 6b960d60-e5c9-4e86-ae70-5f7bc128df5d) |
| 2 | 29.9 (80; 3d449365-0b56-49b1-acd5-1b31b2f1eac7) | 31.4 (80; af37324d-a841-47cb-b722-51bb71e14d75) | 30.2 (210; b09169f5-5e3f-44b1-b1c6-0aa583131231) |
| 3 | 29.6 (171; 12eeb628-5db5-4526-bfd8-4690ced32d64) | 31.2 (44; 496aad62-e579-4887-b376-ac9d84e60ae1) | 30.0 (135; dd484d31-7da5-48b0-a63e-012a0c80ed2d) |
| 4 | 29.6 (53; db458dfe-724a-48f0-b83a-a70a72edebdd) | 31.1 (53; 2d730364-fd24-4b46-9de6-647a04dde8fd) | 30.0 (204; d4ab8c40-6602-4a50-aa2d-ea36ca046cf2) |
| 5 | 29.4 (165; 3e8376c6-6668-41eb-9578-1faff8df68b0) | 30.9 (62; 0deadf4c-44ac-4ef6-bf88-3b71ef4e9552) | 30.0 (60; 59049b0f-62a2-459e-a210-d85d3bd468fe) |
| Top 5 longest reads and their mean basecall quality score |  |  |  |
| 1 | 203557 (14.0; 42bf37c4-1f66-4bb9-bde5-f037f77b13a3) | 160898 (17.0; b5ef43ae-7d39-4fa6-b6e5-796f3449ba9e) | 101623 (14.2; f6ddda1f-1ad7-49b2-97a0-59258578e7f5) |
| 2 | 187412 (13.5; 1a5a7bd1-a032-4469-9302-0f01ad50aef6) | 154526 (13.4; 748df3aa-f2c0-4156-bff9-f5301babbef1) | 99134 (11.2; 363ff07c-f68c-4a43-9003-ab2af1fbe011) |
| 3 | 159473 (14.5; 0605e52a-67f4-469d-9963-e5ac00058c16) | 141975 (12.9; 92fcf4b9-08bd-4bec-9ea6-b85c94f3ecc1) | 95090 (11.9; f6410087-9aec-4e5d-83b5-a783383b3b39) |
| 4 | 111998 (10.4; e82f04cc-f39e-4b78-be11-d747168b8ef1) | 138372 (17.4; 3190a543-9951-4bd6-8a14-0ff64cfacd0e) | 89023 (13.4; 4fa5e794-b97a-4119-bc8b-abebd73ac83b) |
| 5 | 111364 (11.9; e056966a-79af-4a59-b41e-70da2fe0dfea) | 128219 (15.0; 39f8434a-8eca-405c-9c93-5802fdf50f98) | 85804 (14.7; 0ec94f3c-c86b-4d2f-9044-ff520a92febc) |

## Plots

Comparing number of reads

#### Comparing number of reads

Comparing throughput in bases

#### Comparing throughput in bases

Comparing read length N50

#### Comparing read length N50

Comparing read length

#### Comparing read length

Comparing log-transformed read length

#### Comparing log-transformed read length

Comparing average base call quality score

#### Comparing average base call quality score

Comparing percent reference identity

#### Comparing percent reference identity

Histogram of percent reference identity

#### Histogram of percent reference identity

Histogram of Phred scores

#### Histogram of Phred scores

Histogram of read lengths

#### Histogram of read lengths

Normalized histogram of read lengths

#### Normalized histogram of read lengths

Weighted histogram of read lengths

#### Weighted histogram of read lengths

Histogram of log transformed read lengths

#### Histogram of log transformed read lengths

Normalized histogram of log transformed read lengths

#### Normalized histogram of log transformed read lengths

Weighted histogram of log transformed read lengths

#### Weighted histogram of log transformed read lengths
